# National trends in the prevalence of hypertension and diabetes stratified by sleep duration in South Korea, 2009–2022: A nationwide cross-sectional study: Trends and association of sleep duration in hypertension and diabetes

**DOI:** 10.1097/MD.0000000000046042

**Published:** 2025-11-21

**Authors:** Jinyoung Jeong, Hyunjee Kim, Jaeyu Park, Hyeon Jin Kim, Hyesu Jo, Sooji Lee, Kyeongmin Lee, Seoyoung Park, Yesol Yim, Ho Geol Woo, Yejun Son, Soeun Kim, Sang Youl Rhee, Dong Keon Yon

**Affiliations:** a Center for Digital Health, Medical Science Research Institute, Kyung Hee University Medical Center, Kyung Hee University College of Medicine, Seoul, South Korea; b Department of Medicine, Kyung Hee University College of Medicine, Seoul, South Korea; c Department of Precision Medicine, Kyung Hee University College of Medicine, Seoul, South Korea; d Department of Regulatory Science, Kyung Hee University, Seoul, South Korea; e Department of Neurology, Kyung Hee University Medical Center, Kyung Hee University College of Medicine, Seoul, South Korea; f Department of Endocrinology and Metabolism, Kyung Hee University Medical Center, Kyung Hee University College of Medicine, Seoul, South Korea; g Department of Pediatrics, Kyung Hee University College of Medicine, Seoul, South Korea.

**Keywords:** diabetes, hypertension, sleep duration, trend

## Abstract

The increase in sleep-related issues is associated with various health problems, particularly hypertension and diabetes. Therefore, this study aims to address these gaps in the literature by analyzing the relationship between sleep duration and the prevalence of hypertension and diabetes. This study analyzed the association between hypertension, diabetes, and sleep duration using the Korea Community Health Survey database from 2009 to 2022. The study used weighted linear regression models to identify trends in diseases and sleep duration. Additionally, this analysis employed weighted logistic regression to examine associations between diseases and covariates, presented as weighted odds ratio (wOR) and 95% confidence intervals (CIs). Our analysis included 2,903,887 individuals aged ≥19 years. The prevalence of individuals with both hypertension and diabetes increased before (β, 0.87 [95% CI, 0.83–0.90]) and during the COVID-19 pandemic (β, 0.64 [0.58–0.70]). This trend was particularly notable among individuals who sleep less than 6 hours, with an increase identified both before (β, 1.28 [1.16–1.40]) and during the pandemic (β, 1.21 [1.03–1.39]). The higher risk of having both conditions was found in individuals who slept <6 hours (wOR, 1.20 [95% CI, 1.18–1.23]) and >8 hours (wOR, 1.19 [1.17–1.22]) compared to those who slept 7 to 8 hours. Our study is the first to analyze the association between sleep duration and hypertension and diabetes by utilizing a large-scale dataset and conducting a comparative analysis between the pre-COVID-19 and COVID-19 periods. This study shows that inadequate sleep is associated with an increased risk of hypertension and diabetes.

## 1. Introduction

Night work and inadequate rest time due to a heavy workload have led to various sleep-related issues.^[1,2]^ In particular, reduced physical activity and increased screen time during the COVID-19 pandemic have exacerbated these sleep problems.^[3,4]^ These disturbances are particularly concerning because they can negatively impact overall health, affecting everything from cognitive function to the onset of various diseases, including cardiovascular, respiratory, and immune system disorders.^[5]^ Moreover, recent studies emphasized the urgent need to address sleep-related issues, uncovering the association between sleep disturbances and the onset of hypertension and diabetes.^[6]^ In particular, in Korea, the prevalence of diabetes and hypertension is as high as 15.5% and 30%, respectively, highlighting the growing need for in-depth research on this issue among the Korean population.^[7]^ Given these challenges, developing a comprehensive understanding of the associations between sleep duration, a type of sleep-related issue, and hypertension and diabetes is essential.

Despite the importance of this issue, previous studies examining the relationship between sleep duration and hypertension or diabetes have several limitations. Many of these studies did not conduct long-term time-series analyses encompassing the COVID-19 pandemic, and the findings regarding the association between short or long sleep duration and metabolic diseases have been inconsistent.^[8,9]^ Furthermore, although the comorbidity of hypertension and diabetes is of particular importance given its association with increased risk of other complications and the added complexity of disease management, prior studies did not examine the impact of sleep duration on individuals with both conditions.^[10]^ Moreover, there has been insufficient investigation into how sleep duration influences risk factors known to contribute to hypertension or diabetes.

Therefore, this study aims to address these research gaps and enhance our understanding of the association between sleep duration and the risk of hypertension and diabetes. Furthermore, this research will explore how sleep duration may influence the coexistence of hypertension and diabetes. Findings from the present study will likely provide evidence to advocate for including sleep health in National Health Policies, Strategies, and Plans, and recommend further investigation into the influence of sleep duration on hypertension and diabetes.

## 2. Materials and methods

### 2.1. Patient selection and data collection

This study utilized a dataset from the Korea Community Health Survey (KCHS) conducted by the Korea Disease Control and Prevention Agency (KDCA) from 2009 to 2022.^[11]^ The KCHS contains information on age, sex, sleep duration, smoking status, alcohol consumption frequency, and body mass index (BMI), which are known to influence hypertension and diabetes. KCHS employs a stratified 2-stage cluster sampling design to ensure representativeness of the adult population in Korea. Primary sampling units (PSU) are defined based on administrative districts (eup, myeon, and dong), and stratification is conducted within each city, county, and district according to population size as well as age and sex distribution. In the first stage, PSUs are selected using probability proportional to size sampling, and in the second stage, households within the selected PSUs are systematically sampled to construct the survey population. All individuals aged 19 years or older within the sampled households were included in the survey, yielding annual samples of 427,748 individuals in 2009 to 2010, 647,463 in 2011 to 2013, 677,603 in 2014 to 2016, 601,671 in 2017 to 2019, 182,538 in 2020, 180,628 in 2021, and 186,236 in 2022, totaling 2,903,887 individuals. To produce nationally and regionally representative estimates, sampling weights reflecting age, sex, and regional population structures are applied. Since its inception in 2008, the KCHS has maintained a consistent sampling framework and design across survey years, thereby ensuring comparability over time.^[12]^

The KCHS data for the years covered by this study were anonymized, and written informed consent was obtained from all participants before participating. The study protocol was approved by the Institutional Review Board of the KDCA (2010-02CON-22-P, 2011-05CON04-C, 2012-07CON-01-2C, 2013-06EXP-01-3C, 2014-08EXP-09-4CA, and 2016-10-01-TA) for primary data collection and Kyung Hee University for secondary analysis. Ethical considerations were upheld, adhering to the Declaration of Helsinki.

### 2.2. Ascertainment of sleep duration in patients with hypertension, diabetes, and those with both conditions

Our study aimed to analyze trends in sleep duration and risk factors among individuals with hypertension, diabetes, and those with both conditions, as well as to examine the impact of the COVID-19 pandemic on these trends. The definitions of the 4 terms used to achieve the objectives of this study – sleep duration, patients with hypertension, patients with diabetes, and patients with both hypertension and diabetes – are as follows. Sleep duration was defined by the response to the question, “How many hours do you usually sleep per day?” For this study, the responses were categorized into 4 groups: <6 hours, 6 to 7 hours, 7 to 8 hours, and >8 hours. Patients with hypertension were defined as those who answered “Yes” to the question, “Have you ever been diagnosed with hypertension by a doctor?” Similarly, patients with diabetes were defined as those who answered “Yes” to the question, “Have you ever been diagnosed with diabetes by a doctor?” Individuals with both hypertension and diabetes were defined as those who answered “Yes” to both of the above questions. Additionally, considering that the first case of SARS-CoV-2 infection in Korea was identified in January 2020, and the World Health Organization declared a pandemic in March 2020, the period from 2009 to 2019 is defined as pre-COVID-19 pandemic, and the period from 2020 to 2022 is defined as during the COVID-19 pandemic.^[12]^

### 2.3. Covariates

This study included 12 covariates for analysis: sex, age, region of residence, BMI group, sleep duration, subjective health level, depression counseling, stress counseling, education level, household income, smoking status, and alcohol consumption. As identified in previous studies, these covariates were selected based on their potential relevance. For sleep duration, we categorized it into 4 groups for analysis, referring to previous studies: <6 hours, 6 to 7 hours, 7 to 8 hours, and >8 hours.^[13]^ Additionally, consistent with previous studies, we referred to sleep durations of <6 hours per day as very short sleep duration and durations of >8 hours per day as long sleep duration.^[14,15]^

Additionally, the self-rate health of participants was assessed using the question, “How would you rate your overall health?” with possible responses spanning from “very good” to “very poor.” These responses were subsequently classified into 3 categories: “high” (encompassing “very good” and “good”), “middle,” and “low” (encompassing “poor” and “very poor”).

### 2.4. Statistical analysis

This study presented the outcomes using quantitative data, expressed as weighted proportions with 95% confidence intervals (CIs) or crude numbers with percentages, to identify trends in the prevalence of chronic diseases stratified by sleep duration groups. Data from the KCHS, spanning 2009 to 2022, were analyzed and categorized by year to assess the prevalence of hypertension, diabetes, and those with both conditions across sleep duration groups. To account for the complex sampling design of the KCHS, we used survey-weighted logistic regression models. Sampling weights were applied to obtain population-representative estimates, and stratification and clustering variables were incorporated to ensure appropriate variance estimation. β-Coefficients were estimated using log-linear regression models, with weighted prevalence (%) as the dependent variable and survey year as a continuous predictor. Given that the raw β estimates were small in magnitude, they were multiplied by 100 to facilitate interpretation and are presented as absolute percentage-point (%p) changes in prevalence with 95% CIs. Weighted odds ratios (wOR) and risk factors, along with their 95% CIs, were calculated using weighted logistic regression to improve estimation precision. All regression models were adjusted for potential confounders, excluding sleep duration, which was the primary variable of interest. Weighted logistic regression analysis was performed to examine the prevalence of hypertension, diabetes, and the co-occurrence of both conditions for each variable, while evaluating the risk factors in relation to sleep duration.^[16]^ The ratio of wOR was calculated to measure the change in the prevalence of sleep duration among individuals with hypertension and diabetes and those with both conditions before and during the COVID-19 pandemic. Analyses were performed to ensure accurate estimation, considering sex, age, region of residence, BMI group, sleep duration, subjective health level, depression counseling, stress counseling, education level, household income, smoking status, and alcohol consumption in all regression models. The ratio of wOR was calculated to assess the influence of each risk factor on the diseases, thereby delineating groups with heightened vulnerability to hypertension and diabetes and the simultaneous presence of both conditions across various variables. In addition, to assess whether the association between sleep duration and the outcomes differed before and during the COVID-19 pandemic, an interaction term between sleep duration and the COVID period was included in the logistic regression model. All statistical analyses were performed using SAS software (version 9.4; SAS Institute, Cary). A 2-sided *P*-value of <.05 was considered statistically significant.

## 3. Results

A total of 3,205,814 participants were surveyed in the KCHS database between 2009 and 2022. However, 302,417 participants were excluded because of missing information regarding education level, household income, or weight. Therefore, this study utilized the KCHS database, which included 2,903,887 participants from 2009 to 2022 (Fig. S1, Supplemental Digital Content, https://links.lww.com/MD/Q692). This database comprised 1,306,436 (44.99%) males and 1,597,451 (55.01%) females. Based on average sleep duration, the number of people with a sleep duration of <6 hours per day was 491,183 (16.91%), for 6 to 7 hours was 822,197 (28.31%), for 7 to 8 hours was 914,450 (31.49%), and for 8 hours or more was 676,057 (23.28%; Table 1).

**Table 1:** Baseline characteristics of Korean adults based on the data from KCHS, 2009 to 2022 (n = 2,903,887).

Variables	Total	Pre-pandemic				During the pandemic		
		2009–2010	2011–2013	2014–2016	2017–2019	2020	2021	2022
Overall, n	2,903,887	427,748 (14.73)	647,463 (22.30)	677,603 (23.33)	601,671 (20.72)	182,538 (6.29)	180,628 (6.22)	186,236 (6.41)
Sex, weighted % (95% CI)								
Male	45.26 (45.21–45.31)	46.18 (46.05–46.30)	45.09 (44.99–45.19)	45.40 (45.30–45.50)	44.77 (44.66–44.88)	44.93 (44.74–45.12)	45.12 (44.93–45.31)	45.34 (45.15–45.52)
Female	54.74 (54.69–54.79)	53.82 (53.70–53.95)	54.91 (54.81–55.01)	54.60 (54.50–54.70)	55.23 (55.12–55.34)	55.07 (54.88–55.26)	54.88 (54.69–55.07)	54.66 (54.48–54.85)
Age, yr, weighted % (95% CI)								
19–30	15.59 (15.53–15.66)	17.53 (17.36–17.71)	15.85 (15.71–15.98)	15.65 (15.52–15.79)	14.76 (14.62–14.90)	15.67 (15.43–15.90)	14.71 (14.49–14.93)	13.86 (13.65–14.08)
31–40	17.63 (17.55–17.71)	21.52 (21.29–21.75)	20.08 (19.90–20.26)	18.24 (18.08–18.41)	15.68 (15.51–15.85)	13.80 (13.55–14.05)	13.93 (13.68–14.19)	13.15 (12.91–13.39)
41–50	20.08 (20.01–20.16)	21.88 (21.68–22.08)	21.83 (21.67–21.99)	21.07 (20.91–21.23)	18.93 (18.77–19.10)	17.46 (17.20–17.73)	17.29 (17.02–17.56)	16.47 (16.21–16.72)
51–60	18.90 (18.83–18.97)	16.78 (16.62–16.95)	18.58 (18.44–18.72)	19.50 (19.36–19.64)	19.62 (19.47–19.76)	19.61 (19.37–19.86)	19.44 (19.20–19.68)	19.06 (18.82–19.30)
61–70	14.65 (14.59–14.72)	12.71 (12.55–12.86)	12.90 (12.78–13.03)	13.28 (13.15–13.41)	15.85 (15.71–15.99)	17.04 (16.80–17.29)	17.67 (17.43–17.92)	19.66 (19.40–19.91)
>70	13.14 (13.08–13.21)	9.58 (9.44–9.71)	10.77 (10.65–10.88)	12.25 (12.13–12.38)	15.16 (15.01–15.31)	16.42 (16.18–16.66)	16.95 (16.70–17.20)	17.80 (17.56–18.05)
Region of residence, weighted % (95% CI)								
Urban	81.81 (81.64–81.97)	79.84 (79.35–80.33)	81.49 (81.21–81.78)	82.22 (81.93–82.51)	82.19 (81.90–82.48)	82.56 (82.36–82.77)	82.77 (82.57–82.98)	82.78 (82.60–82.96)
Rural	18.19 (18.03–18.36)	20.16 (19.67–20.65)	18.51 (18.22–18.79)	17.78 (17.49–18.07)	17.81 (17.52–18.10)	17.44 (17.23–17.64)	17.23 (17.02–17.43)	17.22 (17.04–17.40)
BMI group,* weighted % (95% CI)								
Underweight	6.61 (6.57–6.65)	7.11 (7.00–7.22)	7.68 (7.59–7.77)	6.82 (6.74–6.90)	6.84 (6.75–6.93)	4.13 (4.01–4.24)	4.97 (4.84–5.09)	4.90 (4.78–5.02)
Normal weight	43.34 (43.26–43.42)	47.06 (46.85–47.26)	45.56 (45.40–45.73)	44.16 (44.00–44.32)	39.94 (39.77–40.11)	40.95 (40.67–41.23)	40.98 (40.70–41.26)	40.94 (40.67–41.22)
Overweight	23.46 (23.40–23.53)	23.44 (23.27–23.60)	23.17 (23.04–23.31)	23.33 (23.20–23.46)	23.31 (23.17–23.45)	24.11 (23.87–24.35)	24.18 (23.93–24.43)	23.98 (23.75–24.22)
Obese	26.59 (26.52–26.66)	22.40 (22.23–22.57)	23.58 (23.44–23.72)	25.69 (25.55–25.83)	29.91 (29.75–30.07)	30.81 (30.54–31.08)	29.87 (29.60–30.13)	30.18 (29.91–30.44)
Average sleep duration, hours/day, weighted % (95% CI)								
<6	16.59 (16.53–16.66)	14.38 (14.23–14.53)	16.06 (15.94–16.19)	17.49 (17.37–17.62)	17.19 (17.06–17.33)	15.36 (15.16–15.57)	17.50 (17.28–17.73)	18.40 (18.18–18.63)
6–7	30.09 (30.02–30.16)	29.02 (28.83–29.22)	30.67 (30.52–30.82)	31.48 (31.33–31.63)	29.97 (29.81–30.13)	27.83 (27.58–28.09)	29.19 (28.92–29.46)	29.45 (29.19–29.71)
7–8	32.05 (31.98–32.13)	32.23 (32.02–32.43)	32.04 (31.89–32.20)	31.54 (31.38–31.69)	32.16 (32.00–32.32)	33.57 (33.29–33.85)	32.05 (31.77–32.33)	31.65 (31.38–31.92)
≥8	21.26 (21.19–21.33)	24.37 (24.18–24.55)	21.22 (21.08–21.36)	19.49 (19.36–19.62)	20.68 (20.53–20.82)	23.23 (22.98–23.48)	21.25 (21.00–21.50)	20.50 (20.27–20.72)
Subjective health-level, weighted % (95% CI)								
High	41.57 (41.48–41.66)	44.28 (44.04–44.53)	41.60 (41.42–41.79)	39.98 (39.80–40.16)	37.68 (37.48–37.87)	50.42 (50.11–50.74)	43.07 (42.77–43.38)	42.99 (42.68–43.29)
Middle	42.40 (42.32–42.49)	39.31 (39.08–39.54)	41.75 (41.57–41.93)	43.67 (43.50–43.84)	45.26 (45.08–45.44)	38.41 (38.12–38.71)	42.76 (42.47–43.05)	41.50 (41.22–41.79)
Low	16.03 (15.96–16.09)	16.41 (16.24–16.57)	16.65 (16.51–16.78)	16.34 (16.21–16.47)	17.06 (16.93–17.20)	11.16 (10.97–11.36)	14.17 (13.96–14.37)	15.51 (15.29–15.72)
Hypertension, weighted % (95% CI)								
Yes	21.73 (21.65–21.80)	17.81 (17.65–17.98)	19.59 (19.45–19.73)	21.10 (20.96–21.24)	23.78 (23.62–23.94)	24.29 (24.03–24.55)	24.99 (24.73–25.25)	26.64 (26.38–26.91)
No	78.27 (78.20–78.35)	82.19 (82.02–82.35)	80.41 (80.27–80.55)	78.90 (78.76–79.04)	76.22 (76.06–76.38)	75.71 (75.45–75.97)	75.01 (74.75–75.27)	73.36 (73.09–73.62)
Diabetes, weighted % (95% CI)								
Yes	8.81 (8.76–8.85)	6.70 (6.60–6.80)	7.46 (7.37–7.55)	8.46 (8.37–8.55)	9.73 (9.63–9.83)	10.45 (10.28–10.63)	11.04 (10.86–11.22)	11.95 (11.76–12.13)
No	91.19 (91.15–91.24)	93.30 (93.20–93.40)	92.54 (92.45–92.63)	91.54 (91.46–91.63)	90.27 (90.17–90.37)	89.55 (89.37–89.72)	88.96 (88.78–89.14)	88.05 (87.87–88.24)
Depression counseling, weighted % (95% CI)								
Yes	1.24 (1.23–1.26)	0.99 (0.95–1.03)	0.96 (0.93–0.99)	1.16 (1.13–1.19)	1.32 (1.28–1.36)	1.52 (1.44–1.59)	1.79 (1.72–1.87)	1.91 (1.83–1.98)
No	98.76 (98.74–98.77)	99.01 (98.97–99.05)	99.04 (99.01–99.07)	98.84 (98.81–98.87)	98.68 (98.64–98.72)	98.48 (98.41–98.56)	98.21 (98.13–98.28)	98.09 (98.02–98.17)
Stress counseling, weighted % (95% CI)								
Yes	2.07 (2.05–2.09)	1.58 (1.53–1.63)	1.70 (1.66–1.74)	2.02 (1.97–2.06)	2.13 (2.08–2.18)	2.58 (2.48–2.67)	2.87 (2.78–2.97)	2.94 (2.84–3.04)
No	97.93 (97.91–97.95)	98.42 (98.37–98.47)	98.30 (98.26–98.34)	97.98 (97.94–98.03)	97.87 (97.82–97.92)	97.42 (97.33–97.52)	97.13 (97.03–97.22)	97.06 (96.96–97.16)
Level of education, weighted % (95% CI)								
Elementary school or lower education	15.87 (15.79–15.94)	18.48 (18.27–18.69)	16.83 (16.68–16.97)	15.21 (15.07–15.35)	15.30 (15.16–15.45)	14.97 (14.76–15.19)	14.02 (13.81–14.22)	13.94 (13.73–14.14)
Middle school	9.87 (9.82–9.92)	10.57 (10.43–10.70)	10.05 (9.95–10.16)	9.44 (9.34–9.54)	9.82 (9.71–9.93)	9.82 (9.64–10.00)	9.38 (9.20–9.55)	9.90 (9.73–10.07)
High school	30.45 (30.37–30.53)	31.54 (31.32–31.75)	30.73 (30.56–30.90)	29.98 (29.82–30.15)	30.02 (29.84–30.20)	30.43 (30.14–30.71)	30.45 (30.16–30.75)	30.13 (29.85–30.41)
College or higher education	43.81 (43.70–43.93)	39.42 (39.12–39.72)	42.39 (42.16–42.61)	45.36 (45.14–45.59)	44.86 (44.62–45.11)	44.78 (44.43–45.13)	46.15 (45.79–46.52)	46.03 (45.68–46.38)
Household income, weighted % (95% CI)								
Lowest quartile	26.49 (26.34–26.63)	17.52 (17.20–17.84)	24.28 (24.01–24.56)	21.68 (21.41–21.94)	31.17 (30.87–31.48)	33.38 (32.90–33.86)	35.43 (34.95–35.90)	38.40 (37.93–38.87)
Second quartile	29.53 (29.41–29.64)	29.50 (29.19–29.81)	30.49 (30.25–30.73)	31.69 (31.44–31.94)	29.50 (29.25–29.74)	27.07 (26.67–27.48)	25.97 (25.59–26.35)	25.52 (25.15–25.90)
Third quartile	31.97 (31.85–32.09)	39.79 (39.45–40.13)	33.25 (33.00–33.50)	32.96 (32.72–33.21)	28.42 (28.18–28.66)	28.50 (28.12–28.88)	27.88 (27.51–28.25)	26.30 (25.95–26.65)
Highest quartile	12.02 (11.94–12.09)	13.18 (12.98–13.39)	11.97 (11.83–12.12)	13.68 (13.52–13.83)	10.91 (10.77–11.06)	11.05 (10.82–11.27)	10.72 (10.50–10.95)	9.78 (9.59–9.98)
Smoking status, weighted % (95% CI)								
Smoker	19.36 (19.30–19.42)	23.14 (22.97–23.30)	21.19 (21.06–21.32)	19.46 (19.33–19.59)	17.68 (17.55–17.81)	16.44 (16.23–16.65)	16.17 (15.95–16.38)	16.46 (16.25–16.67)
Ex-smoker	16.94 (16.88–16.99)	13.92 (13.78–14.05)	15.84 (15.73–15.95)	16.94 (16.83–17.05)	18.00 (17.88–18.11)	17.84 (17.64–18.04)	18.34 (18.13–18.55)	21.21 (21.00–21.42)
Nonsmoker	63.70 (63.64–63.77)	62.95 (62.78–63.11)	62.98 (62.85–63.11)	63.60 (63.47–63.73)	64.33 (64.19–64.47)	65.72 (65.49–65.96)	65.50 (65.25–65.74)	62.33 (62.09–62.56)
Alcohol consumption, weighted % (95% CI)								
Nondrinker and 1 d/mo	56.55 (56.47–56.64)	57.00 (56.78–57.22)	54.82 (54.64–54.99)	53.33 (53.15–53.50)	55.58 (55.39–55.77)	62.53 (62.23–62.84)	64.12 (63.82–64.42)	61.33 (61.04–61.63)
2–9 d/mo	37.07 (36.98–37.15)	36.69 (36.48–36.91)	38.47 (38.29–38.64)	39.71 (39.53–39.88)	37.62 (37.44–37.80)	32.57 (32.27–32.86)	31.11 (30.82–31.40)	33.40 (33.11–33.69)
Over 10 d/mo	6.38 (6.34–6.42)	6.31 (6.21–6.40)	6.72 (6.64–6.80)	6.97 (6.89–7.05)	6.80 (6.72–6.88)	4.90 (4.78–5.02)	4.77 (4.65–4.89)	5.27 (5.14–5.39)

Weights were applied to obtain population-representative estimates, accounting for stratification and clustering variables.

BMI = body mass index, CI = confidence interval, KCHS = Korea Community Health Survey.

* According to the Asian-Pacific guidelines, BMI is divided into 4 groups: underweight (<18.5 kg/m^2^), normal (18.5–22.9 kg/m^2^), overweight (23.0–24.9 kg/m^2^), and obese (≥25.0 kg/m^2^).

**Figure 1.**
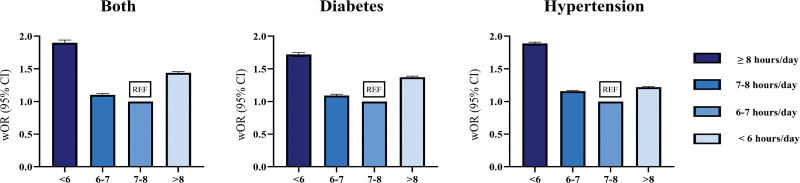
Overall weighted odds ratios of sleep duration prevalence in individuals with hypertension, diabetes, and those both conditions (wOR [95% CI]). CI = confidential interval, wOR = weighted odds ratio.

Individuals who have both hypertension and diabetes increased before (β, 0.87 [95% CI, 0.83–0.90]) and during the COVID-19 pandemic (β, 0.64 [95% CI, 0.58–0.70]). This trend was particularly pronounced among individuals who slept <6 hours per day both before (β, 1.28 [95% CI, 1.16–1.40]) and during the COVID-19 pandemic (β, 1.21 [95% CI, 1.03–1.39]). However, the rate of increase declined during the COVID-19 pandemic compared to the period before the pandemic (<6: differences in β-coefficients [β_diff_], −0.07 [−0.29 to 0.14]; 6–7: −0.40 [−0.56 to −0.24]; 7–8: −0.40 [−0.56 to −0.24]; ≥8: −0.47 [−0.62 to −0.31]). The weighted percentage of individuals with both hypertension and diabetes was highest among those who slept <6 hours, followed by those who slept 8 hours or more. Additionally, the weighted percentage was higher in females than in males (Fig. 1 and Table 2). This trend was similarly observed among individuals with either hypertension or diabetes alone (Tables S1–S4, Supplemental Digital Content, https://links.lww.com/MD/Q692).

**Table 2:** National trends in sleep duration among individuals with both hypertension and diabetes, along with β-coefficients before and during the COVID-19 pandemic (weighted % [95% CI]).

Variables	Sleep duration (hours/day)	Pre-pandemic				During the pandemic			Trends in the pre-pandemic era, β (95% CI)	Trends in the pandemic era, β (95% CI)	β_diff_ between 2009–2019 and 2019–2022 (95% CI)
		2009–2010	2011–2013	2014–2016	2017–2019	2020	2021	2022			
Overall	<6	6.98 (6.69–7.27)	8.18 (7.94–8.43)	9.48 (9.24–9.73)	11.42 (11.13–11.71)	13.31 (12.74–13.88)	13.86 (13.32–14.39)	15.19 (14.64–15.73)	**1.28 (1.16–1.40**)	**1.21 (1.03–1.39**)	−0.07 (−0.29 to 0.14)
	6–7	3.96 (3.80–4.11)	4.44 (4.31–4.57)	5.22 (5.08–5.35)	6.52 (6.36–6.68)	7.72 (7.40–8.04)	7.71 (7.41–8.02)	8.45 (8.14–8.76)	**0.73 (0.66–0.79**)	**0.62 (0.52–0.72**)	**−0.40 (−0.56 to −0.24**)
	7–8	3.41 (3.28–3.54)	4.11 (3.99–4.23)	4.93 (4.80–5.06)	5.93 (5.78–6.07)	5.98 (5.73–6.23)	6.48 (6.22–6.74)	7.56 (7.27–7.86)	**0.71 (0.65–0.76**)	**0.45 (0.36–0.54**)	**−0.40 (−0.56 to −0.24**)
	≥8	5.26 (5.07–5.45)	6.18 (6.00–6.36)	7.27 (7.08–7.47)	8.85 (8.62–9.07)	8.72 (8.37–9.08)	9.19 (8.82–9.57)	10.76 (10.34–11.18)	**0.98 (0.90–1.06**)	**0.51 (0.38–0.64**)	**−0.47 (−0.62 to −0.31**)
Sex											
Male	<6	5.70 (5.31–6.10)	6.99 (6.64–7.34)	8.67 (8.30–9.04)	10.83 (10.38–11.28)	13.04 (12.16–13.91)	13.72 (12.89–14.55)	14.89 (14.05–15.72)	**1.50 (1.33–1.67**)	**1.38 (1.10–1.66**)	−0.12 (−0.45 to 0.20)
	6–7	3.84 (3.63–4.05)	4.47 (4.29–4.66)	5.34 (5.15–5.54)	6.77 (6.53–7.01)	8.10 (7.62–8.57)	8.15 (7.69–8.60)	9.46 (8.97–9.95)	**0.81 (0.72–0.90**)	**0.84 (0.69–0.99**)	**−0.69 (−0.94 to −0.44**)
	7–8	3.72 (3.52–3.92)	4.68 (4.49–4.86)	5.91 (5.70–6.12)	6.80 (6.57–7.03)	6.62 (6.24–7.01)	7.71 (7.29–8.13)	8.68 (8.24–9.13)	**0.87 (0.78–0.95**)	**0.55 (0.41–0.68**)	**−0.69 (−0.94 to −0.44**)
	≥8	6.29 (5.97–6.60)	7.53 (7.23–7.83)	9.37 (9.03–9.71)	11.16 (10.78–11.53)	10.62 (10.05–11.19)	12.00 (11.36–12.64)	13.09 (12.41–13.76)	**1.34 (1.21–1.48**)	**0.63 (0.42–0.84**)	**−0.71 (−0.96 to −0.46**)
Female	<6	8.02 (7.61–8.43)	9.06 (8.73–9.39)	10.08 (9.75–10.40)	11.81 (11.44–12.18)	13.48 (12.77–14.20)	13.95 (13.27–14.63)	15.38 (14.70–16.07)	**1.04 (0.88–1.21**)	**1.07 (0.84–1.30**)	0.03 (−0.25 to 0.30)
	6–7	4.08 (3.86–4.29)	4.41 (4.23–4.58)	5.10 (4.92–5.28)	6.30 (6.08–6.51)	7.40 (6.98–7.82)	7.33 (6.93–7.74)	7.57 (7.17–7.96)	**0.63 (0.55–0.72**)	**0.41 (0.28–0.54**)	−0.17 (−0.36 to 0.02)
	7–8	3.14 (2.97–3.30)	3.65 (3.50–3.80)	4.13 (3.97–4.28)	5.21 (5.03–5.39)	5.43 (5.12–5.75)	5.44 (5.12–5.76)	6.63 (6.26–6.99)	**0.55 (0.48–0.62**)	**0.37 (0.26–0.48**)	−0.17 (−0.36 to 0.02)
	≥8	4.51 (4.29–4.74)	5.27 (5.06–5.48)	5.82 (5.60–6.04)	7.21 (6.96–7.47)	7.28 (6.86–7.71)	7.08 (6.65–7.50)	8.91 (8.41–9.41)	**0.67 (0.58–0.77**)	**0.38 (0.23–0.52**)	**−0.30 (−0.48 to −0.12**)
Age, yr											
19–30	<6	0.03 (0.00–0.06)	0.05 (0.00–0.10)	0.09 (0.02–0.16)	0.12 (0.04–0.20)	N/A	0.15 (0.00–0.35)	0.37 (0.05–0.69)	**0.04 (0.01–0.07**)	0.06 (−0.03 to 0.16)	0.03 (−0.07 to 0.13)
	6–7	0.06 (0.02–0.11)	0.08 (0.03–0.13)	0.05 (0.02–0.08)	0.04 (0.01–0.07)	0.15 (0.05–0.25)	0.08 (0.01–0.15)	0.19 (0.05–0.32)	−0.01 (−0.03 to 0.02)	0.04 (0.00–0.08)	0.01 (−0.07 to 0.10)
	7–8	0.03 (0.00–0.06)	0.03 (0.01–0.06)	0.07 (0.04–0.11)	0.09 (0.04–0.14)	0.07 (0.02–0.12)	0.02 (0.00–0.05)	0.11 (0.04–0.18)	**0.03 (0.01–0.04**)	−0.01 (−0.03 to 0.02)	0.01 (−0.07 to 0.10)
	≥8	0.04 (0.01–0.08)	0.08 (0.04–0.13)	0.13 (0.06–0.19)	0.05 (0.02–0.08)	0.10 (0.00–0.19)	0.12 (0.02–0.22)	0.16 (0.04–0.29)	0.00 (−0.01 to 0.02)	**0.04 (0.01–0.07**)	0.03 (0.00–0.07)
31–40	<6	0.39 (0.23–0.55)	0.42 (0.28–0.57)	0.42 (0.28–0.55)	0.73 (0.52–0.95)	1.15 (0.65–1.66)	1.44 (0.92–1.96)	0.93 (0.48–1.38)	**0.12 (0.02–0.21**)	0.13 (−0.04 to 0.31)	0.02 (−0.18 to 0.21)
	6–7	0.18 (0.12–0.24)	0.30 (0.22–0.38)	0.40 (0.32–0.48)	0.45 (0.34–0.56)	0.64 (0.41–0.88)	0.57 (0.34–0.79)	0.69 (0.43–0.96)	**0.09 (0.05–0.13**)	0.08 (−0.01 to 0.17)	0.14 (−0.06 to 0.33)
	7–8	0.22 (0.15–0.30)	0.25 (0.19–0.31)	0.38 (0.30–0.46)	0.33 (0.25–0.41)	0.42 (0.26–0.58)	0.70 (0.47–0.92)	0.63 (0.43–0.83)	**0.05 (0.02–0.09**)	**0.12 (0.05–0.19**)	0.14 (−0.06 to 0.33)
	≥8	0.32 (0.22–0.42)	0.31 (0.22–0.40)	0.29 (0.21–0.38)	0.44 (0.32–0.56)	0.55 (0.33–0.77)	0.44 (0.23–0.65)	0.75 (0.45–1.05)	0.03 (−0.02 to 0.08)	0.07 (−0.02 to 0.17)	0.04 (−0.07 to 0.15)
41–50	<6	1.83 (1.50–2.16)	2.33 (2.03–2.64)	2.32 (2.04–2.60)	2.89 (2.52–3.26)	3.15 (2.44–3.85)	2.83 (2.19–3.48)	3.87 (3.11–4.62)	**0.30 (0.13–0.46**)	**0.27 (0.01–0.53**)	−0.03 (−0.34 to 0.28)
	6–7	1.48 (1.30–1.67)	1.42 (1.28–1.57)	1.62 (1.47–1.77)	1.98 (1.78–2.17)	1.72 (1.39–2.05)	2.47 (2.08–2.87)	2.72 (2.30–3.14)	**0.20 (0.11–0.29**)	**0.25 (0.10–0.40**)	0.19 (−0.13 to 0.51)
	7–8	1.27 (1.11–1.43)	1.38 (1.24–1.52)	1.46 (1.32–1.60)	1.70 (1.53–1.87)	1.93 (1.61–2.26)	1.88 (1.55–2.22)	2.42 (2.03–2.81)	**0.16 (0.08–0.24**)	**0.20 (0.06–0.33**)	0.19 (−0.13 to 0.51)
	≥8	1.92 (1.64–2.19)	1.80 (1.58–2.01)	1.82 (1.58–2.06)	2.02 (1.75–2.29)	2.50 (2.01–3.00)	2.61 (2.07–3.15)	2.53 (1.98–3.08)	0.02 (−0.12 to 0.16)	0.14 (−0.05 to 0.33)	0.12 (−0.12 to 0.35)
51–60	<6	7.01 (6.34–7.68)	7.17 (6.65–7.69)	7.70 (7.20–8.19)	8.23 (7.69–8.78)	8.79 (7.72–9.86)	9.75 (8.73–10.77)	9.16 (8.18–10.13)	**0.54 (0.26–0.83**)	**0.38 (0.01–0.75**)	−0.17 (−0.64 to 0.30)
	6–7	6.15 (5.67–6.63)	5.80 (5.47–6.14)	5.97 (5.65–6.29)	6.67 (6.31–7.04)	6.85 (6.22–7.49)	7.42 (6.78–8.06)	7.32 (6.69–7.96)	**0.27 (0.07–0.46**)	**0.28 (0.03–0.53**)	0.08 (−0.38 to 0.55)
	7–8	5.99 (5.55–6.44)	5.98 (5.62–6.34)	6.21 (5.87–6.55)	6.15 (5.80–6.50)	6.02 (5.44–6.60)	6.53 (5.88–7.18)	7.25 (6.60–7.91)	0.16 (−0.03 to 0.34)	**0.32 (0.07–0.57**)	0.08 (−0.38 to 0.55)
	≥8	7.80 (7.20–8.39)	8.03 (7.53–8.54)	8.77 (8.22–9.32)	8.05 (7.51–8.59)	9.24 (8.27–10.21)	8.30 (7.38–9.21)	9.14 (8.12–10.17)	0.18 (−0.10 to 0.46)	0.32 (−0.06 to 0.71)	0.14 (−0.33 to 0.61)
61–70	<6	19.26 (18.07–20.45)	19.72 (18.79–20.64)	20.99 (20.09–21.88)	20.36 (19.49–21.22)	21.21 (19.66–22.75)	19.59 (18.20–20.97)	21.54 (20.23–22.86)	0.43 (−0.04 to 0.90)	0.19 (−0.33 to 0.72)	−0.23 (−0.94 to 0.47)
	6–7	15.83 (14.91–16.75)	18.18 (17.37–18.98)	18.44 (17.68–19.20)	17.62 (16.91–18.33)	17.75 (16.55–18.96)	16.49 (15.39–17.59)	17.15 (16.10–18.20)	0.36 (−0.02 to 0.74)	−0.25 (−0.67 to 0.17)	−0.52 (−1.09 to 0.06)
	7–8	16.02 (15.10–16.93)	18.00 (17.22–18.78)	18.99 (18.23–19.76)	17.86 (17.16–18.55)	17.16 (16.01–18.31)	17.56 (16.43–18.68)	18.70 (17.57–19.83)	**0.50 (0.12–0.87**)	0.17 (−0.27 to 0.61)	−0.52 (−1.09 to 0.06)
	≥8	19.43 (18.41–20.46)	21.38 (20.44–22.32)	22.40 (21.43–23.37)	22.48 (21.54–23.41)	20.55 (19.13–21.97)	21.87 (20.38–23.35)	23.29 (21.85–24.73)	**1.12 (0.66–1.59**)	0.08 (−0.50 to 0.66)	**−1.04 (−1.78 to −0.29**)
>70	<6	21.44 (20.13–22.76)	27.86 (26.77–28.95)	33.05 (32.02–34.08)	34.54 (33.51–35.58)	35.38 (33.63–37.12)	36.76 (35.17–38.36)	38.07 (36.53–39.61)	**4.09 (3.54–4.63**)	**1.07 (0.46–1.69**)	**−3.01 (−3.83 to −2.20**)
	6–7	22.26 (20.91–23.61)	24.81 (23.74–25.87)	30.94 (29.87–32.01)	32.50 (31.44–33.56)	31.39 (29.70–33.08)	31.57 (29.90–33.24)	33.68 (32.08–35.29)	**3.59 (3.04–4.13**)	0.27 (−0.37 to 0.90)	**−1.40 (−1.93 to −0.86**)
	7–8	21.64 (20.27–23.00)	25.97 (24.85–27.09)	29.66 (28.58–30.74)	32.47 (31.42–33.51)	29.57 (27.97–31.17)	32.90 (31.23–34.58)	35.89 (34.14–37.64)	**3.51 (2.96–4.07**)	**1.01 (0.35–1.67**)	**−1.40 (−1.93 to −0.86**)
	≥8	22.90 (21.73–24.07)	26.72 (25.73–27.72)	30.95 (29.93–31.96)	34.86 (33.88–35.85)	33.32 (31.75–34.89)	34.63 (33.00–36.27)	36.79 (35.16–38.42)	**4.08 (3.59–4.56**)	0.49 (−0.12 to 1.11)	**−3.58 (−4.37 to −2.80**)
Region of residence											
Urban	<6	6.61 (6.28–6.95)	7.55 (7.28–7.83)	8.75 (8.48–9.03)	10.67 (10.35–11.00)	12.64 (11.99–13.28)	13.20 (12.60–13.80)	14.45 (13.83–15.06)	**1.19 (1.05–1.32**)	**1.17 (0.97–1.38**)	−0.01 (−0.26 to 0.23)
	6–7	3.63 (3.46–3.80)	4.00 (3.86–4.14)	4.69 (4.55–4.84)	5.93 (5.75–6.11)	7.09 (6.73–7.44)	7.18 (6.84–7.52)	7.91 (7.57–8.26)	**0.65 (0.58–0.72**)	**0.62 (0.51–0.73**)	**−0.35 (−0.54 to −0.17**)
	7–8	3.11 (2.96–3.27)	3.67 (3.54–3.81)	4.46 (4.32–4.60)	5.33 (5.16–5.49)	5.41 (5.13–5.69)	5.84 (5.55–6.12)	7.06 (6.73–7.38)	**0.63 (0.56–0.69**)	**0.45 (0.35–0.55**)	**−0.35 (−0.54 to −0.17**)
	≥8	4.79 (4.57–5.02)	5.58 (5.37–5.79)	6.58 (6.35–6.81)	7.93 (7.67–8.18)	7.94 (7.54–8.34)	8.30 (7.87–8.73)	9.84 (9.36–10.31)	**0.86 (0.76–0.96**)	**0.48 (0.34–0.63**)	**−0.38 (−0.55 to −0.20**)
Rural	<6	8.60 (8.06–9.15)	11.33 (10.84–11.81)	13.22 (12.69–13.74)	15.19 (14.58–15.80)	16.66 (15.56–17.76)	17.01 (15.91–18.11)	18.76 (17.66–19.87)	**1.79 (1.56–2.03**)	**1.31 (0.95–1.68**)	**−0.48 (−0.91 to −0.05**)
	6–7	5.58 (5.24–5.93)	6.89 (6.58–7.19)	8.24 (7.92–8.56)	9.90 (9.50–10.30)	11.32 (10.59–12.04)	10.81 (10.08–11.55)	11.53 (10.81–12.24)	**1.19 (1.05–1.34**)	**0.64 (0.41–0.87**)	**−0.53 (−0.82 to −0.25**)
	7–8	4.69 (4.43–4.95)	6.24 (5.98–6.50)	7.35 (7.07–7.64)	9.04 (8.70–9.39)	9.09 (8.52–9.67)	10.12 (9.46–10.78)	10.37 (9.68–11.06)	**1.16 (1.05–1.28**)	**0.55 (0.35–0.76**)	**−0.53 (−0.82 to −0.25**)
	≥8	6.87 (6.54–7.20)	8.51 (8.17–8.84)	10.10 (9.73–10.47)	12.68 (12.22–13.14)	12.33 (11.54–13.13)	13.29 (12.49–14.08)	15.03 (14.15–15.92)	**1.52 (1.36–1.67**)	**0.79 (0.52–1.05**)	**−0.73 (−1.03 to −0.42**)
BMI group*											
Underweight	<6	5.73 (4.91–6.55)	8.88 (8.12–9.65)	9.92 (9.10–10.74)	11.45 (10.49–12.40)	6.20 (4.66–7.74)	10.33 (8.53–12.13)	9.65 (8.00–11.30)	**1.44 (1.07–1.81**)	−0.31 (−0.91 to 0.30)	**−1.75 (−2.46 to −1.04**)
	6–7	2.93 (2.46–3.40)	4.31 (3.89–4.74)	4.47 (4.04–4.90)	5.61 (5.06–6.16)	2.43 (1.51–3.35)	4.64 (3.67–5.62)	4.02 (3.19–4.86)	**0.59 (0.39–0.78**)	**−0.46 (−0.73 to −0.19**)	**−1.68 (−2.11 to −1.25**)
	7–8	1.99 (1.68–2.31)	3.62 (3.26–3.98)	4.04 (3.65–4.43)	4.70 (4.24–5.15)	2.04 (1.35–2.73)	2.72 (2.13–3.31)	2.84 (2.17–3.51)	**0.64 (0.48–0.80**)	**−0.57 (−0.79 to −0.36**)	**−1.68 (−2.11 to −1.25**)
	≥8	3.68 (3.22–4.13)	5.97 (5.49–6.45)	5.79 (5.30–6.29)	7.58 (6.98–8.17)	3.53 (2.63–4.43)	5.49 (4.53–6.44)	4.89 (3.90–5.88)	**0.89 (0.69–1.10**)	**−0.79 (−1.09 to −0.50**)	**−1.69 (−2.05 to −1.33**)
Normal weight	<6	4.73 (4.37–5.09)	5.31 (5.02–5.60)	6.31 (6.00–6.62)	7.12 (6.76–7.48)	9.41 (8.65–10.17)	9.82 (9.10–10.55)	10.38 (9.67–11.10)	**0.70 (0.56–0.85**)	**1.03 (0.81–1.26**)	**0.33 (0.06–0.60**)
	6–7	2.31 (2.15–2.48)	2.48 (2.34–2.63)	3.00 (2.85–3.15)	3.37 (3.19–3.55)	4.77 (4.39–5.15)	4.51 (4.15–4.88)	4.97 (4.60–5.34)	**0.31 (0.25–0.38**)	**0.50 (0.39–0.61**)	−0.12 (−0.32 to 0.08)
	7–8	1.86 (1.73–2.00)	2.28 (2.15–2.40)	2.68 (2.55–2.82)	3.10 (2.94–3.26)	3.44 (3.17–3.72)	3.70 (3.40–3.99)	4.50 (4.17–4.83)	**0.34 (0.28–0.40**)	**0.38 (0.28–0.48**)	−0.12 (−0.32 to 0.08)
	≥8	2.98 (2.77–3.18)	3.51 (3.32–3.70)	4.09 (3.88–4.31)	5.09 (4.84–5.35)	5.18 (4.77–5.58)	5.59 (5.16–6.02)	7.00 (6.51–7.49)	**0.55 (0.46–0.64**)	**0.48 (0.33–0.62**)	−0.08 (−0.25 to 0.09)
Overweight	<6	7.50 (6.88–8.12)	8.90 (8.36–9.45)	10.24 (9.71–10.77)	11.65 (11.04–12.26)	13.26 (12.14–14.38)	14.11 (13.02–15.20)	16.16 (15.06–17.25)	**1.18 (0.93–1.43**)	**1.41 (1.04–1.77**)	0.22 (−0.22 to 0.66)
	6–7	4.50 (4.17–4.83)	5.20 (4.92–5.48)	5.87 (5.58–6.16)	7.22 (6.87–7.58)	8.01 (7.36–8.65)	7.91 (7.30–8.51)	9.33 (8.65–10.01)	**0.73 (0.60–0.87**)	**0.61 (0.39–0.82**)	0.01 (−0.34 to 0.36)
	7–8	4.01 (3.72–4.30)	4.98 (4.71–5.25)	5.86 (5.57–6.15)	6.64 (6.31–6.96)	6.54 (6.00–7.07)	7.28 (6.72–7.84)	8.76 (8.11–9.40)	**0.75 (0.63–0.88**)	**0.51 (0.31–0.70**)	0.01 (−0.34 to 0.36)
	≥8	6.92 (6.45–7.39)	7.52 (7.09–7.95)	9.06 (8.60–9.53)	9.95 (9.46–10.45)	10.31 (9.50–11.11)	11.07 (10.18–11.96)	12.71 (11.78–13.65)	**0.88 (0.68–1.07**)	**0.84 (0.54–1.13**)	−0.04 (−0.39 to 0.31)
Obese	<6	11.39 (10.66–12.13)	12.50 (11.90–13.11)	13.90 (13.34–14.47)	16.96 (16.34–17.58)	19.27 (18.10–20.44)	19.87 (18.72–21.02)	22.13 (20.95–23.31)	**1.65 (1.37–1.93**)	**1.61 (1.22–1.99**)	−0.04 (−0.52 to 0.43)
	6–7	7.37 (6.93–7.81)	7.77 (7.42–8.12)	8.90 (8.55–9.24)	10.81 (10.42–11.19)	12.47 (11.75–13.19)	12.77 (12.04–13.49)	13.74 (13.02–14.46)	**1.00 (0.83–1.16**)	**0.97 (0.74–1.21**)	**−0.46 (−0.81 to −0.10**)
	7–8	7.39 (6.96–7.83)	7.85 (7.49–8.20)	9.29 (8.92–9.66)	10.49 (10.11–10.86)	10.40 (9.79–11.02)	11.37 (10.70–12.04)	12.59 (11.90–13.27)	**0.88 (0.72–1.04**)	**0.66 (0.44–0.88**)	**−0.46 (−0.81 to −0.10**)
	≥8	10.48 (9.90–11.06)	11.79 (11.25–12.33)	13.55 (12.99–14.11)	15.14 (14.58–15.70)	14.85 (13.98–15.72)	14.96 (14.05–15.86)	17.09 (16.10–18.08)	**1.29 (1.06–1.52**)	**0.49 (0.18–0.81**)	**−0.80 (−1.19 to −0.40**)
Level of education											
High school or lower education	<6	14.27 (13.62–14.93)	17.65 (17.07–18.23)	22.18 (21.56–22.81)	25.13 (24.44–25.82)	26.81 (25.54–28.09)	28.28 (27.04–29.51)	31.54 (30.32–32.77)	**3.68 (3.38–3.99**)	**1.98 (1.54–2.43**)	**−1.70 (−2.24 to −1.16**)
	6–7	11.33 (10.81–11.85)	14.08 (13.60–14.56)	17.94 (17.39–18.50)	21.98 (21.33–22.62)	22.24 (21.12–23.35)	23.32 (22.15–24.49)	26.23 (25.06–27.40)	**3.55 (3.29–3.81**)	**1.32 (0.89–1.75**)	**−1.34 (−1.74 to −0.94**)
	7–8	10.53 (10.06–11.00)	14.11 (13.63–14.59)	17.60 (17.05–18.14)	21.22 (20.61–21.84)	21.55 (20.46–22.65)	23.74 (22.56–24.92)	27.22 (25.94–28.50)	**3.51 (3.26–3.75**)	**1.83 (1.39–2.27**)	**−1.34 (−1.74 to −0.94**)
	≥8	13.82 (13.27–14.37)	17.49 (16.94–18.03)	21.64 (21.01–22.27)	25.99 (25.29–26.68)	26.62 (25.42–27.82)	28.23 (26.93–29.52)	30.37 (29.09–31.65)	**4.04 (3.76–4.33**)	**1.47 (1.01–1.94**)	**−2.57 (−3.12 to −2.03**)
College or higher education	<6	3.10 (2.84–3.35)	3.76 (3.54–3.97)	4.35 (4.14–4.56)	5.70 (5.44–5.96)	7.29 (6.74–7.84)	7.83 (7.32–8.35)	8.21 (7.70–8.72)	**0.78 (0.67–0.89**)	**0.85 (0.69–1.02**)	0.07 (−0.13 to 0.27)
	6–7	1.95 (1.82–2.08)	2.27 (2.17–2.38)	2.82 (2.70–2.93)	3.54 (3.40–3.68)	4.57 (4.28–4.85)	4.82 (4.55–5.09)	5.19 (4.91–5.47)	**0.48 (0.42–0.53**)	**0.55 (0.46–0.64**)	−0.14 (−0.29 to 0.02)
	7–8	1.72 (1.61–1.83)	2.07 (1.97–2.17)	2.71 (2.60–2.82)	3.30 (3.17–3.42)	3.43 (3.22–3.64)	4.03 (3.80–4.27)	4.67 (4.42–4.92)	**0.49 (0.44–0.54**)	**0.40 (0.32–0.47**)	−0.14 (−0.29 to 0.02)
	≥8	2.32 (2.16–2.47)	2.72 (2.58–2.86)	3.38 (3.22–3.54)	4.31 (4.12–4.49)	4.41 (4.12–4.71)	5.03 (4.70–5.35)	6.14 (5.76–6.52)	**0.57 (0.50–0.64**)	**0.50 (0.38–0.61**)	−0.07 (−0.20 to 0.06)
Household income											
Lowest and second quartile	<6	3.84 (3.49–4.19)	4.34 (4.07–4.61)	4.49 (4.23–4.75)	5.85 (5.56–6.15)	7.31 (6.69–7.94)	7.92 (7.33–8.50)	8.79 (8.20–9.37)	**0.61 (0.48–0.75**)	**0.87 (0.67–1.06**)	**0.25 (0.02–0.49**)
	6–7	2.27 (2.10–2.44)	2.46 (2.33–2.59)	2.80 (2.66–2.93)	3.73 (3.57–3.89)	4.11 (3.81–4.41)	4.87 (4.55–5.18)	5.23 (4.93–5.54)	**0.44 (0.37–0.51**)	**0.49 (0.39–0.59**)	0.08 (−0.10 to 0.26)
	7–8	1.96 (1.81–2.11)	2.20 (2.08–2.32)	2.57 (2.44–2.69)	3.22 (3.08–3.36)	3.57 (3.33–3.82)	3.78 (3.52–4.03)	4.69 (4.41–4.97)	**0.39 (0.33–0.45**)	**0.40 (0.31–0.48**)	0.08 (−0.10 to 0.26)
	≥8	3.11 (2.88–3.34)	3.10 (2.92–3.28)	3.58 (3.38–3.79)	4.32 (4.11–4.53)	4.63 (4.28–4.99)	4.78 (4.42–5.14)	6.19 (5.76–6.62)	**0.37 (0.28–0.46**)	**0.44 (0.32–0.57**)	0.08 (−0.08 to 0.24)
Third and highest quartile	<6	9.33 (8.90–9.76)	12.17 (11.76–12.58)	14.48 (14.07–14.89)	18.62 (18.11–19.14)	20.42 (19.46–21.37)	20.98 (20.07–21.90)	24.01 (23.06–24.97)	**2.76 (2.56–2.97**)	**1.76 (1.43–2.09**)	**−1.00 (−1.39 to −0.62**)
	6–7	5.85 (5.59–6.11)	7.60 (7.35–7.86)	8.93 (8.67–9.20)	12.59 (12.21–12.97)	15.03 (14.30–15.76)	14.13 (13.44–14.81)	16.56 (15.80–17.32)	**1.84 (1.71–1.97**)	**1.25 (1.01–1.50**)	**−1.15 (−1.43 to −0.87**)
	7–8	5.00 (4.78–5.22)	7.21 (6.97–7.46)	8.49 (8.23–8.74)	12.09 (11.73–12.44)	11.53 (10.95–12.12)	12.96 (12.33–13.59)	15.68 (14.93–16.43)	**1.90 (1.79–2.01**)	**0.96 (0.74–1.19**)	**−1.15 (−1.43 to −0.87**)
	≥8	7.05 (6.77–7.33)	9.75 (9.43–10.06)	11.31 (10.98–11.65)	15.95 (15.50–16.39)	15.38 (14.67–16.10)	16.71 (15.92–17.51)	19.30 (18.44–20.15)	**2.43 (2.28–2.57**)	**1.02 (0.74–1.30**)	**−1.41 (−1.72 to −1.09**)

β values indicate the average annual change (%) in the weighted prevalence, multiplied by 100 for interpretability.

The values in bold font represent significant variance (*P* < .05).

β_diff_ = differences in β-coefficients, BMI = body mass index, CI = confidence interval, KCHS = Korea Community Health Survey.

* According to the Asian-Pacific guidelines, BMI is divided into 4 groups: underweight (<18.5 kg/m^2^), normal (18.5–22.9 kg/m^2^), overweight (23.0–24.9 kg/m^2^), and obese (≥25.0 kg/m^2^).

**Figure 2.**
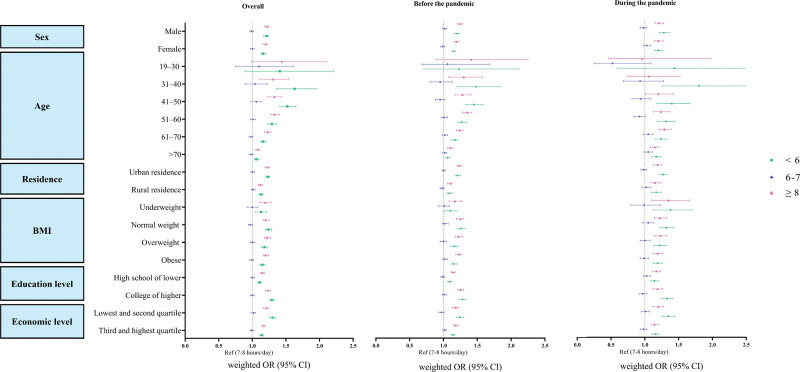
Forest plot of weighted odds ratios among individuals with both hypertension and diabetes by sleep duration. BMI = body mass index, OR = odds ratio, wOR = weighted odds ratio.

The risk of having both diseases, regardless of sex, was high among those who slept <6 hours (wOR, 1.20 [95% CI, 1.18–1.23]) or slept 8 hours or more (1.19 [1.17–1.22]). In addition, individuals living in urban areas (<6: 1.23 [1.20–1.26]; ≥8: 1.22 [1.19–1.26]) were more affected by sleep duration than those living in rural areas (<6: 1.13 [1.09–1.16]; ≥8: 1.12 [1.09–1.15]), and those with higher educational attainment (<6: 1.29 [1.25–1.33]); ≥8: 1.23 [1.19–1.27]) were more affected than those with lower educational levels attainment (<6: 1.11 [1.08–1.13]; ≥8: 1.15 [1.12–1.18]). This pattern remained consistent when comparing the periods before and during the COVID-19 pandemic (Fig. 2 and Table 3). Additionally, the risk of having hypertension was highest among those who slept <6 hours (1.23 [1.21–1.24]) followed by those who slept 6 to 7 hours (1.07 [1.06–1.08]) or >8 hours (1.05 [1.04–1.06]; Table S5, Supplemental Digital Content, https://links.lww.com/MD/Q692). The risk of having diabetes was high among those who slept >8 hours (1.21 [1.19–1.23]) or <6 hours (1.17 [1.15–1.19]; Tables S6 and S7, Supplemental Digital Content, https://links.lww.com/MD/Q692). Additionally, a significant interaction between sleep duration and during the COVID-19 pandemic was observed for all outcomes, indicating that the strength of the association between sleep duration and hypertension, diabetes, and their coexistence differed between the pre-pandemic and pandemic periods (Table S8, Supplemental Digital Content, https://links.lww.com/MD/Q692).

**Table 3:** Weighted odds ratios in the prevalence of individuals who have both hypertension and diabetes stratified by sleep duration before and during COVID-19, with 7 to 8 hours of sleep duration as the reference (weighted % [95% CI]).

Variables	Sleep duration (hours/day)	Overall (2009–2022)			Pre-pandemic era (2009–2019)			During pandemic era (2020–2022)		
		Sample size	wOR (95% CI)	*P*-value	Sample size	wOR (95% CI)	*P*-value	Sample size	wOR (95% CI)	*P*-value
Overall	7–8 (reference)	491,183	1.00 (reference)		392,601	1.00 (reference)		98,582	1.00 (reference)	
	<6	822,197	**1.20 (1.18–1.23**)	**<.001**	671,007	**1.18 (1.16–1.21**)	**<.001**	151,190	**1.25 (1.20–1.30**)	**<.001**
	6–7	914,450	1.00 (0.98–1.02)	.725	742,258	1.00 (0.98–1.02)	.820	172,192	0.99 (0.95–1.03)	.640
	≥8	676,057	**1.19 (1.17–1.22**)	**<.001**	548,619	**1.19 (1.17–1.22**)	**<.001**	127,438	**1.18 (1.14–1.23**)	**<.001**
Sex										
Male	7–8 (reference)	192,647	1.00 (reference)		155,797	1.00 (reference)		36,850	1.00 (reference)	
	<6	388,490	**1.21 (1.17–1.24**)	**<.001**	318,907	**1.20 (1.16–1.24**)	**<.001**	69,583	**1.28 (1.21–1.36**)	**<.001**
	6–7	423,760	0.99 (0.96–1.02)	.463	343,821	1.02 (0.99–1.05)	.198	79,939	0.98 (0.93–1.03)	.402
	≥8	301,539	**1.22 (1.18–1.25**)	**<.001**	242,616	**1.24 (1.20–1.28**)	**<.001**	58,923	**1.21 (1.15–1.28**)	**<.001**
Female	7–8 (reference)	298,536	1.00 (reference)		236,804	1.00 (reference)		61,732	1.00 (reference)	
	<6	433,707	**1.16 (1.13–1.20**)	**<.001**	352,100	**1.15 (1.12–1.18**)	**<.001**	81,607	**1.20 (1.14–1.26**)	**<.001**
	6–7	490,690	1.00 (0.97–1.03)	.967	398,437	0.99 (0.96–1.02)	.463	92,253	1.03 (0.98–1.09)	.271
	≥8	374,518	**1.20 (1.16–1.23**)	**<.001**	306,003	**1.19 (1.16–1.23**)	**<.001**	68,515	**1.20 (1.14–1.27**)	**<.001**
Age, yr										
19–30	7–8 (reference)	35,462	1.00 (reference)		30,197	1.00 (reference)		5265	1.00 (reference)	
	<6	92,503	1.41 (0.89–2.21)	.140	78,294	1.23 (0.71–2.12)	.461	14,209	1.44 (0.60–3.45)	.416
	6–7	125,725	1.10 (0.75–1.62)	.623	101,213	1.06 (0.68–1.68)	.788	24,512	0.52 (0.25–1.09)	.085
	≥8	103,547	1.44 (0.99–2.10)	.055	83,090	1.41 (0.89–2.25)	.143	20,457	0.96 (0.46–1.98)	.909
31–40	7–8 (reference)	46,987	1.00 (reference)		40,348	1.00 (reference)		6639	1.00 (reference)	
	<6	124,600	**1.63 (1.36–1.95**)	**<.001**	107,607	**1.48 (1.20–1.84**)	**<.001**	16,993	**1.80 (1.26–2.57**)	**.001**
	6–7	155,692	1.04 (0.89–1.22)	.596	133,222	0.95 (0.80–1.13)	.570	22,470	0.93 (0.68–1.28)	.647
	≥8	96,633	**1.31 (1.11–1.54**)	**.002**	82,825	**1.30 (1.07–1.58**)	**.009**	13,808	1.06 (0.74–1.52)	.744
41–50	7–8 (reference)	70,484	1.00 (reference)		59,445	1.00 (reference)		11,039	1.00 (reference)	
	<6	169,934	**1.52 (1.41–1.65**)	**<.001**	144,514	**1.45 (1.33–1.59**)	**<.001**	25,420	**1.40 (1.18–1.67**)	**<.001**
	6–7	183,765	1.06 (0.98–1.13)	.130	154,089	0.95 (0.88–1.02)	.157	29,676	0.94 (0.81–1.09)	.400
	≥8	97,802	**1.33 (1.23–1.44**)	**<.001**	82,328	**1.28 (1.17–1.40**)	**<.001**	15,474	**1.20 (1.01–1.42**)	**.037**
51–60	7–8 (reference)	96,383	1.00 (reference)		78,073	1.00 (reference)		18,310	1.00 (reference)	
	<6	172,901	**1.29 (1.23–1.36**)	**<.001**	140,297	**1.27 (1.21–1.34**)	**<.001**	32,604	**1.31 (1.19–1.44**)	**<.001**
	6–7	176,886	1.01 (0.97–1.05)	.662	143,617	1.01 (0.97–1.06)	.605	33,269	0.92 (0.84–1.01)	.070
	≥8	109,981	**1.33 (1.27–1.40**)	**<.001**	90,920	**1.35 (1.28–1.42**)	**<.001**	19,061	**1.24 (1.13–1.37**)	**<.001**
61–70	7–8 (reference)	105,233	1.00 (reference)		81,419	1.00 (reference)		23,814	1.00 (reference)	
	<6	136,363	**1.16 (1.12–1.20**)	**<.001**	104,682	**1.17 (1.12–1.21**)	**<.001**	31,681	**1.24 (1.16–1.33**)	**<.001**
	6–7	144,041	0.98 (0.95–1.01)	.178	111,874	1.02 (0.98–1.06)	.425	32,167	1.05 (0.98–1.12)	.191
	≥8	113,345	**1.23 (1.18–1.27**)	**<.001**	89,510	**1.24 (1.19–1.29**)	**<.001**	23,835	**1.29 (1.21–1.39**)	**<.001**
>70	7–8 (reference)	136,634	1.00 (reference)		103,119	1.00 (reference)		33,515	1.00 (reference)	
	<6	125,896	**1.06 (1.03–1.10**)	**.000**	95,613	**1.06 (1.02–1.10**)	**.003**	30,283	**1.17 (1.10–1.24**)	**<.001**
	6–7	128,341	0.98 (0.95–1.01)	.151	98,243	1.02 (0.98–1.05)	.424	30,098	1.05 (0.99–1.11)	.144
	≥8	154,749	**1.08 (1.05–1.11**)	**<.001**	119,946	**1.10 (1.06–1.14**)	**<.001**	34,803	**1.15 (1.08–1.21**)	**<.001**
Region of residence										
Urban	7–8 (reference)	276,481	1.00 (reference)		220,302	1.00 (reference)		56,179	1.00 (reference)	
	<6	501,126	**1.23 (1.20–1.26**)	**<.001**	406,992	**1.21 (1.18–1.25**)	**<.001**	94,134	**1.27 (1.21–1.33**)	**<.001**
	6–7	531,905	1.00 (0.98–1.03)	.897	426,033	1.00 (0.98–1.03)	.843	105,872	0.98 (0.94–1.03)	.427
	≥8	345,653	**1.22 (1.19–1.26**)	**<.001**	275,444	**1.23 (1.20–1.27**)	**<.001**	70,209	**1.19 (1.13–1.25**)	**<.001**
Rural	7–8 (reference)	214,702	1.00 (reference)		172,299	1.00 (reference)		42,403	1.00 (reference)	
	<6	321,071	**1.13 (1.09–1.16**)	**<.001**	264,015	**1.09 (1.05–1.13**)	**<.001**	57,056	**1.17 (1.10–1.24**)	**<.001**
	6–7	382,545	1.01 (0.99–1.04)	.338	316,225	0.98 (0.95–1.01)	.145	66,320	1.02 (0.96–1.09)	.492
	≥8	330,404	**1.12 (1.09–1.15**)	**<.001**	273,175	**1.10 (1.06–1.13**)	**<.001**	57,229	**1.15 (1.09–1.23**)	**<.001**
BMI group										
Underweight	7–8 (reference)	50,696	1.00 (reference)		44,538	1.00 (reference)		6158	1.00 (reference)	
	<6	55,426	**1.13 (1.05–1.21**)	**.002**	48,921	**1.10 (1.01–1.19**)	**.024**	6505	**1.38 (1.12–1.70**)	**.003**
	6–7	64,285	1.00 (0.92–1.08)	.959	56,448	1.01 (0.93–1.09)	.896	7837	0.99 (0.79–1.23)	.896
	≥8	70,457	**1.19 (1.11–1.28**)	**<.001**	62,236	**1.17 (1.08–1.27**)	**<.001**	8221	**1.35 (1.10–1.66**)	**.005**
Normal weight	7–8 (reference)	190,591	1.00 (reference)		152,963	1.00 (reference)		37,628	1.00 (reference)	
	<6	337,887	**1.24 (1.19–1.29**)	**<.001**	278,734	**1.26 (1.21–1.32**)	**<.001**	59,153	**1.32 (1.23–1.43**)	**<.001**
	6–7	395,981	0.97 (0.94–1.01)	.121	324,984	1.02 (0.98–1.07)	.277	70,997	1.05 (0.97–1.13)	.222
	≥8	289,932	**1.20 (1.16–1.25**)	**<.001**	237,113	**1.25 (1.19–1.30**)	**<.001**	52,819	**1.22 (1.14–1.32**)	**<.001**
Overweight	7–8 (reference)	111,151	1.00 (reference)		87,923	1.00 (reference)		23,228	1.00 (reference)	
	<6	199,563	**1.18 (1.13–1.22**)	**<.001**	161,973	**1.16 (1.10–1.21**)	**<.001**	37,590	**1.22 (1.13–1.32**)	**<.001**
	6–7	219,143	1.00 (0.97–1.04)	.929	177,053	1.00 (0.95–1.04)	.867	42,090	1.00 (0.93–1.08)	.946
	≥8	148,731	**1.22 (1.18–1.27**)	**<.001**	119,244	**1.22 (1.17–1.28**)	**<.001**	29,487	**1.23 (1.14–1.33**)	**<.001**
Obese	7–8 (reference)	138,745	1.00 (reference)		107,177	1.00 (reference)		31,568	1.00 (reference)	
	<6	229,321	**1.15 (1.11–1.19**)	**<.001**	181,379	**1.15 (1.11–1.20**)	**<.001**	47,942	**1.19 (1.12–1.26**)	**<.001**
	6–7	235,041	0.99 (0.96–1.02)	.348	183,773	1.02 (0.99–1.06)	.227	51,268	0.99 (0.93–1.05)	.729
	≥8	166,937	**1.20 (1.16–1.24**)	**<.001**	130,026	**1.23 (1.18–1.27**)	**<.001**	36,911	**1.19 (1.12–1.27**)	**<.001**
Education										
High school or lower education	7–8 (reference)	243,353	1.00 (reference)		195,854	1.00 (reference)		47,499	1.00 (reference)	
	<6	264,733	**1.11 (1.08–1.13**)	**<.001**	218,922	**1.09 (1.06–1.12**)	**<.001**	45,811	**1.14 (1.08–1.20**)	**<.001**
	6–7	277,275	1.00 (0.98–1.03)	.955	231,810	0.99 (0.96–1.02)	.543	45,465	1.03 (0.98–1.09)	.234
	≥8	273,406	**1.15 (1.12–1.18**)	**<.001**	227,329	**1.14 (1.11–1.18**)	**<.001**	46,077	**1.17 (1.11–1.23**)	**<.001**
College or higher education	7–8 (reference)	247,830	1.00 (reference)		196,747	1.00 (reference)		51,083	1.00 (reference)	
	<6	557,464	**1.29 (1.25–1.33**)	**<.001**	452,085	**1.28 (1.23–1.33**)	**<.001**	105,379	**1.33 (1.25–1.41**)	**<.001**
	6–7	637,175	1.00 (0.97–1.03)	.875	510,448	1.01 (0.98–1.05)	.498	126,727	0.97 (0.92–1.03)	.302
	≥8	402,651	**1.23 (1.19–1.27**)	**<.001**	321,290	**1.25 (1.21–1.30**)	**<.001**	81,361	**1.19 (1.12–1.26**)	**<.001**
Household income										
Lowest and second quartile	7–8 (reference)	182,027	1.00 (reference)		141,732	1.00 (reference)		40,295	1.00 (reference)	
	<6	415,045	**1.30 (1.25–1.35**)	**<.001**	329,336	**1.24 (1.19–1.30**)	**<.001**	85,709	**1.35 (1.26–1.44**)	**<.001**
	6–7	466,316	1.02 (0.98–1.05)	.325	363,980	0.97 (0.93–1.01)	.132	102,336	1.01 (0.95–1.07)	.805
	≥8	279,940	**1.21 (1.16–1.25**)	**<.001**	217,612	**1.18 (1.13–1.23**)	**<.001**	62,328	**1.20 (1.12–1.28**)	**<.001**
Third and highest quartile	7–8 (reference)	309,156	1.00 (reference)		250,869	1.00 (reference)		58,287	1.00 (reference)	
	<6	407,152	**1.14 (1.11–1.16**)	**<.001**	341,671	**1.14 (1.11–1.17**)	**<.001**	65,481	**1.16 (1.10–1.21**)	**<.001**
	6–7	448,134	0.99 (0.97–1.02)	.596	378,278	1.02 (0.99–1.04)	.277	69,856	0.98 (0.93–1.03)	.399
	≥8	396,117	**1.17 (1.14–1.19**)	**<.001**	331,007	**1.18 (1.15–1.22**)	**<.001**	65,110	**1.14 (1.09–1.20**)	**<.001**

The values in bold font represent significant variance (*P* < .05).

BMI = body mass index, CI = confidence interval, KCHS = Korea Community Health Survey, wOR = weighted odds ratios.

* According to the Asian-Pacific guidelines, BMI is divided into 4 groups: underweight (<18.5 kg/m^2^), normal (18.5–22.9 kg/m^2^), overweight (23.0–24.9 kg/m^2^), and obese (≥25.0 kg/m^2^).

## 4. Discussion

### 4.1. Key finding

This study analyzed trends in sleep duration and associated risk factors among individuals with hypertension, diabetes, and those with both conditions, using a large population dataset from 2009 to 2022. The number of individuals with hypertension, diabetes, and both conditions increased before and after the COVID-19 pandemic. Notably, this increase was more pronounced among those who slept <6 hours per day. The weighted prevalence of hypertension, diabetes, or both conditions was higher among individuals who slept <6 hours or >8 hours per day, with higher values observed in females. Additionally, individuals who slept <6 hours or >8 hours per day exhibited a higher wOR of diabetes and had both diabetes and hypertension. Moreover, the wOR for hypertension was elevated among those sleeping <6 hours, those sleeping 7 to 8 hours, and those sleeping >8 hours. Individuals living in urban areas and those with higher educational attainment were more affected by sleep duration in relation to the prevalence of diabetes and hypertension.

### 4.2. Plausible underlying mechanisms

The risk of hypertension, diabetes, and both diseases was high among individuals who slept <6 hours or >8 hours per day. Under normal sleep conditions, the vagal system is activated, and catecholamine biosynthesis decreases. However, short sleep duration acts as a stressor on the body and activates the sympathetic system, as evidenced by evaluations of serum stress hormones after sleep deprivation. Consequently, the rennin-angiotensin-aldosterone system is stimulated, and the synthesis of central catecholamines increases. This leads to blood vessel constriction, which raises blood pressure and potentially leads to hypertension.^[17]^ Additionally, chronic short sleep duration can reduce flow-mediated dilation of the arteries and intracellular magnesium concentrations. Since magnesium can decrease vascular tone, a magnesium deficiency can lead to arterial constriction. Therefore, short sleep duration can create a state of long-term vascular tension, which may contribute to the development of hypertension.^[17]^ Furthermore, prolonged exposure to hemodynamic load for 24 hours due to short sleep duration can lead to structural adaptations such as hypertrophic remodeling of the arteries and left ventricle. This can gradually induce the cardiovascular system to operate at a higher-pressure equilibrium. Therefore, short sleep duration can increase the risk of hypertension by disrupting circadian rhythmicity and autonomic balance.^[18]^

Long sleep duration can also increase the risk of hypertension. This is because long sleep duration can elevate the likelihood of obesity, which in turn raises the risk of developing hypertension.^[19]^ Short sleep duration can also increase the risk of developing diabetes. Although the mechanisms are not clearly understood, short sleep duration can alter insulin signaling in adipocytes, leading to insulin resistance, which can increase the risk of diabetes. Additionally, short sleep duration decreases leptin levels and increases ghrelin levels, promoting obesity and thereby increasing the risk of diabetes. Furthermore, the disruption of leptin and ghrelin can cause dysregulation of insulin and glucose, further increasing the risk of diabetes.^[8]^ Additionally, longer sleep duration was associated with higher levels of hemoglobin A1c and worsened glycemic control.^[10]^

### 4.3. Comparison of previous studies

Previous studies have suggested that sleep duration may be associated with metabolic disorders.^[17,18,20]^ Similar to our findings, they presented that short sleep duration increases the risk of metabolic disorders. However, those studies did not adequately examine long sleep duration^[10,21]^ or consider individuals with both conditions simultaneously. Additionally, previous studies have the limitation of having conducted analyses using data from a short period that did not include the COVID-19 era, and they were limited by relatively small sample sizes (n = 347,759,^[17]^ 335,727^[20]^) or were review articles in which the exact sample size could not be clearly determined. In contrast, using a larger sample size, this study provided a more clear examination of the risk associated with long sleep duration. Additionally, this study analyzed long-term data that included the COVID-19 period and investigated the association in individuals with comorbidities, providing a broader understanding of the relationship between sleep duration and metabolic disorders. This study also identified that the association is more severe when both conditions are present, leading to the proposal of relevant policies.

Additionally, some studies have investigated risk factors for hypertension and diabetes, proposing similar factors in our study.^[22,23]^ However, these previous studies did not analyze the association of risk factors on metabolic disorders simultaneously, nor did they examine the association of sleep duration on these risk factors.^[24,25]^ In contrast, our study simultaneously analyzed a large number of risk factors to estimate their combined associations. Furthermore, rather than merely identifying risk factors, this study also compared differences in sleep duration among individuals with these risk factors, suggesting that short or long sleep duration can further increase the associations with these factors.

### 4.4. Clinical and policy implications

While the growing recognition of sleep issues has led to efforts such as the convening of technical meetings on sleep and health, during which definitions and measurement indicators for sleep disorders were established. However, sleep-related issues are still not addressed in the National Health Policies, Strategies, and Plans. Based on our findings, we suggest that sleep should be considered an important health marker, alongside nutrition and physical activity, and that future policy discussions may benefit from incorporating sleep-related concerns. In addition, while the WHO has specified age-specific sleep duration recommendations for children under the age of 5, it has not established concrete sleep duration targets for adolescents and adults.^[26]^ Therefore, further work is needed to develop evidence-based guidelines for sleep duration in these age groups.

The International Labour Organization has established conventions and recommendations regarding night work; however, a major limitation is that these instruments lack legal binding force in most countries, thereby limiting their scope of protection.^[26]^ Previous studies have also suggested that excessive workloads and shift work negatively affect sleep.^[27,28]^ Therefore, these policies should be revised and implemented within national legal frameworks, and effective enforcement and monitoring mechanisms must be established to ensure their practical application.

### 4.5. Strengths and limitations

In this study, we conducted a long-term analysis of trends among individuals with hypertension, diabetes, and those with both conditions, based on their sleep duration, using a large sample size. We specifically examined the association between sleep duration and individuals with both diseases, and additionally explored the association between sleep duration and those with existing risk factors. As a result, this research significantly enhances our understanding of the association between sleep duration and metabolic disorders. Consequently, the findings of this study could contribute to the formulation of public health strategies to mitigate sleep-related problems. Nonetheless, despite these strengths, several limitations warrant consideration.

First, this study has the limitation of not including sleep quality among the variables considered. Although various previous studies have shown a close relationship between sleep duration and sleep quality, with the relationship between sleep duration and deterioration in sleep quality often following the same U-shaped curve observed in health outcomes, substituting sleep quality with sleep duration has its limitations.^[29,30]^ Therefore, future research that includes sleep quality could provide a more comprehensive understanding of the relationship between sleep duration and metabolic disorders. Second, as a cross-sectional study, this study has the limitation of being unable to present direct causal mechanisms. However, by referencing numerous previous studies, this research has established the reliability of these causal associations and proposed several potential mechanisms that may contribute to them. Nonetheless, the precise mechanisms remain unclear, underscoring the need for further research to elucidate them. Third, the data used in this study were entirely self-reported, which may not accurately reflect actual conditions. Since the data used in this study are self-report data, reporting bias is possible. However, the reliability of this data has been shown in numerous previous studies.^[31]^ Therefore, it is expected that it would be challenging to have a significant association with the reliability of our data. In addition, hypertension and diabetes were defined based on participants’ responses to KCHS. This survey-based dataset does not include international classification of diseases codes, laboratory results (e.g., fasting glucose or HbA1c), or prescription records. Although such clinical data were unavailable and some degree of misclassification may exist, particularly among the older population, the reliability of self-reported diagnoses is considered relatively high in the context of the South Korean healthcare system. This is largely attributed to the country’s comprehensive national health screening programs and routine medical checkups, which promote high levels of awareness and diagnosis of chronic conditions such as hypertension and diabetes.^[32]^ This aligns with previous studies utilizing South Korean survey datasets, which have shown that self-reported diagnoses are reasonably accurate among the Korean population.^[11]^ The KCHS is widely used in epidemiologic research, and its survey-based measures are generally regarded as valid and reliable. Fourth, this study has a limitation in that it addresses sleep duration only at the time of the survey, which restricts its ability to reflect long-term sleep duration fully. However, sleep duration tends to remain relatively stable over time,^[33]^ and numerous previous studies have suggested that sleep duration may be associated with the development of metabolic diseases.^[20,34]^ Fifth, this study could not address drug compliance or the achievement of treatment goals, which are important for evaluating the management of hypertension and diabetes. This limitation arises from the use of the KCHS, which does not include cohort data, follow-up information, or electronic medical records. Therefore, future studies incorporating these data sources are needed to assess treatment adherence and effectiveness more comprehensively. Considering these findings, we believe that our study provides valuable academic implications. Nonetheless, to more clearly elucidate the relationship between long-term sleep patterns and health outcomes, further research incorporating longitudinal assessments of sleep duration is warranted. Sixth, in this study, all participants with incomplete data were excluded, which may have introduced selection bias. While this exclusion process has the advantage of enhancing the internal validity of the analysis, it also presents the limitation of potentially restricting the generalizability of the findings. Therefore, these factors should be carefully considered when interpreting the results.

## 5. Conclusion

This study analyzed sleep duration trends and associated risk factors among individuals with hypertension, diabetes, and those with both conditions using a large population sample from 2009 to 2022, including the COVID-19 pandemic period. The number of individuals with hypertension, diabetes, and both conditions increased before and during the COVID-19 pandemic, particularly among those who sleep <6 hours per day. The prevalence of hypertension, diabetes, or both conditions was significantly associated with short sleep (<6 hours) and long sleep (>8 hours). Sleep duration was more strongly associated with the prevalence of comorbid diabetes and hypertension among those living in urban settings and those with more advanced educational backgrounds. Importantly, the use of a large, nationally representative survey enabled us to show long-term trends in the burden of these diseases and their associations with sleep duration. Based on these findings, our results support considering sleep as an important health indicator.

## Author contributions

**Conceptualization:** Jinyoung Jeong, Hyunjee Kim, Jaeyu Park, Dong Keon Yon.

**Data curation:** Jinyoung Jeong, Hyunjee Kim, Jaeyu Park, Dong Keon Yon.

**Formal analysis:** Jinyoung Jeong, Hyunjee Kim, Jaeyu Park, Dong Keon Yon.

**Funding acquisition:** Jaeyu Park, Ho Geol Woo, Dong Keon Yon.

**Investigation:** Jinyoung Jeong, Hyunjee Kim, Jaeyu Park, Dong Keon Yon.

**Methodology:** Jinyoung Jeong, Hyunjee Kim, Jaeyu Park, Dong Keon Yon.

**Project administration:** Jinyoung Jeong, Hyunjee Kim, Jaeyu Park, Dong Keon Yon.

**Resources:** Jinyoung Jeong, Hyunjee Kim, Jaeyu Park, Dong Keon Yon.

**Software:** Jinyoung Jeong, Hyunjee Kim, Jaeyu Park, Dong Keon Yon.

**Supervision:** Dong Keon Yon.

**Validation:** Jinyoung Jeong, Hyunjee Kim, Jaeyu Park, Dong Keon Yon.

**Visualization:** Jinyoung Jeong, Hyunjee Kim, Jaeyu Park, Dong Keon Yon.

**Writing – original draft:** Jinyoung Jeong, Hyunjee Kim, Jaeyu Park, Dong Keon Yon.

**Writing – review & editing:** Jinyoung Jeong, Hyunjee Kim, Jaeyu Park, Hyeon Jin Kim, Hyesu Jo, Sooji Lee, Kyeongmin Lee, Seoyoung Park, Yesol Yim, Ho Geol Woo, Yejun Son, Soeun Kim, Sang Youl Rhee, Dong Keon Yon.

## Supplementary Material


