# Supplementary material for: National trends in the prevalence of hypertension and diabetes stratified by sleep duration in South Korea, 2009–2022: A nationwide cross-sectional study: Trends and association of sleep duration in hypertension and diabetes
Source: Medicine (Baltimore). 2025 Nov 21;104(47):e46042. doi: 10.1097/MD.0000000000046042 (PMC12643665; doi:10.1097/MD.0000000000046042)
Supplement: Supplementary file 1 [file medi-104-e46042-s001.pdf]

Figure S1. Flowchart of population**.**

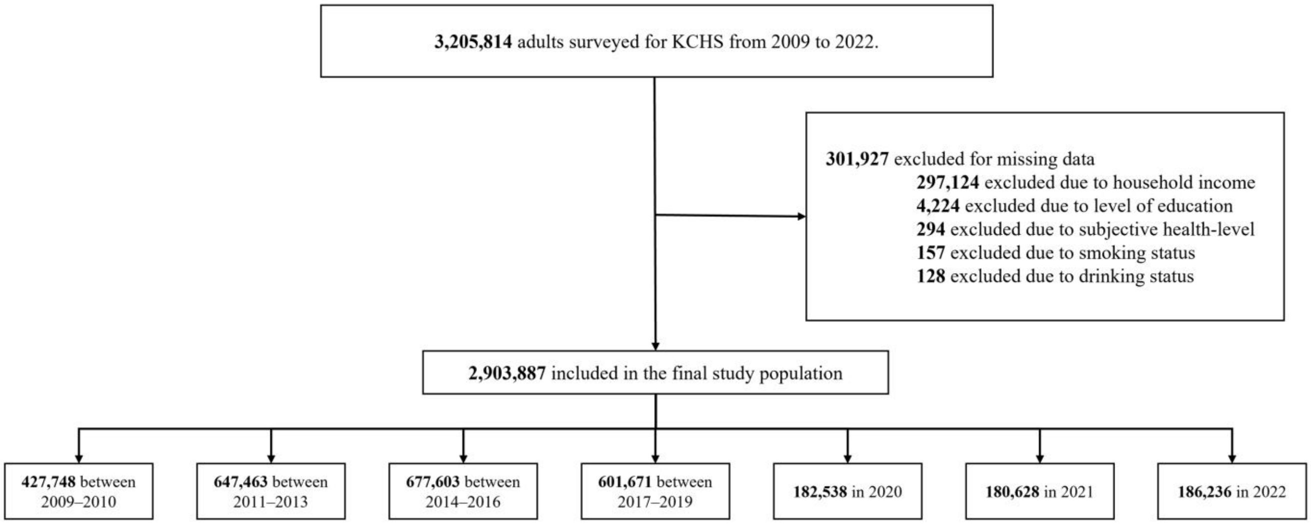

Table S1. Crude rate of baseline characteristics of Korean adults based on the data from KCHS, 2009–2022 (n=2,903,887).

Variables	Total	Pre-pandemic				During the pandemic		
		2009-2010	2011-2013	2014-2016	2017-2019	2020	2021	2022
**Overall, n**	2,903,887	427,748 (14.73)	647,463 (22.30)	677,603 (23.33)	601,671 (20.72)	182,538 (6.29)	180,628 (6.22)	186,236 (6.41)
**Sex, n (%)**								
Male	1,306,436 (44.99)	197,049 (46.18)	291,521 (45.09)	305,732 (45.40)	266,839 (44.77)	81,026 (44.93)	80,616 (45.12)	83,653 (45.34)
Female	1,597,451 (55.01)	230,699 (53.82)	355,942 (54.91)	371,871 (54.60)	334,832 (55.23)	101,512 (55.07)	100,012 (54.88)	102,583 (54.66)
**Age, years, n (%)**								
19–30	357,237 (12.30)	61,106 (17.53)	80,652 (15.85)	81,964 (15.65)	69,072 (14.76)	22,605 (15.67)	21,301 (14.71)	20,537 (13.86)
31–40	423,912 (14.60)	78,799 (21.52)	107,594 (20.08)	101,275 (18.24)	76,334 (15.68)	20,184 (13.80)	20,104 (13.93)	19,622 (13.15)
41–50	521,985 (17.98)	87,895 (21.88)	126,790 (21.83)	126,213 (21.07)	99,478 (18.93)	27,743 (17.46)	27,094 (17.29)	26,772 (16.47)
51–60	556,151 (19.15)	75,691 (16.78)	124,679 (18.58)	135,563 (19.50)	116,974 (19.62)	34,924 (19.61)	34,015 (19.44)	34,305 (19.06)
61–70	498,982 (17.18)	66,362 (15.51)	103,077 (15.92)	108,197 (15.97)	109,849 (18.26)	35,058 (19.21)	36,004 (19.93)	40,435 (21.71)
>70	545,620 (18.79)	57,895 (13.53)	104,671 (16.17)	124,391 (18.36)	129,964 (21.60)	42,024 (23.02)	42,110 (23.31)	44,565 (23.93)
**Region of residence, n (%)**								
Urban	1,655,165 (57.00)	236,023 (79.84)	362,373 (81.49)	381,575 (82.22)	348,800 (82.19)	107,081 (82.56)	107,685 (82.77)	111,628 (82.78)
Rural	1,248,722 (43.00)	191,725 (20.16)	285,090 (18.51)	296,028 (17.78)	252,871 (17.81)	75,457 (17.44)	72,943 (17.23)	74,608 (17.22)
**BMI group,[a] n (%)**								
Underweight	240,864 (8.29)	36,118 (7.11)	64,237 (7.68)	60,367 (6.82)	51,421 (6.84)	7,530 (4.13)	10,618 (4.97)	10,573 (4.90)
Normal weight	1,214,391 (41.82)	195,470 (47.06)	282,447 (45.56)	285,852 (44.16)	230,025 (39.94)	73,079 (40.95)	72,734 (40.98)	74,784 (40.94)
Overweight	678,588 (23.37)	99,899 (23.44)	149,141 (23.17)	157,512 (23.33)	139,641 (23.31)	43,828 (24.11)	43,550 (24.18)	45,017 (23.98)
Obese	770,044 (26.52)	96,261 (22.40)	151,638 (23.58)	173,872 (25.69)	180,584 (29.91)	58,101 (30.81)	53,726 (29.87)	55,862 (30.18)
**Average sleep duration, hours/day, n (%)**								
< 6	491,183 (16.91)	61,811 (14.38)	104,479 (16.06)	120,677 (17.49)	105,634 (17.19)	29,387 (15.36)	33,241 (17.50)	35,954 (18.40)
6–7	822,197 (28.31)	116,606 (29.02)	185,510 (30.67)	199,198 (31.48)	169,693 (29.97)	48,469 (27.83)	50,283 (29.19)	52,438 (29.45)

7–8	914,450 (31.49)	135,510 (32.23)	205,745 (32.04)	210,886 (31.54)	190,117 (32.16)	59,562 (33.57)	56,064 (32.05)	56,566 (31.65)
≥8	676,057 (23.28)	113,821 (24.37)	151,729 (21.22)	146,842 (19.49)	136,227 (20.68)	45,120 (23.23)	41,040 (21.25)	41,278 (20.50)
**Subjective Health-level, n (%)**								
High	1,122,606 (38.66)	178,248 (44.28)	253,292 (41.60)	248,809 (39.98)	210,658 (37.68)	86,211 (50.42)	71,906 (43.07)	73,482 (42.99)
Middle	1,186,764 (40.87)	161,306 (39.31)	256,151 (41.75)	284,481 (43.67)	261,740 (45.26)	70,205 (38.41)	76,509 (42.76)	76,372 (41.50)
Low	594,517 (20.47)	88,194 (16.41)	138,020 (16.65)	144,313 (16.34)	129,273 (17.06)	26,122 (11.16)	32,213 (14.17)	36,382 (15.51)
**Hypertension, n (%)**								
Yes	749,187 (25.80)	87,996 (17.81)	151,178 (19.59)	172,481 (21.10)	172,114 (23.78)	52,977 (24.29)	54,080 (24.99)	58,361 (26.64)
No	2,154,700 (74.20)	339,752 (82.19)	496,285 (80.41)	505,122 (78.90)	429,557 (76.22)	129,561 (75.71)	126,548 (75.01)	127,875 (73.36)
**Diabetes, n (%)**								
Yes	297,237 (10.24)	32,465 (6.70)	56,681 (7.46)	68,043 (8.46)	68,863 (9.73)	22,240 (10.45)	23,402 (11.04)	25,543 (11.95)
No	2,606,650 (89.76)	395,283 (93.30)	590,782 (92.54)	609,560 (91.54)	532,808 (90.27)	160,298 (89.55)	157,226 (88.96)	160,693 (88.05)
**Depression counseling, n (%)**								
Yes	33,868 (1.17)	4,079 (0.99)	5,859 (0.96)	7,670 (1.16)	7,354 (1.32)	2,501 (1.52)	3,049 (1.79)	3,356 (1.91)
No	2,870,019 (98.83)	423,669 (99.01)	641,604 (99.04)	669,933 (98.84)	594,317 (98.68)	180,037 (98.48)	177,579 (98.21)	182,880 (98.09)
**Stress counseling, n (%)**								
Yes	55,516 (1.91)	6,438 (1.58)	10,301 (1.70)	13,205 (2.02)	11,766 (2.13)	4,140 (2.58)	4,663 (2.87)	5,003 (2.94)
No	2,848,371 (98.09)	421,310 (98.42)	637,162 (98.30)	664,398 (97.98)	589,905 (97.87)	178,398 (97.42)	175,965 (97.13)	181,233 (97.06)
**Level of education, n (%)**								
Elementary school or lower education	725,481 (24.98)	116,155 (18.48)	171,803 (16.83)	168,536 (15.21)	146,745 (15.30)	42,571 (14.97)	39,649 (14.02)	40,022 (13.94)
Middle school	333,286 (11.48)	50,790 (10.57)	75,172 (10.05)	75,864 (9.44)	68,850 (9.82)	20,948 (9.82)	20,075 (9.38)	21,587 (9.90)
High school	840,949 (28.96)	127,215 (31.54)	187,754 (30.73)	193,145 (29.98)	171,783 (30.02)	53,315 (30.43)	53,164 (30.45)	54,573 (30.13)
College or higher education	1,004,171 (34.58)	133,588 (39.42)	212,734 (42.39)	240,058 (45.36)	214,293 (44.86)	65,704 (44.78)	67,740 (46.15)	70,054 (46.03)
**Household income, n (%)**								
Lowest quartile	589,665 (20.31)	57,525 (17.52)	120,253 (24.28)	109,127 (21.68)	144,288 (31.17)	48,487 (33.38)	51,416 (35.43)	58,569 (38.40)

Second quartile	753,663 (25.95)	108,752 (29.50)	171,030 (30.49)	183,514 (31.69)	158,171 (29.50)	44,691 (27.07)	43,403 (25.97)	44,102 (25.52)
Third quartile	1,007,970 (34.71)	175,458 (39.79)	231,360 (33.25)	239,209 (32.96)	191,477 (28.42)	58,027 (28.50)	56,636 (27.88)	55,803 (26.30)
Highest quartile	552,589 (19.03)	86,013 (13.18)	124,820 (11.97)	145,753 (13.68)	107,735 (10.91)	31,333 (11.05)	29,173 (10.72)	27,762 (9.78)
**Smoking status, n (%)**								
Smoker	552,399 (19.02)	98,323 (23.14)	134,723 (21.19)	128,400 (19.46)	102,771 (17.68)	29,278 (16.44)	28,666 (16.17)	30,238 (16.46)
Ex-smoker	506,439 (17.44)	60,601 (13.92)	105,836 (15.84)	120,706 (16.94)	112,896 (18.00)	32,634 (17.84)	33,715 (18.34)	40,051 (21.21)
Non-smoker	1,845,049 (63.54)	268,824 (62.95)	406,904 (62.98)	428,497 (63.60)	386,004 (64.33)	120,626 (65.72)	118,247 (65.50)	115,947 (62.33)
**Alcohol consumption, n (%)**								
Non-drinker and 1day/month	1,728,869 (59.54)	254,732 (57.00)	376,119 (54.82)	384,774 (53.33)	354,666 (55.58)	119,372 (62.53)	120,060 (64.12)	119,146 (61.33)
2–9 days/month	965,649 (33.25)	142,742 (36.69)	221,718 (38.47)	238,785 (39.71)	201,812 (37.62)	53,299 (32.57)	50,985 (31.11)	56,308 (33.40)
Over 10 days/month	209,369 (7.21)	30,274 (6.31)	49,626 (6.72)	54,044 (6.97)	45,193 (6.80)	9,867 (4.90)	9,583 (4.77)	10,782 (5.27)

Abbreviations: BMI, body mass index; CI, confidence interval; KCHS, Korea Community Health Survey.

[a] According to the Asian-Pacific guidelines, BMI is divided into four groups: underweight (<18.5 kg/ m^2), normal (18.5–22.9 kg/ m^2), overweight (23.0–24.9 kg/ m^2), and obese (≥25.0 kg/ m^2).

Table S2. National trends in sleep duration among individuals with hypertension and β-coefficients of odds ratios before and during the COVID–19 pandemic (weighted % [95% CI]).

Variables	Sleep duration (hours/day)	Pre-pandemic				During the pandemic			Trends in the pre-pandemic era, β (95% CI)	Trends in the pandemic era, β (95% CI)	β_{diff} between 2009-2019 and 2019-2022 (95% CI)
		2009-2010	2011-2013	2014-2016	2017-2019	2020	2021	2022			
**Overall**	<6	25.03 (24.59 to 25.47)	26.95 (26.60 to 27.31)	28.36 (28.03 to 28.70)	31.61 (31.25 to 31.98)	33.47 (32.80 to 34.15)	34.76 (34.13 to 35.40)	36.27 (35.67 to 36.88)	**1.95 (1.78 to 2.13)**	**1.66 (1.43 to 1.89)**	**-0.29 (-0.58 to 0.00)**
	6–7	17.10 (16.83 to 17.38)	18.43 (18.20 to 18.65)	19.85 (19.62 to 20.08)	22.37 (22.12 to 22.63)	24.65 (24.19 to 25.12)	24.38 (23.92 to 24.83)	25.42 (24.97 to 25.87)	**1.57 (1.46 to 1.68)**	1.09 (0.93 to 1.25)	**-0.48 (-0.67 to -0.28)**
	7–8	14.91 (14.66 to 15.16)	16.63 (16.42 to 16.85)	18.04 (17.82 to 18.25)	20.40 (20.16 to 20.64)	20.32 (19.92 to 20.72)	20.62 (20.22 to 21.02)	22.29 (21.88 to 22.71)	**1.58 (1.47 to 1.68)**	**0.58 (0.43 to 0.72)**	**-1.00 (-1.18 to -0.82)**
	≥8	18.24 (17.94 to 18.55)	20.17 (19.89 to 20.44)	21.57 (21.28 to 21.86)	24.56 (24.25 to 24.86)	23.51 (23.02 to 24.00)	24.37 (23.85 to 24.89)	26.46 (25.92 to 27.00)	**1.75 (1.62 to 1.88)**	**0.59 (0.40 to 0.78)**	**-1.16 (-1.39 to -0.93)**
**Sex**											
Male	<6	21.08 (20.46 to 21.71)	24.00 (23.48 to 24.52)	25.91 (25.40 to 26.41)	29.63 (29.06 to 30.20)	31.02 (29.97 to 32.06)	32.40 (31.42 to 33.37)	34.11 (33.16 to 35.07)	**2.61 (2.35 to 2.87)**	**1.66 (1.31 to 2.01)**	**-0.95 (-1.38 to -0.51)**
	6–7	16.44 (16.06 to 16.82)	18.44 (18.12 to 18.76)	20.34 (20.02 to 20.66)	22.89 (22.52 to 23.25)	25.22 (24.53 to 25.92)	24.74 (24.08 to 25.40)	26.80 (26.14 to 27.46)	**1.95 (1.79 to 2.11)**	**1.33 (1.10 to 1.55)**	**-0.62 (-0.90 to -0.34)**
	7–8	15.48 (15.13 to 15.84)	18.08 (17.77 to 18.39)	20.16 (19.83 to 20.48)	22.19 (21.84 to 22.54)	21.39 (20.81 to 21.97)	22.67 (22.07 to 23.27)	24.59 (23.98 to 25.20)	**1.94 (1.79 to 2.09)**	**0.75 (0.54 to 0.96)**	**-1.20 (-1.45 to -0.94)**
	≥8	20.20 (19.74 to 20.66)	23.07 (22.64 to 23.50)	25.36 (24.92 to 25.81)	28.23 (27.76 to 28.70)	26.78 (26.04 to 27.52)	28.48 (27.68 to 29.28)	29.67 (28.86 to 30.47)	**2.32 (2.13 to 2.52)**	**0.65 (0.37 to 0.93)**	**-1.68 (-2.02 to -1.34)**

Female	<6	28.04 (27.44 to 28.64)	29.03 (28.57 to 29.49)	30.08 (29.64 to 30.52)	32.89 (32.43 to 33.36)	35.01 (34.17 to 35.85)	36.25 (35.46 to 37.05)	37.62 (36.86 to 38.38)	**1.32 (1.08 to 1.55)**	**1.63 (1.34 to 1.92)**	0.31 (-0.06 to 0.68)
	6–7	17.75 (17.37 to 18.13)	18.42 (18.11 to 18.72)	19.39 (19.08 to 19.69)	21.91 (21.57 to 22.25)	24.16 (23.56 to 24.76)	24.05 (23.45 to 24.65)	24.17 (23.59 to 24.76)	**1.15 (1.00 to 1.30)**	**0.85 (0.64 to 1.06)**	**-0.30 (-0.56 to -0.04)**
	7–8	14.40 (14.07 to 14.72)	15.40 (15.13 to 15.67)	16.21 (15.93 to 16.48)	18.86 (18.56 to 19.16)	19.39 (18.87 to 19.90)	18.80 (18.29 to 19.32)	20.28 (19.74 to 20.81)	**1.20 (1.07 to 1.33)**	**0.41 (0.22 to 0.59)**	**-0.79 (-1.02 to -0.56)**
	≥8	16.76 (16.38 to 17.14)	18.08 (17.74 to 18.41)	18.72 (18.37 to 19.07)	21.74 (21.37 to 22.12)	20.85 (20.24 to 21.46)	21.02 (20.38 to 21.67)	23.72 (23.04 to 24.40)	**1.22 (1.06 to 1.38)**	**0.47 (0.25 to 0.70)**	**-0.75 (-1.03 to -0.47)**
**Age, years**											
19–30	<6	2.40 (1.88 to 2.93)	2.04 (1.69 to 2.39)	2.25 (1.90 to 2.60)	1.88 (1.52 to 2.24)	2.11 (1.46 to 2.76)	1.88 (1.07 to 2.68)	3.44 (2.47 to 4.42)	-0.12 (-0.32 to 0.09)	**0.38 (0.06 to 0.70)**	**0.50 (0.12 to 0.88)**
	6–7	1.15 (0.96 to 1.34)	1.66 (1.46 to 1.86)	1.80 (1.59 to 2.00)	1.44 (1.24 to 1.64)	1.68 (1.22 to 2.13)	1.89 (1.45 to 2.34)	2.05 (1.61 to 2.49)	0.07 (-0.03 to 0.17)	**0.21 (0.05 to 0.38)**	0.14 (-0.05 to 0.33)
	7–8	1.13 (0.95 to 1.30)	1.23 (1.07 to 1.39)	1.40 (1.24 to 1.56)	1.27 (1.10 to 1.44)	1.08 (0.83 to 1.32)	1.13 (0.88 to 1.38)	1.33 (1.05 to 1.60)	0.05 (-0.03 to 0.13)	-0.02 (-0.12 to 0.09)	-0.07 (-0.20 to 0.07)
	≥8	1.01 (0.83 to 1.19)	1.32 (1.13 to 1.50)	1.55 (1.35 to 1.74)	1.19 (1.01 to 1.37)	1.16 (0.88 to 1.43)	1.41 (1.06 to 1.75)	1.32 (1.02 to 1.62)	0.08 (-0.02 to 0.17)	0.06 (-0.07 to 0.19)	-0.02 (-0.17 to 0.14)
31–40	<6	4.29 (3.77 to 4.82)	4.75 (4.28 to 5.22)	5.37 (4.91 to 5.83)	5.56 (4.99 to 6.13)	5.61 (4.49 to 6.73)	5.77 (4.74 to 6.79)	6.81 (5.68 to 7.94)	**0.44 (0.19 to 0.70)**	0.33 (-0.07 to 0.74)	-0.11 (-0.59 to 0.37)
	6–7	3.78 (3.48 to 4.09)	3.85 (3.59 to 4.10)	4.41 (4.14 to 4.68)	4.10 (3.80 to 4.40)	4.55 (3.93 to 5.17)	4.64 (4.02 to 5.25)	4.68 (4.04 to 5.32)	**0.18 (0.04 to 0.32)**	0.21 (-0.01 to 0.44)	0.03 (-0.23 to 0.30)

	7–8	2.96 (2.71 to 3.20)	3.15 (2.94 to 3.36)	3.45 (3.22 to 3.68)	3.04 (2.79 to 3.28)	3.74 (3.26 to 4.21)	4.34 (3.81 to 4.88)	3.82 (3.34 to 4.31)	0.07 (-0.04 to 0.19)	**0.37 (0.19 to 0.55)**	**0.30 (0.09 to 0.52)**
	≥8	2.67 (2.39 to 2.94)	2.77 (2.51 to 3.02)	2.69 (2.43 to 2.95)	2.93 (2.62 to 3.24)	3.50 (2.90 to 4.10)	3.76 (3.16 to 4.36)	3.36 (2.76 to 3.96)	0.08 (-0.05 to 0.22)	0.20 (-0.02 to 0.42)	0.12 (-0.14 to 0.38)
41–50	<6	12.09 (11.34 to 12.83)	12.48 (11.86 to 13.10)	12.60 (12.02 to 13.19)	12.83 (12.15 to 13.51)	14.57 (13.23 to 15.91)	14.26 (12.96 to 15.55)	15.54 (14.20 to 16.88)	0.23 (-0.10 to 0.56)	**0.94 (0.46 to 1.41)**	**0.70 (0.12 to 1.28)**
	6–7	10.44 (10.00 to 10.88)	10.78 (10.42 to 11.15)	10.99 (10.63 to 11.35)	11.49 (11.07 to 11.91)	12.45 (11.61 to 13.29)	13.02 (12.17 to 13.87)	12.61 (11.79 to 13.42)	**0.39 (0.19 to 0.59)**	**0.52 (0.22 to 0.82)**	0.13 (-0.23 to 0.49)
	7–8	9.92 (9.50 to 10.34)	9.63 (9.29 to 9.97)	9.63 (9.29 to 9.97)	10.08 (9.70 to 10.46)	10.76 (10.05 to 11.46)	10.92 (10.20 to 11.64)	11.21 (10.46 to 11.96)	0.16 (-0.03 to 0.35)	**0.39 (0.11 to 0.66)**	0.23 (-0.11 to 0.56)
	≥8	10.87 (10.30 to 11.43)	10.26 (9.77 to 10.74)	9.92 (9.41 to 10.44)	10.02 (9.47 to 10.57)	11.67 (10.65 to 12.70)	11.69 (10.64 to 12.74)	10.16 (9.19 to 11.14)	-0.19 (-0.46 to 0.08)	0.14 (-0.23 to 0.52)	0.34 (-0.12 to 0.79)
51–60	<6	26.51 (25.49 to 27.52)	26.96 (26.19 to 27.73)	27.19 (26.48 to 27.91)	27.82 (27.05 to 28.58)	26.93 (25.53 to 28.32)	29.02 (27.63 to 30.40)	28.12 (26.82 to 29.42)	**0.51 (0.09 to 0.92)**	0.16 (-0.35 to 0.67)	-0.35 (-1.00 to 0.31)
	6–7	25.12 (24.38 to 25.86)	25.16 (24.59 to 25.72)	24.72 (24.20 to 25.25)	25.38 (24.82 to 25.94)	24.83 (23.86 to 25.80)	24.96 (24.04 to 25.89)	24.93 (24.00 to 25.87)	0.18 (-0.12 to 0.48)	-0.12 (-0.49 to 0.25)	-0.30 (-0.77 to 0.18)
	7–8	24.02 (23.30 to 24.73)	24.66 (24.10 to 25.22)	24.37 (23.84 to 24.91)	24.19 (23.64 to 24.74)	24.17 (23.21 to 25.13)	23.32 (22.37 to 24.27)	24.59 (23.62 to 25.56)	0.09 (-0.20 to 0.39)	-0.02 (-0.38 to 0.35)	-0.11 (-0.58 to 0.36)
	≥8	26.71 (25.86 to 27.56)	26.67 (25.95 to 27.39)	27.10 (26.35 to 27.84)	25.90 (25.13 to 26.66)	25.98 (24.70 to 27.26)	24.49 (23.20 to 25.78)	26.97 (25.61 to 28.33)	-0.14 (-0.52 to 0.24)	0.14 (-0.38 to 0.65)	0.28 (-0.36 to 0.92)

61–70	<6	45.30 (44.17 to 46.43)	45.76 (44.85 to 46.67)	46.22 (45.36 to 47.07)	44.82 (43.97 to 45.66)	44.19 (42.71 to 45.66)	43.75 (42.43 to 45.07)	45.40 (44.15 to 46.64)	-0.20 (-0.66 to 0.25)	0.02 (-0.48 to 0.53)	0.23 (-0.46 to 0.91)
	6–7	42.65 (41.66 to 43.64)	44.25 (43.45 to 45.04)	44.39 (43.62 to 45.15)	42.62 (41.88 to 43.35)	41.80 (40.58 to 43.02)	39.79 (38.63 to 40.95)	41.38 (40.28 to 42.49)	-0.16 (-0.55 to 0.24)	**-0.63 (-1.07 to -0.19)**	-0.47 (-1.06 to 0.12)
	7–8	41.57 (40.62 to 42.53)	43.24 (42.45 to 44.02)	43.50 (42.75 to 44.25)	42.74 (42.03 to 43.45)	40.26 (39.08 to 41.45)	40.25 (39.05 to 41.45)	41.18 (40.03 to 42.32)	0.31 (-0.08 to 0.69)	**-0.67 (-1.12 to -0.22)**	**-0.98 (-1.57 to - 0.39)**
	≥8	43.71 (42.70 to 44.73)	44.81 (43.92 to 45.69)	44.50 (43.60 to 45.40)	45.54 (44.68 to 46.40)	41.88 (40.50 to 43.27)	43.40 (41.97 to 44.83)	43.58 (42.23 to 44.92)	**0.62 (0.18 to 1.06)**	**-0.67 (-1.20 to -0.13)**	**-1.28 (-1.98 to - 0.59)**
>70	<6	52.11 (50.95 to 53.26)	56.96 (56.11 to 57.81)	59.29 (58.52 to 60.06)	61.18 (60.43 to 61.92)	60.12 (58.84 to 61.39)	60.68 (59.49 to 61.87)	61.46 (60.38 to 62.53)	**2.70 (2.27 to 3.13)**	-0.14 (-0.58 to 0.30)	**-2.84 (-3.46 to - 2.23)**
	6–7	51.22 (50.04 to 52.39)	53.60 (52.70 to 54.50)	58.15 (57.34 to 58.97)	59.21 (58.42 to 60.00)	43.31 (42.00 to 44.61)	57.28 (56.02 to 58.54)	58.03 (56.79 to 59.27)	**2.74 (2.29 to 3.18)**	**-0.51 (-1.00 to -0.03)**	**-3.25 (-3.91 to - 2.60)**
	7–8	50.66 (49.42 to 51.91)	54.33 (53.41 to 55.24)	56.06 (55.23 to 56.89)	58.37 (57.60 to 59.15)	55.49 (54.20 to 56.78)	56.70 (55.40 to 58.00)	58.91 (57.64 to 60.19)	**2.35 (1.90 to 2.80)**	0.01 (-0.49 to 0.50)	**-2.34 (-3.01 to - 1.68)**
	≥8	48.92 (47.91 to 49.93)	53.31 (52.48 to 54.13)	55.39 (54.60 to 56.17)	58.49 (57.76 to 59.22)	56.10 (54.88 to 57.32)	56.86 (55.61 to 58.10)	58.09 (56.87 to 59.30)	**2.95 (2.55 to 3.34)**	-0.31 (-0.77 to 0.15)	**-3.25 (-3.86 to - 2.65)**
**Region of residence**											
Urban	<6	23.62 (23.10 to 24.13)	25.33 (24.93 to 25.74)	26.69 (26.30 to 27.08)	29.95 (29.53 to 30.37)	31.77 (30.99 to 32.55)	33.43 (32.70 to 34.16)	35.04 (34.34 to 35.73)	**1.88 (1.68 to 2.08)**	**1.73 (1.47 to 2.00)**	-0.15 (-0.48 to 0.19)
	6–7	15.97 (15.66 to 16.28)	17.13 (16.88 to 17.38)	18.57 (18.31 to 18.82)	21.03 (20.74 to 21.31)	23.41 (22.89 to 23.94)	23.04 (22.53 to 23.55)	24.25 (23.74 to 24.75)	**1.50 (1.38 to 1.63)**	**1.09 (0.91 to 1.27)**	**-0.41 (-0.63 to - 0.19)**

	7–8	13.75 (13.46 to 14.03)	15.26 (15.02 to 15.50)	16.74 (16.49 to 16.98)	18.99 (18.72 to 19.26)	18.86 (18.42 to 19.31)	19.27 (18.82 to 19.71)	21.10 (20.63 to 21.57)	**1.50 (1.38 to 1.62)**	**0.59 (0.43 to 0.75)**	**-0.91 (-1.11 to -0.71)**
	≥8	16.49 (16.13 to 16.85)	18.17 (17.85 to 18.50)	19.62 (19.29 to 19.96)	22.42 (22.07 to 22.78)	21.73 (21.17 to 22.30)	22.44 (21.84 to 23.04)	24.52 (23.90 to 25.15)	**1.63 (1.48 to 1.79)**	**0.58 (0.37 to 0.80)**	**-1.05 (-1.32 to -0.79)**
Rural	<6	30.70 (29.91 to 31.49)	34.24 (33.60 to 34.87)	36.03 (35.41 to 36.66)	39.13 (38.43 to 39.82)	41.02 (39.81 to 42.23)	40.59 (39.38 to 41.79)	41.79 (40.69 to 42.90)	**2.38 (2.05 to 2.70)**	**1.26 (0.84 to 1.68)**	**-1.12 (-1.65 to -0.59)**
	6–7	22.36 (21.77 to 22.94)	25.08 (24.59 to 25.57)	26.69 (26.19 to 27.18)	29.46 (28.90 to 30.02)	31.16 (30.19 to 32.13)	31.41 (30.41 to 32.42)	31.60 (30.67 to 32.52)	**2.05 (1.81 to 2.29)**	**1.09 (0.76 to 1.42)**	**-0.96 (-1.37 to -0.55)**
	7–8	19.66 (19.17 to 20.15)	22.80 (22.37 to 23.22)	24.21 (23.76 to 24.65)	27.15 (26.63 to 27.66)	27.56 (26.74 to 28.38)	27.67 (26.81 to 28.52)	28.44 (27.49 to 29.40)	**2.12 (1.91 to 2.33)**	**0.71 (0.40 to 1.01)**	**-1.41 (-1.78 to -1.04)**
	≥8	23.82 (23.30 to 24.34)	27.13 (26.63 to 27.62)	28.69 (28.17 to 29.21)	32.53 (31.95 to 33.10)	30.87 (29.94 to 31.80)	32.36 (31.39 to 33.33)	34.51 (33.49 to 35.53)	**2.39 (2.16 to 2.62)**	**0.91 (0.56 to 1.25)**	**-1.48 (-1.90 to -1.07)**
**BMI group [a]**											
Underweight	<6	25.26 (23.88 to 26.63)	30.98 (29.90 to 32.06)	30.31 (29.25 to 31.38)	31.47 (30.27 to 32.68)	22.03 (19.42 to 24.64)	28.25 (25.95 to 30.54)	28.71 (26.43 to 30.98)	**1.25 (0.68 to 1.82)**	**-0.87 (-1.67 to -0.06)**	**-2.12 (-3.10 to -1.14)**
	6–7	13.61 (12.74 to 14.49)	18.00 (17.22 to 18.78)	17.07 (16.29 to 17.84)	18.92 (18.05 to 19.79)	10.97 (9.29 to 12.65)	16.10 (14.44 to 17.76)	14.16 (12.59 to 15.73)	**1.04 (0.68 to 1.40)**	**-1.39 (-1.90 to -0.88)**	**-2.43 (-3.06 to -1.80)**
	7–8	9.98 (9.28 to 10.68)	14.62 (13.97 to 15.27)	14.58 (13.93 to 15.23)	16.97 (16.20 to 17.74)	8.48 (7.22 to 9.73)	11.74 (10.52 to 12.96)	11.16 (9.87 to 12.45)	**1.62 (1.32 to 1.93)**	**-1.79 (-2.21 to -1.36)**	**-3.41 (-3.93 to -2.89)**
	≥8	14.63 (13.85 to 15.41)	20.19 (19.43 to 20.95)	20.02 (19.22 to 20.82)	22.53 (21.67 to 23.38)	10.65 (9.29 to 12.02)	16.77 (15.26 to 18.28)	16.73 (15.17 to 18.30)	**1.75 (1.41 to 2.09)**	**-1.92 (-2.41 to -1.42)**	**-3.66 (-4.27 to -3.06)**

Normal weight	<6	18.55 (17.97 to 19.14)	20.05 (19.57 to 20.54)	21.28 (20.81 to 21.74)	22.77 (22.24 to 23.31)	26.19 (25.20 to 27.18)	27.64 (26.71 to 28.57)	28.71 (27.80 to 29.62)	**1.28 (1.04 to 1.51)**	**2.01 (1.68 to 2.33)**	**0.73 (0.32 to 1.13)**
	6–7	11.53 (11.19 to 11.87)	12.13 (11.85 to 12.41)	13.14 (12.86 to 13.43)	14.11 (13.78 to 14.44)	17.17 (16.56 to 17.77)	17.00 (16.39 to 17.62)	17.91 (17.31 to 18.51)	**0.77 (0.64 to 0.91)**	**1.26 (1.05 to 1.46)**	**0.49 (0.24 to 0.73)**
	7–8	9.33 (9.05 to 9.61)	10.72 (10.47 to 10.97)	11.35 (11.09 to 11.60)	12.63 (12.33 to 12.92)	12.98 (12.48 to 13.47)	13.73 (13.23 to 14.24)	15.15 (14.60 to 15.69)	**0.87 (0.75 to 0.99)**	**0.74 (0.57 to 0.92)**	-0.13 (-0.34 to 0.08)
	≥8	12.08 (11.72 to 12.44)	13.41 (13.08 to 13.74)	14.54 (14.19 to 14.90)	16.09 (15.70 to 16.49)	16.36 (15.73 to 16.99)	17.10 (16.43 to 17.77)	19.46 (18.74 to 20.19)	**1.11 (0.95 to 1.26)**	**0.99 (0.75 to 1.22)**	-0.12 (-0.40 to 0.16)
Overweight	<6	27.21 (26.29 to 28.13)	28.71 (27.97 to 29.44)	30.12 (29.41 to 30.82)	32.69 (31.94 to 33.44)	34.63 (33.28 to 35.98)	36.73 (35.43 to 38.04)	38.23 (36.98 to 39.47)	**1.50 (1.13 to 1.86)**	**2.05 (1.59 to 2.52)**	0.56 (-0.03 to 1.15)
	6–7	19.56 (18.98 to 20.13)	21.28 (20.80 to 21.75)	22.24 (21.77 to 22.71)	24.21 (23.69 to 24.73)	26.77 (25.83 to 27.71)	25.32 (24.44 to 26.20)	27.75 (26.83 to 28.66)	**1.34 (1.10 to 1.57)**	**1.02 (0.69 to 1.34)**	-0.32 (-0.72 to 0.08)
	7–8	17.85 (17.32 to 18.37)	20.25 (19.79 to 20.70)	21.63 (21.16 to 22.10)	22.72 (22.23 to 23.21)	23.07 (22.25 to 23.89)	23.65 (22.80 to 24.50)	25.71 (24.85 to 26.57)	**1.35 (1.13 to 1.57)**	**0.86 (0.56 to 1.16)**	**-0.50 (-0.87 to -0.12)**
	≥8	22.38 (21.71 to 23.05)	24.18 (23.56 to 24.79)	25.15 (24.53 to 25.78)	27.77 (27.11 to 28.43)	27.19 (26.15 to 28.24)	28.22 (27.09 to 29.35)	30.19 (29.07 to 31.30)	**1.46 (1.17 to 1.74)**	**0.87 (0.47 to 1.26)**	**-0.59 (-1.08 to -0.10)**
Obese	<6	33.98 (33.04 to 34.92)	34.88 (34.14 to 35.62)	36.46 (35.79 to 37.12)	40.80 (40.12 to 41.47)	41.95 (40.74 to 43.15)	42.85 (41.69 to 44.02)	45.08 (43.95 to 46.21)	**2.19 (1.83 to 2.55)**	**1.52 (1.09 to 1.95)**	**-0.67 (-1.23 to -0.11)**
	6–7	26.28 (25.63 to 26.93)	27.11 (26.59 to 27.62)	28.97 (28.48 to 29.46)	31.90 (31.40 to 32.41)	33.73 (32.83 to 34.62)	33.98 (33.10 to 34.85)	34.77 (33.90 to 35.64)	**1.71 (1.46 to 1.96)**	**1.19 (0.87 to 1.51)**	**-0.52 (-0.92 to -0.12)**

	7–8	25.97 (25.32 to 26.62)	25.98 (25.46 to 26.49)	28.20 (27.70 to 28.70)	30.51 (30.01 to 31.01)	30.08 (29.26 to 30.89)	29.87 (29.04 to 30.69)	31.56 (30.72 to 32.39)	**1.47 (1.22 to 1.72)**	**0.35 (0.05 to 0.66)**	**-1.12 (-1.51 to -0.73)**
	≥8	29.74 (28.98 to 30.51)	30.99 (30.32 to 31.65)	32.65 (31.99 to 33.31)	35.38 (34.75 to 36.02)	33.70 (32.69 to 34.71)	33.98 (32.89 to 35.06)	35.97 (34.90 to 37.04)	**1.58 (1.28 to 1.89)**	0.10 (-0.28 to 0.49)	**-1.48 (-1.97 to -0.98)**
**Subjective Health-level**											
High	<6	12.04 (11.48 to 12.60)	14.08 (13.60 to 14.56)	14.98 (14.51 to 15.46)	18.14 (17.57 to 18.71)	21.78 (20.84 to 22.71)	22.66 (21.68 to 23.64)	21.80 (20.86 to 22.74)	**1.67 (1.44 to 1.91)**	**1.45 (1.11 to 1.79)**	-0.22 (-0.63 to 0.19)
	6–7	8.82 (8.52 to 9.13)	10.26 (9.99 to 10.53)	11.05 (10.78 to 11.33)	12.89 (12.57 to 13.22)	16.53 (15.97 to 17.08)	15.65 (15.06 to 16.24)	16.21 (15.63 to 16.80)	**1.10 (0.97 to 1.23)**	**1.18 (0.98 to 1.38)**	0.08 (-0.16 to 0.32)
	7–8	7.59 (7.33 to 7.85)	9.33 (9.09 to 9.58)	9.99 (9.74 to 10.24)	11.45 (11.17 to 11.73)	13.33 (12.89 to 13.78)	13.45 (12.96 to 13.94)	13.60 (13.11 to 14.09)	**1.00 (0.89 to 1.12)**	**0.77 (0.61 to 0.94)**	**-0.23 (-0.43 to -0.03)**
	≥8	8.65 (8.33 to 8.98)	10.27 (9.96 to 10.59)	11.26 (10.91 to 11.60)	13.42 (13.03 to 13.80)	15.04 (14.47 to 15.61)	15.35 (14.70 to 16.01)	15.47 (14.81 to 16.12)	**1.26 (1.12 to 1.41)**	**0.79 (0.57 to 1.01)**	**-0.48 (-0.74 to -0.21)**
Middle	<6	22.40 (21.70 to 23.10)	23.44 (22.91 to 23.97)	25.24 (24.75 to 25.73)	28.52 (27.98 to 29.05)	34.12 (33.08 to 35.16)	33.75 (32.80 to 34.69)	34.93 (34.00 to 35.85)	**1.94 (1.67 to 2.20)**	**2.35 (2.01 to 2.69)**	0.41 (-0.02 to 0.85)
	6–7	17.66 (17.22 to 18.10)	18.49 (18.15 to 18.83)	20.37 (20.03 to 20.71)	22.77 (22.40 to 23.13)	28.83 (28.07 to 29.59)	26.67 (25.98 to 27.36)	27.47 (26.78 to 28.16)	**1.64 (1.47 to 1.82)**	**1.73 (1.49 to 1.98)**	0.09 (-0.21 to 0.39)
	7–8	16.03 (15.63 to 16.44)	17.14 (16.82 to 17.46)	18.96 (18.63 to 19.29)	21.45 (21.10 to 21.80)	25.48 (24.79 to 26.18)	23.26 (22.62 to 23.90)	25.60 (24.93 to 26.27)	**1.67 (1.51 to 1.84)**	**1.27 (1.04 to 1.50)**	**-0.41 (-0.69 to -0.13)**
	≥8	17.26 (16.77 to 17.74)	18.59 (18.17 to 19.00)	20.74 (20.30 to 21.17)	23.34 (22.90 to 23.79)	27.34 (26.50 to 28.17)	26.00 (25.17 to 26.83)	28.14 (27.27 to 29.00)	**1.78 (1.58 to 1.98)**	**1.62 (1.33 to 1.92)**	-0.16 (-0.51 to 0.20)

Low	<6	45.82 (44.91 to 46.74)	47.88 (47.16 to 48.61)	49.58 (48.89 to 50.27)	50.83 (50.11 to 51.55)	54.25 (52.76 to 55.74)	52.39 (51.06 to 53.71)	55.49 (54.32 to 56.66)	**1.33 (0.95 to 1.71)**	**1.54 (1.07 to 2.01)**	0.21 (-0.39 to 0.82)
	6–7	42.14 (41.23 to 43.06)	43.20 (42.48 to 43.93)	44.88 (44.16 to 45.60)	46.43 (45.66 to 47.20)	48.88 (47.25 to 50.50)	47.14 (45.70 to 48.57)	49.49 (48.14 to 50.84)	**1.28 (0.90 to 1.67)**	**0.91 (0.39 to 1.42)**	-0.38 (-1.02 to 0.26)
	7–8	40.32 (39.43 to 41.21)	41.51 (40.77 to 42.25)	43.55 (42.82 to 44.28)	46.05 (45.29 to 46.82)	46.39 (44.72 to 48.05)	44.84 (43.34 to 46.34)	49.75 (48.31 to 51.19)	**1.79 (1.40 to 2.17)**	**1.01 (0.47 to 1.55)**	**-0.78 (-1.44 to -0.12)**
	≥8	42.41 (41.59 to 43.24)	44.76 (44.03 to 45.49)	45.06 (44.31 to 45.81)	49.02 (48.25 to 49.78)	48.53 (46.93 to 50.12)	46.13 (44.66 to 47.60)	50.93 (49.58 to 52.29)	**1.77 (1.40 to 2.14)**	0.31 (-0.20 to 0.83)	**-1.45 (-2.09 to -0.82)**
**Depression counseling**											
Yes	<6	31.09 (28.52 to 33.65)	31.33 (29.34 to 33.32)	32.04 (30.32 to 33.77)	32.78 (30.95 to 34.61)	30.52 (27.41 to 33.62)	33.08 (30.25 to 35.91)	31.22 (28.60 to 33.83)	0.35 (-0.63 to 1.32)	0.05 (-1.00 to 1.10)	-0.30 (-1.73 to 1.14)
	6–7	23.99 (21.39 to 26.60)	20.70 (18.75 to 22.64)	21.38 (19.68 to 23.09)	20.60 (18.82 to 22.38)	22.73 (19.74 to 25.73)	21.37 (18.66 to 24.09)	20.04 (17.54 to 22.54)	-0.67 (-1.58 to 0.24)	0.00 (-0.97 to 0.97)	0.67 (-0.66 to 2.00)
	7–8	20.05 (17.50 to 22.59)	18.71 (16.75 to 20.67)	20.05 (18.21 to 21.88)	19.07 (17.22 to 20.92)	17.44 (14.79 to 20.08)	17.13 (14.57 to 19.69)	18.04 (15.60 to 20.48)	0.00 (-0.95 to 0.95)	-0.63 (-1.61 to 0.34)	-0.64 (-2.00 to 0.73)
	≥8	21.02 (18.31 to 23.73)	23.28 (21.01 to 25.54)	22.15 (20.11 to 24.19)	21.40 (19.40 to 23.41)	20.79 (17.70 to 23.87)	17.24 (14.41 to 20.06)	21.67 (18.76 to 24.57)	-0.55 (-1.57 to 0.47)	-0.47 (-1.52 to 0.59)	0.08 (-1.39 to 1.55)
No	<6	24.84 (24.39 to 25.28)	26.81 (26.45 to 27.16)	28.22 (27.88 to 28.57)	31.57 (31.19 to 31.94)	33.62 (32.93 to 34.31)	34.85 (34.20 to 35.50)	36.55 (35.93 to 37.17)	**2.00 (1.82 to 2.18)**	**1.75 (1.52 to 1.98)**	-0.25 (-0.54 to 0.05)
	6–7	17.00 (16.72 to 17.28)	18.39 (18.17 to 18.62)	19.82 (19.59 to 20.05)	22.41 (22.15 to 22.66)	24.70 (24.23 to 25.17)	24.45 (23.99 to 24.91)	25.56 (25.11 to 26.02)	**1.60 (1.49 to 1.72)**	**1.13 (0.96 to 1.29)**	**-0.48 (-0.67 to -0.28)**

	7–8	14.85 (14.60 to 15.10)	16.61 (16.40 to 16.82)	18.01 (17.79 to 18.23)	20.42 (20.18 to 20.66)	20.38 (19.98 to 20.78)	20.70 (20.29 to 21.10)	22.39 (21.96 to 22.81)	**1.59 (1.49 to 1.70)**	**0.60 (0.46 to 0.75)**	**-0.99 (-1.17 to -0.81)**
	≥8	18.20 (17.89 to 18.51)	20.12 (19.84 to 20.39)	21.56 (21.27 to 21.85)	24.62 (24.31 to 24.93)	23.58 (23.08 to 24.08)	24.57 (24.04 to 25.10)	26.60 (26.05 to 27.15)	**1.79 (1.66 to 1.92)**	**0.63 (0.44 to 0.82)**	**-1.16 (-1.39 to -0.93)**
**Stress counseling**											
Yes	<6	31.21 (27.93 to 34.49)	32.69 (30.05 to 35.33)	37.11 (34.72 to 39.50)	34.83 (32.46 to 37.19)	33.71 (29.71 to 37.71)	35.87 (32.15 to 39.59)	37.77 (34.34 to 41.21)	0.74 (-0.56 to 2.03)	1.00 (-0.39 to 2.38)	0.26 (-1.63 to 2.15)
	6–7	24.36 (20.99 to 27.73)	24.66 (21.84 to 27.47)	25.67 (23.21 to 28.13)	24.44 (21.89 to 27.00)	25.18 (21.02 to 29.33)	25.78 (21.73 to 29.83)	25.35 (21.72 to 28.98)	-0.13 (-1.43 to 1.16)	0.38 (-1.01 to 1.77)	0.51 (-1.39 to 2.41)
	7–8	21.46 (18.13 to 24.79)	20.09 (17.27 to 22.90)	23.49 (20.91 to 26.06)	24.29 (21.70 to 26.87)	20.16 (16.49 to 23.83)	21.08 (17.62 to 24.53)	25.05 (21.54 to 28.57)	1.02 (-0.29 to 2.34)	-0.06 (-1.49 to 1.37)	-1.08 (-3.02 to 0.86)
	≥8	22.19 (18.98 to 25.40)	25.80 (22.85 to 28.74)	24.91 (22.37 to 27.45)	25.80 (23.25 to 28.35)	25.50 (21.42 to 29.58)	22.28 (18.67 to 25.89)	27.86 (24.19 to 31.53)	0.59 (-0.67 to 1.85)	-0.01 (-1.36 to 1.34)	-0.60 (-2.45 to 1.25)
No	<6	24.91 (24.46 to 25.35)	26.84 (26.49 to 27.20)	28.17 (27.83 to 28.51)	31.53 (31.16 to 31.90)	33.47 (32.78 to 34.15)	34.72 (34.08 to 35.37)	36.22 (35.61 to 36.84)	**1.96 (1.79 to 2.14)**	**1.68 (1.44 to 1.91)**	-0.29 (-0.58 to 0.00)
	6–7	17.04 (16.77 to 17.32)	18.38 (18.15 to 18.61)	19.80 (19.57 to 20.03)	22.35 (22.09 to 22.61)	24.65 (24.18 to 25.11)	24.36 (23.90 to 24.81)	25.42 (24.97 to 25.88)	**1.58 (1.47 to 1.69)**	**1.10 (0.94 to 1.26)**	**-0.48 (-0.68 to -0.28)**
	7–8	14.87 (14.62 to 15.12)	16.61 (16.40 to 16.82)	18.00 (17.78 to 18.21)	20.37 (20.13 to 20.61)	20.32 (19.92 to 20.72)	20.62 (20.22 to 21.02)	22.26 (21.84 to 22.68)	**1.57 (1.47 to 1.68)**	**0.58 (0.43 to 0.72)**	**-1.00 (-1.17 to -0.82)**
	≥8	18.20 (17.89 to 18.50)	20.11 (19.83 to 20.38)	21.53 (21.24 to 21.81)	24.54 (24.23 to 24.84)	23.48 (22.99 to 23.97)	24.42 (23.89 to 24.94)	26.43 (25.88 to 26.98)	**1.76 (1.63 to 1.89)**	**0.60 (0.41 to 0.79)**	**-1.16 (-1.39 to -0.93)**

**Level of education**											
High school or lower education	<6	40.13 (39.42 to 40.84)	44.16 (43.57 to 44.75)	47.85 (47.28 to 48.42)	50.99 (50.39 to 51.59)	52.06 (50.99 to 53.13)	53.56 (52.55 to 54.58)	55.37 (54.42 to 56.33)	**3.63 (3.32 to 3.93)**	**1.43 (1.06 to 1.81)**	**-2.19 (-2.68 to -1.71)**
	6–7	34.92 (34.29 to 35.54)	39.18 (38.64 to 39.73)	43.54 (42.99 to 44.10)	47.46 (46.86 to 48.05)	47.43 (46.40 to 48.46)	48.01 (46.95 to 49.07)	50.46 (49.43 to 51.48)	**4.27 (3.99 to 4.55)**	**0.97 (0.58 to 1.36)**	**-3.30 (-3.78 to -2.82)**
	7–8	33.22 (32.61 to 33.83)	38.20 (37.67 to 38.73)	42.36 (41.81 to 42.91)	46.81 (46.22 to 47.39)	46.24 (45.22 to 47.26)	47.75 (46.68 to 48.83)	50.39 (49.31 to 51.47)	**4.56 (4.29 to 4.84)**	**1.12 (0.73 to 1.52)**	**-3.44 (-3.92 to -2.96)**
	≥8	36.62 (36.01 to 37.24)	41.20 (40.64 to 41.75)	45.03 (44.44 to 45.62)	49.97 (49.37 to 50.57)	48.92 (47.89 to 49.95)	51.18 (50.07 to 52.29)	51.96 (50.89 to 53.03)	**4.46 (4.17 to 4.74)**	**0.84 (0.43 to 1.24)**	**-3.62 (-4.11 to -3.13)**
College or higher education	<6	14.14 (13.66 to 14.62)	15.83 (15.44 to 16.21)	17.28 (16.91 to 17.64)	20.18 (19.77 to 20.60)	21.85 (21.07 to 22.63)	23.51 (22.78 to 24.24)	24.92 (24.21 to 25.63)	**1.86 (1.67 to 2.06)**	**1.60 (1.34 to 1.85)**	-0.27 (-0.59 to 0.05)
	6–7	10.74 (10.47 to 11.01)	12.03 (11.81 to 12.25)	13.59 (13.37 to 13.82)	15.46 (15.20 to 15.71)	17.71 (17.23 to 18.19)	18.12 (17.65 to 18.59)	18.86 (18.39 to 19.32)	**1.47 (1.36 to 1.59)**	**1.17 (1.01 to 1.33)**	**-0.30 (-0.50 to -0.11)**
	7–8	9.21 (8.98 to 9.45)	10.61 (10.41 to 10.82)	12.01 (11.81 to 12.22)	13.78 (13.55 to 14.01)	14.28 (13.88 to 14.67)	15.04 (14.64 to 15.44)	16.23 (15.81 to 16.65)	**1.39 (1.29 to 1.49)**	**0.74 (0.60 to 0.88)**	**-0.65 (-0.82 to -0.48)**
	≥8	9.70 (9.41 to 9.99)	11.18 (10.92 to 11.44)	12.41 (12.13 to 12.68)	14.60 (14.30 to 14.90)	14.60 (14.11 to 15.09)	15.55 (15.03 to 16.07)	17.70 (17.15 to 18.26)	**1.40 (1.28 to 1.53)**	**0.88 (0.69 to 1.06)**	**-0.53 (-0.75 to -0.30)**
**Household income**											
Lowest and second quartile	<6	16.91 (16.26 to 17.55)	17.85 (17.38 to 18.31)	18.18 (17.74 to 18.63)	21.09 (20.62 to 21.56)	22.61 (21.72 to 23.50)	24.65 (23.80 to 25.49)	25.58 (24.78 to 26.37)	**1.33 (1.09 to 1.57)**	**1.45 (1.16 to 1.74)**	0.12 (-0.26 to 0.50)

	6–7	12.42 (12.07 to 12.77)	13.13 (12.87 to 13.40)	14.14 (13.87 to 14.41)	16.11 (15.83 to 16.40)	18.04 (17.50 to 18.58)	18.30 (17.78 to 18.82)	19.21 (18.70 to 19.73)	**1.18 (1.04 to 1.31)**	**1.01 (0.83 to 1.19)**	-0.16 (-0.39 to 0.06)
	7–8	10.83 (10.51 to 11.14)	11.45 (11.20 to 11.69)	12.43 (12.18 to 12.68)	14.07 (13.81 to 14.33)	14.67 (14.23 to 15.11)	14.90 (14.46 to 15.34)	16.40 (15.95 to 16.85)	**1.02 (0.90 to 1.14)**	**0.65 (0.49 to 0.81)**	**-0.37 (-0.57 to -0.17)**
	≥8	12.90 (12.49 to 13.31)	12.99 (12.66 to 13.32)	13.75 (13.39 to 14.11)	15.31 (14.95 to 15.66)	15.23 (14.66 to 15.81)	15.92 (15.32 to 16.51)	18.30 (17.67 to 18.92)	**0.74 (0.58 to 0.90)**	**0.79 (0.57 to 1.00)**	0.05 (-0.22 to 0.31)
Third and highest quartile	<6	30.28 (29.70 to 30.85)	34.79 (34.29 to 35.30)	36.76 (36.30 to 37.23)	42.52 (41.98 to 43.05)	43.76 (42.82 to 44.70)	44.48 (43.58 to 45.37)	47.94 (47.08 to 48.80)	**3.73 (3.48 to 3.98)**	**1.90 (1.56 to 2.24)**	**-1.83 (-2.25 to -1.41)**
	6–7	21.87 (21.46 to 22.28)	25.83 (25.45 to 26.21)	27.53 (27.16 to 27.90)	33.73 (33.26 to 34.19)	35.88 (35.06 to 36.70)	35.71 (34.90 to 36.52)	38.23 (37.42 to 39.05)	**3.41 (3.22 to 3.60)**	**1.68 (1.38 to 1.98)**	**-1.73 (-2.09 to -1.38)**
	7–8	19.05 (18.68 to 19.42)	24.04 (23.68 to 24.39)	25.45 (25.10 to 25.81)	32.37 (31.93 to 32.81)	31.30 (30.55 to 32.04)	32.24 (31.47 to 33.00)	36.00 (35.17 to 36.83)	**3.72 (3.55 to 3.89)**	**1.06 (0.77 to 1.35)**	**-2.66 (-3.00 to -2.32)**
	≥8	22.29 (21.87 to 22.72)	27.36 (26.94 to 27.77)	28.91 (28.49 to 29.33)	36.29 (35.80 to 36.78)	34.61 (33.81 to 35.41)	36.10 (35.22 to 36.97)	38.85 (37.96 to 39.73)	**3.99 (3.79 to 4.19)**	**0.90 (0.58 to 1.22)**	**-3.09 (-3.47 to -2.72)**
**Smoking status**											
Smoker	<6	16.32 (15.54 to 17.10)	17.82 (17.17 to 18.47)	19.69 (19.02 to 20.35)	22.35 (21.57 to 23.12)	25.25 (23.74 to 26.75)	25.62 (24.18 to 27.06)	28.17 (26.71 to 29.63)	**1.99 (1.65 to 2.32)**	**1.87 (1.36 to 2.38)**	-0.12 (-0.73 to 0.49)
	6–7	12.52 (12.04 to 12.99)	13.63 (13.21 to 14.04)	15.86 (15.41 to 16.30)	18.01 (17.49 to 18.54)	21.27 (20.20 to 22.34)	21.24 (20.20 to 22.29)	22.18 (21.18 to 23.19)	**1.83 (1.61 to 2.04)**	**1.56 (1.20 to 1.91)**	-0.27 (-0.68 to 0.15)
	7–8	11.80 (11.36 to 12.24)	13.83 (13.41 to 14.24)	15.47 (15.01 to 15.92)	16.92 (16.41 to 17.43)	17.53 (16.64 to 18.42)	17.61 (16.70 to 18.53)	19.72 (18.76 to 20.67)	**1.56 (1.35 to 1.76)**	**0.77 (0.44 to 1.09)**	**-0.79 (-1.17 to -0.41)**

	≥8	14.80 (14.24 to 15.36)	16.99 (16.43 to 17.54)	18.79 (18.17 to 19.41)	19.99 (19.31 to 20.66)	19.99 (18.90 to 21.07)	20.99 (19.76 to 22.21)	22.59 (21.42 to 23.75)	**1.63 (1.37 to 1.90)**	**0.86 (0.45 to 1.28)**	**-0.77 (-1.26 to -0.28)**
Ex-smoker	<6	32.32 (31.07 to 33.58)	34.20 (33.26 to 35.13)	35.67 (34.81 to 36.54)	38.27 (37.36 to 39.18)	37.07 (35.43 to 38.71)	39.05 (37.51 to 40.59)	39.78 (38.41 to 41.16)	**1.84 (1.35 to 2.32)**	**0.64 (0.09 to 1.18)**	**-1.20 (-1.93 to -0.47)**
	6–7	25.32 (24.51 to 26.14)	27.00 (26.37 to 27.62)	28.51 (27.91 to 29.11)	30.77 (30.13 to 31.40)	31.89 (30.71 to 33.06)	31.08 (29.99 to 32.18)	32.88 (31.85 to 33.92)	**1.69 (1.37 to 2.01)**	**0.68 (0.29 to 1.07)**	**-1.01 (-1.51 to -0.51)**
	7–8	24.20 (23.42 to 24.97)	26.23 (25.63 to 26.84)	28.36 (27.77 to 28.95)	30.83 (30.21 to 31.45)	28.13 (27.09 to 29.18)	30.48 (29.43 to 31.53)	31.45 (30.44 to 32.46)	**2.09 (1.78 to 2.39)**	0.18 (-0.19 to 0.55)	**-1.91 (-2.39 to -1.43)**
	≥8	30.09 (29.17 to 31.02)	32.60 (31.83 to 33.37)	34.85 (34.08 to 35.62)	37.95 (37.18 to 38.72)	35.47 (34.18 to 36.77)	37.05 (35.70 to 38.40)	37.46 (36.21 to 38.70)	**2.44 (2.06 to 2.81)**	-0.17 (-0.64 to 0.30)	**-2.60 (-3.21 to -2.00)**
Non-smoker	<6	26.54 (25.98 to 27.10)	28.16 (27.72 to 28.60)	29.15 (28.73 to 29.57)	32.36 (31.91 to 32.81)	34.49 (33.68 to 35.30)	35.74 (34.98 to 36.50)	37.11 (36.36 to 37.86)	**1.61 (1.39 to 1.83)**	**1.70 (1.42 to 1.98)**	0.09 (-0.27 to 0.44)
	6–7	16.99 (16.64 to 17.34)	17.92 (17.64 to 18.20)	18.80 (18.52 to 19.08)	21.22 (20.91 to 21.53)	23.43 (22.89 to 23.98)	23.16 (22.61 to 23.71)	23.64 (23.09 to 24.18)	**1.13 (0.99 to 1.26)**	**0.90 (0.71 to 1.09)**	-0.22 (-0.46 to 0.01)
	7–8	14.07 (13.77 to 14.36)	15.19 (14.94 to 15.44)	16.04 (15.79 to 16.29)	18.45 (18.17 to 18.72)	18.94 (18.47 to 19.40)	18.70 (18.23 to 19.17)	19.92 (19.43 to 20.41)	**1.16 (1.04 to 1.28)**	**0.50 (0.33 to 0.67)**	**-0.66 (-0.87 to -0.46)**
	≥8	16.87 (16.51 to 17.23)	18.12 (17.80 to 18.44)	18.85 (18.52 to 19.18)	21.94 (21.58 to 22.30)	21.19 (20.63 to 21.76)	21.64 (21.04 to 22.24)	23.64 (22.99 to 24.28)	**1.24 (1.09 to 1.39)**	**0.51 (0.30 to 0.73)**	**-0.73 (-0.99 to -0.47)**
**Alcohol consumption**											
Non-drinker and 1day/month	<6	28.92 (28.34 to 29.51)	31.52 (31.04 to 31.99)	33.27 (32.81 to 33.73)	36.12 (35.64 to 36.60)	36.67 (35.86 to 37.48)	37.55 (36.77 to 38.32)	39.40 (38.66 to 40.14)	**2.09 (1.85 to 2.33)**	**1.08 (0.79 to 1.37)**	**-1.01 (-1.38 to -0.63)**

	6–7	19.77 (19.38 to 20.16)	21.35 (21.02 to 21.68)	22.91 (22.58 to 23.24)	25.71 (25.34 to 26.07)	26.94 (26.34 to 27.53)	26.76 (26.17 to 27.36)	27.45 (26.86 to 28.04)	**1.70 (1.54 to 1.86)**	**0.71 (0.49 to 0.92)**	**-0.99 (-1.26 to -0.72)**
	7–8	16.78 (16.44 to 17.12)	18.92 (18.62 to 19.22)	20.46 (20.15 to 20.77)	23.62 (23.28 to 23.95)	22.52 (22.00 to 23.05)	22.48 (21.96 to 23.00)	24.64 (24.08 to 25.21)	**1.94 (1.80 to 2.09)**	**0.24 (0.04 to 0.43)**	**-1.71 (-1.95 to -1.46)**
	≥8	20.33 (19.93 to 20.72)	22.62 (22.25 to 22.98)	24.44 (24.05 to 24.83)	28.34 (27.93 to 28.75)	25.91 (25.28 to 26.54)	26.69 (26.02 to 27.35)	29.15 (28.46 to 29.83)	**2.24 (2.07 to 2.41)**	0.16 (-0.08 to 0.40)	**-2.08 (-2.38 to -1.78)**
2–9 days/month	<6	17.08 (16.39 to 17.77)	18.41 (17.87 to 18.94)	19.44 (18.94 to 19.94)	22.63 (22.04 to 23.22)	24.69 (23.47 to 25.91)	26.74 (25.55 to 27.92)	28.38 (27.27 to 29.50)	**1.72 (1.45 to 2.00)**	**2.02 (1.63 to 2.42)**	0.30 (-0.18 to 0.78)
	6–7	12.60 (12.21 to 12.98)	13.76 (13.45 to 14.07)	15.10 (14.79 to 15.41)	16.88 (16.52 to 17.24)	19.41 (18.67 to 20.15)	19.35 (18.61 to 20.08)	21.08 (20.37 to 21.79)	**1.36 (1.21 to 1.52)**	**1.36 (1.12 to 1.60)**	-0.01 (-0.30 to 0.28)
	7–8	11.24 (10.90 to 11.58)	12.50 (12.21 to 12.79)	13.66 (13.36 to 13.95)	14.87 (14.54 to 15.19)	15.64 (15.05 to 16.24)	16.33 (15.70 to 16.95)	17.41 (16.80 to 18.02)	**1.07 (0.93 to 1.21)**	**0.83 (0.62 to 1.04)**	-0.24 (-0.50 to 0.01)
	≥8	12.88 (12.43 to 13.32)	13.98 (13.58 to 14.38)	14.78 (14.37 to 15.19)	16.08 (15.64 to 16.51)	16.77 (16.00 to 17.54)	17.26 (16.44 to 18.08)	19.09 (18.24 to 19.93)	**0.93 (0.74 to 1.11)**	**0.98 (0.70 to 1.25)**	0.05 (-0.28 to 0.38)
Over 10 days/month	<6	26.53 (25.00 to 28.06)	28.52 (27.31 to 29.73)	31.20 (30.04 to 32.36)	32.74 (31.45 to 34.03)	35.91 (33.21 to 38.61)	35.82 (33.27 to 38.37)	36.62 (34.15 to 39.09)	**2.00 (1.37 to 2.63)**	**1.60 (0.72 to 2.48)**	-0.40 (-1.48 to 0.69)
	6–7	22.63 (21.47 to 23.80)	24.93 (24.00 to 25.87)	27.08 (26.16 to 28.01)	28.97 (27.94 to 29.99)	32.87 (30.58 to 35.17)	28.44 (26.29 to 30.59)	32.06 (29.98 to 34.13)	**1.99 (1.51 to 2.47)**	**0.90 (0.18 to 1.62)**	**-1.10 (-1.96 to -0.23)**
	7–8	21.98 (20.81 to 23.15)	24.74 (23.79 to 25.69)	27.89 (26.92 to 28.85)	29.28 (28.25 to 30.31)	28.70 (26.64 to 30.75)	28.02 (25.98 to 30.06)	31.56 (29.52 to 33.60)	**2.36 (1.88 to 2.85)**	0.47 (-0.24 to 1.18)	**-1.89 (-2.75 to -1.04)**

	≥8	26.47 (25.22 to 27.73)	29.74 (28.62 to 30.85)	31.73 (30.61 to 32.84)	34.03 (32.85 to 35.22)	32.69 (30.42 to 34.96)	34.62 (32.11 to 37.13)	35.84 (33.55 to 38.13)	**2.27 (1.71 to 2.82)**	0.45 (-0.37 to 1.28)	**-1.81 (-2.81 to -0.82)**

Abbreviations: BMI, body mass index; CI, confidence interval; KCHS, Korea Community Health Survey.

β values indicate the average annual change (%) in the weighted prevalence, multiplied by 100 for interpretability.

The values in bold font represent significant variance ($p<0.05$).

[a] According to the Asian-Pacific guidelines, BMI is divided into four groups: underweight (<18.5 kg/ m^2), normal (18.5–22.9 kg/ m^2), overweight (23.0–24.9 kg/ m^2), and obese (≥25.0 kg/ m^2).

Table S3. National trends in sleep duration among individuals with diabetes and β-coefficients of odds ratios before and during the COVID–19 pandemic (weighted % [95% CI]).

Variables	Sleep duration (hours/day)	Pre-pandemic				During the pandemic			Trends in the pre-pandemic era, β (95% CI)	Trends in the pandemic era, β (95% CI)	β_{diff} between 2009-2019 and 2019-2022 (95% CI)
		2009-2010	2011-2013	2014-2016	2017-2019	2020	2021	2022			
**Overall**	<6	9.04 (8.74 to 9.33)	10.08 (9.85 to 10.32)	11.15 (10.91 to 11.38)	12.67 (12.41 to 12.92)	14.37 (13.87 to 14.86)	15.00 (14.53 to 15.46)	15.68 (15.22 to 16.14)	**1.13 (1.01 to 1.25)**	**1.04 (0.88 to 1.20)**	-0.09 (-0.29 to 0.11)
	6–7	6.09 (5.92 to 6.26)	6.60 (6.46 to 6.74)	7.37 (7.23 to 7.51)	8.76 (8.59 to 8.93)	10.05 (9.72 to 10.37)	10.38 (10.07 to 10.69)	10.85 (10.54 to 11.16)	**0.80 (0.73 to 0.87)**	**0.73 (0.63 to 0.84)**	-0.07 (-0.19 to 0.06)
	7–8	5.50 (5.35 to 5.65)	6.25 (6.12 to 6.39)	7.17 (7.03 to 7.31)	8.20 (8.04 to 8.35)	8.43 (8.16 to 8.69)	9.00 (8.72 to 9.27)	9.98 (9.69 to 10.28)	**0.80 (0.74 to 0.87)**	**0.54 (0.44 to 0.64)**	**-0.26 (-0.38 to -0.15)**
	≥8	8.87 (8.63 to 9.10)	10.07 (9.85 to 10.29)	11.73 (11.49 to 11.97)	13.46 (13.20 to 13.73)	13.52 (13.09 to 13.95)	14.20 (13.74 to 14.65)	16.15 (15.66 to 16.63)	**1.02 (0.93 to 1.11)**	**0.62 (0.49 to 0.75)**	**-0.40 (-0.56 to -0.24)**
**Sex**											
Male	<6	8.52 (8.08 to 8.97)	9.55 (9.20 to 9.91)	11.09 (10.73 to 11.45)	12.93 (12.51 to 13.34)	15.13 (14.33 to 15.93)	15.89 (15.13 to 16.65)	16.26 (15.52 to 17.01)	**1.42 (1.24 to 1.59)**	**1.20 (0.94 to 1.46)**	-0.21 (-0.53 to 0.10)
	6–7	6.48 (6.23 to 6.72)	7.05 (6.84 to 7.26)	7.92 (7.71 to 8.13)	9.41 (9.16 to 9.66)	11.04 (10.54 to 11.54)	11.36 (10.89 to 11.83)	12.19 (11.71 to 12.66)	**0.89 (0.78 to 0.99)**	**0.95 (0.79 to 1.11)**	0.07 (-0.12 to 0.26)

	7–8	6.40 (6.16 to 6.63)	7.29 (7.08 to 7.50)	8.53 (8.31 to 8.75)	9.44 (9.19 to 9.68)	9.65 (9.24 to 10.06)	10.59 (10.16 to 11.02)	11.27 (10.83 to 11.72)	**0.93 (0.83 to 1.02)**	**0.61 (0.46 to 0.76)**	**-0.31 (-0.49 to -0.14)**
	≥8	10.81 (10.42 to 11.19)	12.72 (12.35 to 13.09)	15.04 (14.64 to 15.44)	17.11 (16.68 to 17.55)	16.46 (15.79 to 17.13)	18.12 (17.36 to 18.87)	19.65 (18.90 to 20.40)	**1.35 (1.21 to 1.49)**	**0.66 (0.45 to 0.86)**	**-0.69 (-0.94 to -0.44)**
Female	<6	9.43 (9.05 to 9.82)	10.45 (10.15 to 10.76)	11.18 (10.89 to 11.48)	12.50 (12.18 to 12.82)	13.89 (13.28 to 14.50)	14.43 (13.86 to 15.01)	15.31 (14.74 to 15.87)	**0.88 (0.73 to 1.03)**	**0.93 (0.73 to 1.13)**	0.04 (-0.21 to 0.30)
	6–7	5.72 (5.49 to 5.95)	6.18 (5.99 to 6.36)	6.85 (6.66 to 7.04)	8.18 (7.96 to 8.40)	9.18 (8.78 to 9.59)	9.51 (9.10 to 9.92)	9.64 (9.25 to 10.04)	**0.71 (0.62 to 0.80)**	**0.51 (0.38 to 0.65)**	**-0.20 (-0.36 to -0.03)**
	7–8	4.70 (4.51 to 4.89)	5.37 (5.20 to 5.53)	5.99 (5.82 to 6.16)	7.13 (6.93 to 7.33)	7.36 (7.02 to 7.69)	7.58 (7.24 to 7.93)	8.85 (8.47 to 9.22)	**0.68 (0.60 to 0.76)**	**0.47 (0.35 to 0.60)**	**-0.20 (-0.35 to -0.06)**
	≥8	7.44 (7.15 to 7.72)	8.24 (7.98 to 8.49)	9.37 (9.09 to 9.65)	10.80 (10.49 to 11.11)	11.24 (10.71 to 11.76)	11.16 (10.64 to 11.68)	13.27 (12.67 to 13.86)	**0.72 (0.61 to 0.82)**	**0.55 (0.39 to 0.71)**	-0.17 (-0.36 to 0.02)
**Age, years**											
19–30	<6	0.23 (0.10 to 0.36)	0.40 (0.27 to 0.54)	0.39 (0.24 to 0.54)	0.44 (0.27 to 0.61)	0.51 (0.18 to 0.85)	0.58 (0.23 to 0.92)	1.29 (0.69 to 1.89)	0.06 (-0.01 to 0.14)	**0.24 (0.05 to 0.42)**	0.17 (-0.03 to 0.37)
	6–7	0.31 (0.21 to 0.41)	0.33 (0.23 to 0.42)	0.26 (0.19 to 0.33)	0.42 (0.30 to 0.53)	0.43 (0.22 to 0.63)	0.51 (0.30 to 0.71)	0.65 (0.41 to 0.88)	0.03 (-0.03 to 0.09)	0.06 (-0.03 to 0.15)	0.03 (-0.07 to 0.14)
	7–8	0.26 (0.17 to 0.34)	0.30 (0.22 to 0.37)	0.36 (0.28 to 0.44)	0.36 (0.28 to 0.45)	0.44 (0.27 to 0.60)	0.35 (0.20 to 0.50)	0.44 (0.29 to 0.60)	**0.04 (0.01 to 0.08)**	0.03 (-0.03 to 0.09)	-0.01 (-0.08 to 0.06)

	≥8	0.37 (0.27 to 0.47)	0.35 (0.26 to 0.45)	0.47 (0.36 to 0.58)	0.38 (0.29 to 0.48)	0.49 (0.31 to 0.66)	0.55 (0.36 to 0.75)	0.44 (0.25 to 0.62)	0.02 (-0.02 to 0.07)	0.04 (-0.03 to 0.11)	0.01 (-0.07 to 0.10)
31–40	<6	1.56 (1.22 to 1.89)	1.72 (1.43 to 2.02)	1.59 (1.34 to 1.84)	2.26 (1.89 to 2.62)	3.30 (2.41 to 4.18)	2.95 (2.22 to 3.67)	2.15 (1.48 to 2.83)	**0.22 (0.05 to 0.38)**	0.03 (-0.21 to 0.27)	-0.19 (-0.48 to 0.11)
	6–7	0.98 (0.83 to 1.13)	1.03 (0.89 to 1.16)	1.33 (1.18 to 1.48)	1.45 (1.27 to 1.63)	1.68 (1.30 to 2.05)	2.06 (1.64 to 2.48)	1.90 (1.50 to 2.30)	**0.18 (0.10 to 0.25)**	**0.21 (0.07 to 0.35)**	0.03 (-0.13 to 0.19)
	7–8	0.96 (0.82 to 1.09)	1.09 (0.96 to 1.22)	1.35 (1.21 to 1.50)	1.29 (1.13 to 1.46)	1.27 (0.99 to 1.55)	1.69 (1.36 to 2.01)	2.05 (1.70 to 2.41)	**0.12 (0.05 to 0.18)**	**0.26 (0.14 to 0.38)**	**0.14 (0.00 to 0.28)**
	≥8	1.30 (1.11 to 1.49)	1.35 (1.17 to 1.54)	1.52 (1.31 to 1.72)	1.56 (1.34 to 1.78)	1.95 (1.46 to 2.43)	1.67 (1.25 to 2.08)	2.42 (1.91 to 2.93)	0.09 (0.00 to 0.19)	**0.23 (0.06 to 0.40)**	0.14 (-0.06 to 0.33)
41–50	<6	4.54 (4.06 to 5.01)	4.92 (4.51 to 5.33)	4.84 (4.46 to 5.21)	5.60 (5.14 to 6.07)	6.15 (5.22 to 7.08)	5.52 (4.67 to 6.37)	7.13 (6.19 to 8.07)	**0.34 (0.12 to 0.56)**	**0.35 (0.03 to 0.68)**	0.02 (-0.38 to 0.41)
	6–7	3.74 (3.47 to 4.01)	3.46 (3.25 to 3.67)	3.88 (3.66 to 4.10)	4.30 (4.04 to 4.57)	4.31 (3.81 to 4.82)	4.88 (4.36 to 5.41)	5.43 (4.89 to 5.96)	**0.25 (0.13 to 0.38)**	**0.37 (0.17 to 0.57)**	0.12 (-0.12 to 0.35)
	7–8	3.46 (3.21 to 3.71)	3.46 (3.25 to 3.67)	3.64 (3.42 to 3.86)	3.93 (3.68 to 4.18)	4.45 (4.00 to 4.91)	4.48 (4.01 to 4.95)	4.57 (4.08 to 5.05)	**0.19 (0.07 to 0.31)**	**0.21 (0.03 to 0.38)**	0.02 (-0.20 to 0.23)
	≥8	4.98 (4.57 to 5.39)	4.56 (4.22 to 4.90)	4.81 (4.43 to 5.18)	5.11 (4.69 to 5.53)	5.63 (4.87 to 6.38)	5.55 (4.77 to 6.34)	6.15 (5.32 to 6.99)	0.06 (-0.12 to 0.24)	0.25 (-0.02 to 0.52)	0.19 (-0.13 to 0.51)
51–60	<6	10.11 (9.39 to 10.84)	10.25 (9.73 to 10.77)	10.80 (10.31 to 11.29)	11.55 (11.00 to 12.09)	11.74 (10.71 to 12.77)	12.75 (11.76 to 13.74)	12.55 (11.63 to 13.48)	**0.61 (0.32 to 0.90)**	**0.36 (0.00 to 0.72)**	-0.25 (-0.71 to 0.22)

	6–7	9.29 (8.79 to 9.78)	9.00 (8.64 to 9.36)	9.05 (8.71 to 9.39)	10.01 (9.62 to 10.39)	10.05 (9.39 to 10.71)	10.85 (10.17 to 11.53)	10.75 (10.09 to 11.42)	**0.31 (0.10 to 0.51)**	**0.30 (0.04 to 0.55)**	-0.01 (-0.34 to 0.32)
	7–8	9.23 (8.76 to 9.70)	9.05 (8.68 to 9.42)	9.30 (8.94 to 9.65)	9.51 (9.13 to 9.88)	9.46 (8.84 to 10.08)	9.82 (9.14 to 10.49)	10.76 (10.07 to 11.45)	**0.24 (0.04 to 0.44)**	**0.34 (0.08 to 0.61)**	0.11 (-0.22 to 0.43)
	≥8	14.00 (13.24 to 14.75)	14.49 (13.86 to 15.13)	15.77 (15.08 to 16.45)	14.81 (14.13 to 15.50)	16.41 (15.23 to 17.60)	15.98 (14.77 to 17.19)	16.01 (14.79 to 17.24)	**0.32 (0.05 to 0.60)**	**0.40 (0.03 to 0.78)**	0.08 (-0.38 to 0.55)
61–70	<6	17.50 (16.60 to 18.39)	18.22 (17.53 to 18.92)	18.99 (18.33 to 19.66)	18.86 (18.20 to 19.52)	19.98 (18.80 to 21.15)	19.35 (18.25 to 20.44)	19.97 (18.97 to 20.97)	**0.49 (0.13 to 0.84)**	0.29 (-0.11 to 0.69)	-0.19 (-0.73 to 0.34)
	6–7	15.71 (14.99 to 16.42)	17.46 (16.85 to 18.08)	17.73 (17.15 to 18.31)	17.40 (16.84 to 17.96)	17.65 (16.69 to 18.61)	17.97 (17.07 to 18.87)	17.58 (16.74 to 18.42)	**0.41 (0.11 to 0.70)**	0.11 (-0.23 to 0.44)	-0.30 (-0.75 to 0.14)
	7–8	16.32 (15.60 to 17.05)	17.60 (17.00 to 18.21)	18.54 (17.95 to 19.14)	17.74 (17.19 to 18.29)	18.08 (17.16 to 19.00)	18.32 (17.39 to 19.24)	19.06 (18.16 to 19.96)	**0.40 (0.11 to 0.70)**	**0.42 (0.07 to 0.77)**	0.01 (-0.44 to 0.47)
	≥8	28.17 (27.07 to 29.26)	30.08 (29.09 to 31.07)	31.55 (30.54 to 32.56)	31.37 (30.39 to 32.34)	29.40 (27.89 to 30.91)	30.70 (29.18 to 32.23)	32.28 (30.81 to 33.76)	**0.85 (0.49 to 1.21)**	0.33 (-0.12 to 0.78)	-0.52 (-1.09 to 0.06)
>70	<6	17.16 (16.26 to 18.05)	20.00 (19.30 to 20.70)	23.16 (22.49 to 23.83)	23.13 (22.48 to 23.79)	24.63 (23.51 to 25.76)	25.84 (24.81 to 26.87)	26.07 (25.04 to 27.10)	**1.91 (1.56 to 2.26)**	**1.09 (0.69 to 1.49)**	**-0.82 (-1.35 to -0.29)**

	6–7	17.84 (16.91 to 18.76)	19.43 (18.70 to 20.15)	21.99 (21.29 to 22.68)	22.89 (22.21 to 23.57)	23.85 (22.72 to 24.97)	23.76 (22.67 to 24.86)	24.92 (23.87 to 25.96)	**1.68 (1.31 to 2.04)**	**0.69 (0.28 to 1.10)**	**-0.99 (-1.54 to -0.44)**
	7–8	17.80 (16.86 to 18.75)	20.06 (19.31 to 20.81)	22.30 (21.58 to 23.01)	23.40 (22.72 to 24.08)	22.56 (21.47 to 23.65)	25.28 (24.15 to 26.41)	26.32 (25.18 to 27.46)	**1.84 (1.47 to 2.21)**	**1.01 (0.58 to 1.45)**	**-0.83 (-1.40 to -0.26)**
	≥8	30.36 (29.16 to 31.57)	34.66 (33.65 to 35.67)	39.74 (38.74 to 40.73)	42.63 (41.68 to 43.59)	41.79 (40.23 to 43.34)	43.19 (41.59 to 44.80)	45.29 (43.75 to 46.83)	**2.17 (1.83 to 2.51)**	**0.77 (0.35 to 1.19)**	**-1.40 (-1.93 to -0.86)**
**Region of residence**											
Urban	<6	8.65 (8.30 to 8.99)	9.57 (9.30 to 9.84)	10.55 (10.29 to 10.81)	12.12 (11.82 to 12.41)	13.97 (13.40 to 14.54)	14.60 (14.07 to 15.13)	15.18 (14.65 to 15.71)	**1.09 (0.95 to 1.22)**	**1.04 (0.86 to 1.23)**	-0.04 (-0.27 to 0.19)
	6–7	5.73 (5.53 to 5.92)	6.11 (5.95 to 6.26)	6.86 (6.70 to 7.03)	8.20 (8.01 to 8.38)	9.48 (9.11 to 9.84)	9.88 (9.53 to 10.23)	10.33 (9.98 to 10.67)	**0.74 (0.66 to 0.82)**	**0.73 (0.61 to 0.85)**	-0.01 (-0.15 to 0.13)
	7–8	5.09 (4.91 to 5.27)	5.75 (5.59 to 5.90)	6.66 (6.50 to 6.82)	7.61 (7.43 to 7.79)	7.86 (7.55 to 8.16)	8.33 (8.03 to 8.64)	9.54 (9.21 to 9.87)	**0.76 (0.68 to 0.83)**	**0.55 (0.44 to 0.66)**	**-0.21 (-0.34 to -0.08)**
	≥8	8.10 (7.82 to 8.38)	9.11 (8.86 to 9.37)	10.75 (10.47 to 11.03)	12.31 (12.01 to 12.61)	12.40 (11.92 to 12.89)	13.10 (12.59 to 13.62)	15.01 (14.46 to 15.56)	**0.96 (0.86 to 1.07)**	**0.61 (0.46 to 0.76)**	**-0.35 (-0.54 to -0.17)**
Rural	<6	10.61 (10.11 to 11.12)	12.37 (11.95 to 12.79)	13.87 (13.42 to 14.32)	15.15 (14.66 to 15.64)	16.11 (15.24 to 16.99)	16.74 (15.88 to 17.59)	17.88 (17.03 to 18.73)	**1.36 (1.15 to 1.58)**	**1.01 (0.71 to 1.31)**	-0.35 (-0.73 to 0.02)

	6–7	7.78 (7.43 to 8.14)	9.12 (8.82 to 9.42)	10.05 (9.75 to 10.35)	11.73 (11.37 to 12.09)	13.04 (12.39 to 13.69)	13.01 (12.35 to 13.67)	13.60 (12.95 to 14.24)	**1.16 (1.01 to 1.31)**	**0.75 (0.53 to 0.98)**	**-0.40 (-0.67 to -0.14)**
	7–8	7.18 (6.90 to 7.47)	8.51 (8.24 to 8.77)	9.57 (9.29 to 9.85)	10.99 (10.67 to 11.32)	11.26 (10.70 to 11.82)	12.44 (11.81 to 13.06)	12.24 (11.60 to 12.87)	**1.09 (0.96 to 1.21)**	**0.58 (0.38 to 0.78)**	**-0.50 (-0.74 to -0.27)**
	≥8	11.49 (11.08 to 11.90)	13.71 (13.31 to 14.11)	15.62 (15.19 to 16.06)	18.19 (17.67 to 18.72)	18.52 (17.60 to 19.45)	19.13 (18.23 to 20.03)	21.31 (20.31 to 22.31)	**1.32 (1.17 to 1.47)**	**0.78 (0.54 to 1.03)**	**-0.53 (-0.82 to -0.25)**
**BMI group [a]**											
Underweight	<6	7.72 (6.89 to 8.55)	10.63 (9.93 to 11.34)	10.97 (10.25 to 11.70)	12.64 (11.78 to 13.50)	7.80 (6.27 to 9.34)	12.44 (10.77 to 14.10)	11.23 (9.70 to 12.77)	**1.27 (0.90 to 1.63)**	-0.24 (-0.81 to 0.32)	**-1.51 (-2.18 to -0.84)**
	6–7	4.69 (4.14 to 5.25)	6.19 (5.70 to 6.67)	6.71 (6.21 to 7.21)	7.52 (6.93 to 8.12)	4.53 (3.40 to 5.66)	6.75 (5.62 to 7.87)	6.31 (5.23 to 7.40)	**0.64 (0.41 to 0.88)**	**-0.43 (-0.76 to -0.10)**	**-1.07 (-1.48 to -0.66)**
	7–8	3.80 (3.34 to 4.26)	5.20 (4.80 to 5.59)	5.98 (5.53 to 6.43)	6.60 (6.09 to 7.11)	3.82 (2.93 to 4.70)	4.36 (3.60 to 5.12)	5.17 (4.25 to 6.09)	**0.76 (0.56 to 0.95)**	**-0.58 (-0.86 to -0.29)**	**-1.33 (-1.68 to -0.99)**
	≥8	6.65 (6.03 to 7.27)	9.42 (8.83 to 10.01)	9.82 (9.19 to 10.46)	11.52 (10.80 to 12.24)	5.69 (4.59 to 6.79)	8.79 (7.58 to 10.00)	8.83 (7.52 to 10.13)	**0.97 (0.73 to 1.21)**	**-0.71 (-1.07 to -0.36)**	**-1.68 (-2.11 to -1.25)**
Normal weight	<6	7.25 (6.84 to 7.67)	7.93 (7.61 to 8.25)	8.98 (8.65 to 9.30)	9.91 (9.53 to 10.28)	12.24 (11.49 to 12.98)	12.72 (12.01 to 13.42)	12.86 (12.18 to 13.55)	**0.84 (0.68 to 1.01)**	**1.04 (0.81 to 1.27)**	0.20 (-0.09 to 0.48)

	6–7	4.44 (4.23 to 4.66)	4.73 (4.55 to 4.91)	5.31 (5.12 to 5.49)	6.05 (5.83 to 6.28)	7.88 (7.43 to 8.32)	7.73 (7.30 to 8.16)	7.71 (7.29 to 8.12)	**0.50 (0.41 to 0.59)**	**0.59 (0.45 to 0.73)**	0.09 (-0.07 to 0.26)
	7–8	3.74 (3.56 to 3.92)	4.24 (4.08 to 4.40)	4.99 (4.82 to 5.16)	5.62 (5.42 to 5.82)	6.10 (5.76 to 6.44)	6.39 (6.03 to 6.75)	7.35 (6.97 to 7.73)	**0.59 (0.51 to 0.66)**	**0.48 (0.35 to 0.60)**	-0.11 (-0.25 to 0.04)
	≥8	5.82 (5.55 to 6.09)	6.65 (6.40 to 6.91)	7.73 (7.44 to 8.01)	9.00 (8.67 to 9.33)	9.29 (8.76 to 9.82)	9.92 (9.35 to 10.48)	11.85 (11.23 to 12.48)	**0.75 (0.64 to 0.86)**	**0.63 (0.45 to 0.80)**	-0.12 (-0.32 to 0.08)
Overweight	<6	9.54 (8.94 to 10.13)	10.57 (10.06 to 11.08)	11.81 (11.33 to 12.30)	12.94 (12.40 to 13.49)	14.12 (13.15 to 15.08)	14.96 (14.03 to 15.90)	16.06 (15.14 to 16.97)	**1.13 (0.89 to 1.38)**	**1.04 (0.71 to 1.37)**	-0.09 (-0.50 to 0.32)
	6–7	6.78 (6.42 to 7.13)	7.46 (7.17 to 7.76)	8.09 (7.79 to 8.40)	9.64 (9.29 to 10.00)	10.30 (9.67 to 10.94)	11.06 (10.43 to 11.69)	12.15 (11.48 to 12.82)	**0.79 (0.64 to 0.94)**	**0.84 (0.62 to 1.07)**	0.05 (-0.22 to 0.32)
	7–8	6.37 (6.04 to 6.70)	7.46 (7.16 to 7.76)	8.12 (7.81 to 8.42)	9.00 (8.68 to 9.33)	9.24 (8.68 to 9.80)	9.93 (9.36 to 10.51)	10.89 (10.27 to 11.51)	**0.76 (0.62 to 0.90)**	**0.52 (0.32 to 0.72)**	-0.23 (-0.48 to 0.01)
	≥8	11.32 (10.75 to 11.89)	12.46 (11.94 to 12.99)	14.49 (13.93 to 15.04)	15.40 (14.82 to 15.99)	15.94 (14.98 to 16.89)	17.09 (16.05 to 18.13)	19.06 (18.00 to 20.13)	**0.89 (0.70 to 1.09)**	**0.91 (0.62 to 1.19)**	0.01 (-0.34 to 0.36)
Obese	<6	12.03 (11.38 to 12.68)	12.86 (12.35 to 13.37)	13.72 (13.26 to 14.19)	15.57 (15.08 to 16.05)	17.64 (16.73 to 18.56)	18.17 (17.29 to 19.05)	19.47 (18.59 to 20.36)	**1.11 (0.86 to 1.35)**	**1.29 (0.98 to 1.59)**	0.18 (-0.21 to 0.57)

	6–7	8.94 (8.52 to 9.37)	9.22 (8.89 to 9.54)	10.14 (9.82 to 10.45)	11.68 (11.35 to 12.02)	13.12 (12.49 to 13.75)	13.64 (13.00 to 14.27)	14.49 (13.87 to 15.11)	**0.88 (0.72 to 1.04)**	**0.95 (0.73 to 1.17)**	0.07 (-0.20 to 0.33)
	7–8	9.10 (8.69 to 9.51)	9.56 (9.22 to 9.90)	10.70 (10.37 to 11.04)	11.60 (11.26 to 11.94)	11.68 (11.11 to 12.24)	12.87 (12.25 to 13.49)	13.78 (13.17 to 14.39)	**0.73 (0.58 to 0.89)**	**0.77 (0.56 to 0.98)**	0.04 (-0.23 to 0.30)
	≥8	15.40 (14.73 to 16.08)	16.52 (15.92 to 17.13)	18.90 (18.27 to 19.53)	20.40 (19.79 to 21.01)	20.52 (19.55 to 21.48)	20.55 (19.54 to 21.56)	22.74 (21.67 to 23.81)	**1.04 (0.83 to 1.25)**	**0.58 (0.30 to 0.87)**	**-0.46 (-0.81 to -0.10)**
**Subjective Health-level**											
High	<6	2.75 (2.47 to 3.03)	3.58 (3.32 to 3.85)	3.82 (3.57 to 4.07)	5.00 (4.68 to 5.32)	7.44 (6.85 to 8.04)	7.44 (6.82 to 8.06)	7.25 (6.65 to 7.84)	**0.58 (0.46 to 0.70)**	**0.82 (0.62 to 1.01)**	**0.23 (0.00 to 0.47)**
	6–7	1.97 (1.83 to 2.12)	2.37 (2.24 to 2.50)	2.76 (2.62 to 2.90)	3.51 (3.34 to 3.69)	5.22 (4.88 to 5.56)	5.03 (4.70 to 5.36)	4.86 (4.54 to 5.19)	**0.43 (0.37 to 0.50)**	**0.52 (0.42 to 0.63)**	0.09 (-0.04 to 0.22)
	7–8	1.87 (1.74 to 2.00)	2.30 (2.18 to 2.42)	2.74 (2.61 to 2.88)	3.36 (3.20 to 3.52)	4.38 (4.12 to 4.64)	4.64 (4.34 to 4.94)	4.50 (4.21 to 4.80)	**0.43 (0.37 to 0.49)**	**0.40 (0.31 to 0.50)**	-0.03 (-0.14 to 0.09)
	≥8	2.49 (2.30 to 2.69)	3.07 (2.89 to 3.25)	3.74 (3.53 to 3.95)	4.68 (4.43 to 4.92)	6.27 (5.87 to 6.67)	6.46 (5.99 to 6.93)	6.35 (5.88 to 6.81)	**0.49 (0.41 to 0.57)**	**0.52 (0.39 to 0.64)**	0.03 (-0.12 to 0.18)
Middle	<6	6.55 (6.11 to 6.99)	7.17 (6.86 to 7.49)	8.35 (8.04 to 8.66)	10.29 (9.93 to 10.66)	13.94 (13.19 to 14.69)	13.81 (13.13 to 14.50)	13.55 (12.90 to 14.20)	**1.24 (1.07 to 1.41)**	**1.22 (0.99 to 1.45)**	-0.02 (-0.31 to 0.27)
	6–7	5.62 (5.36 to 5.88)	5.80 (5.59 to 6.00)	6.89 (6.69 to 7.10)	8.23 (7.99 to 8.47)	11.79 (11.25	11.23 (10.74	11.63 (11.14	**0.87 (0.76 to 0.98)**	**1.21 (1.05 to 1.38)**	**0.34 (0.14 to 0.54)**

						to 12.34)	to 11.72)	to 12.12)			
	7–8	5.25 (5.01 to 5.49)	5.73 (5.53 to 5.93)	7.02 (6.80 to 7.23)	8.13 (7.90 to 8.36)	10.91 (10.43 to 11.38)	10.32 (9.87 to 10.76)	11.42 (10.93 to 11.90)	**0.92 (0.82 to 1.02)**	**1.09 (0.93 to 1.25)**	0.17 (-0.02 to 0.35)
	≥8	6.91 (6.57 to 7.25)	7.86 (7.56 to 8.17)	9.98 (9.63 to 10.33)	11.90 (11.52 to 12.28)	16.57 (15.80 to 17.34)	15.41 (14.67 to 16.15)	17.56 (16.75 to 18.37)	**1.21 (1.08 to 1.34)**	**1.36 (1.15 to 1.57)**	0.15 (-0.10 to 0.39)
Low	<6	20.82 (20.05 to 21.58)	22.37 (21.77 to 22.97)	24.56 (23.96 to 25.16)	24.60 (23.99 to 25.22)	28.28 (26.95 to 29.62)	26.99 (25.85 to 28.13)	29.05 (27.96 to 30.13)	**1.23 (0.91 to 1.54)**	**1.51 (1.11 to 1.91)**	0.28 (-0.23 to 0.79)
	6–7	20.71 (19.97 to 21.45)	21.98 (21.37 to 22.59)	22.99 (22.39 to 23.60)	24.50 (23.86 to 25.15)	27.47 (26.03 to 28.91)	26.33 (25.08 to 27.59)	28.35 (27.18 to 29.53)	**1.07 (0.75 to 1.39)**	**1.22 (0.78 to 1.66)**	0.15 (-0.39 to 0.69)
	7–8	20.74 (20.01 to 21.47)	22.40 (21.77 to 23.03)	23.54 (22.92 to 24.17)	24.31 (23.67 to 24.96)	25.99 (24.54 to 27.45)	24.94 (23.65 to 26.24)	29.79 (28.47 to 31.10)	**1.04 (0.72 to 1.35)**	**1.41 (0.94 to 1.88)**	0.37 (-0.19 to 0.94)
	≥8	32.38 (31.45 to 33.31)	34.78 (33.94 to 35.63)	37.22 (36.36 to 38.07)	39.43 (38.54 to 40.33)	41.50 (39.60 to 43.41)	38.00 (36.31 to 39.68)	44.04 (42.42 to 45.66)	**1.30 (0.98 to 1.62)**	**0.97 (0.51 to 1.43)**	-0.33 (-0.90 to 0.23)
**Depression counseling**											
Yes	<6	11.27 (9.48 to 13.06)	12.26 (10.86 to 13.67)	12.57 (11.33 to 13.80)	14.92 (13.53 to 16.31)	14.52 (12.15 to 16.88)	14.78 (12.75 to 16.82)	15.38 (13.29 to 17.47)	**0.97 (0.29 to 1.66)**	0.24 (-0.55 to 1.03)	-0.74 (-1.78 to 0.31)

	6–7	7.32 (5.86 to 8.78)	8.22 (6.91 to 9.53)	8.99 (7.84 to 10.15)	9.71 (8.42 to 11.00)	9.85 (7.73 to 11.98)	11.16 (9.01 to 13.32)	8.06 (6.43 to 9.70)	**0.66 (0.04 to 1.28)**	-0.27 (-0.95 to 0.41)	**-0.93 (-1.85 to -0.01)**
	7–8	6.98 (5.44 to 8.52)	8.43 (7.00 to 9.87)	9.17 (7.86 to 10.47)	7.94 (6.70 to 9.18)	8.32 (6.41 to 10.22)	6.15 (4.59 to 7.72)	10.18 (8.24 to 12.12)	0.27 (-0.34 to 0.88)	0.16 (-0.53 to 0.85)	-0.11 (-1.03 to 0.81)
	≥8	12.56 (10.06 to 15.07)	14.71 (12.62 to 16.79)	14.35 (12.48 to 16.22)	12.68 (10.94 to 14.42)	14.45 (11.62 to 17.29)	11.40 (9.10 to 13.71)	15.94 (13.27 to 18.61)	-0.20 (-0.96 to 0.56)	0.69 (-0.16 to 1.55)	0.89 (-0.25 to 2.04)
No	<6	8.97 (8.67 to 9.27)	10.01 (9.77 to 10.25)	11.09 (10.86 to 11.33)	12.57 (12.31 to 12.83)	14.36 (13.86 to 14.86)	15.01 (14.53 to 15.49)	15.69 (15.22 to 16.16)	**1.13 (1.01 to 1.25)**	**1.08 (0.91 to 1.24)**	-0.05 (-0.25 to 0.15)
	6–7	6.07 (5.90 to 6.25)	6.57 (6.43 to 6.72)	7.34 (7.20 to 7.49)	8.74 (8.57 to 8.91)	10.05 (9.73 to 10.38)	10.36 (10.04 to 10.67)	10.92 (10.61 to 11.24)	**0.80 (0.73 to 0.87)**	**0.76 (0.65 to 0.87)**	**-0.04 (-0.17 to 0.09)**
	7–8	5.48 (5.33 to 5.64)	6.22 (6.09 to 6.36)	7.14 (7.00 to 7.28)	8.20 (8.04 to 8.36)	8.43 (8.16 to 8.70)	9.06 (8.78 to 9.34)	9.98 (9.68 to 10.28)	**0.81 (0.74 to 0.87)**	**0.55 (0.45 to 0.65)**	**-0.26 (-0.38 to -0.14)**
	≥8	8.81 (8.57 to 9.05)	9.99 (9.77 to 10.21)	11.68 (11.43 to 11.92)	13.48 (13.21 to 13.75)	13.50 (13.06 to 13.93)	14.28 (13.82 to 14.75)	16.15 (15.66 to 16.65)	**1.04 (0.95 to 1.13)**	**0.62 (0.49 to 0.75)**	**-0.42 (-0.58 to -0.26)**
**Stress counseling**											
Yes	<6	13.23 (10.73 to 15.72)	14.43 (12.43 to 16.42)	15.34 (13.59 to 17.10)	16.59 (14.77 to 18.40)	15.02 (12.12 to 17.92)	16.46 (13.49 to 19.43)	17.91 (15.29 to 20.53)	0.74 (-0.21 to 1.70)	0.35 (-0.65 to 1.36)	-0.39 (-1.78 to 1.00)

	6–7	10.57 (8.14 to 12.99)	10.83 (8.75 to 12.92)	11.48 (9.69 to 13.26)	12.16 (10.24 to 14.08)	9.83 (7.17 to 12.49)	12.06 (9.15 to 14.98)	12.43 (9.64 to 15.22)	0.65 (-0.33 to 1.63)	-0.04 (-1.12 to 1.04)	**-0.69 (-2.15 to 0.76)**
	7–8	7.83 (5.72 to 9.93)	9.73 (7.57 to 11.89)	12.19 (10.17 to 14.20)	11.03 (9.16 to 12.90)	10.69 (7.74 to 13.64)	10.17 (7.69 to 12.65)	11.39 (8.78 to 14.00)	**0.93 (0.03 to 1.83)**	-0.11 (-1.12 to 0.91)	-1.04 (-2.40 to 0.32)
	≥8	13.54 (10.69 to 16.39)	16.88 (14.08 to 19.69)	18.56 (16.07 to 21.05)	15.86 (13.53 to 18.19)	15.18 (11.59 to 18.76)	15.68 (12.56 to 18.80)	22.61 (18.98 to 26.24)	0.28 (-0.65 to 1.20)	**1.71 (0.60 to 2.82)**	1.43 (-0.01 to 2.88)
No	<6	8.96 (8.66 to 9.25)	10.00 (9.76 to 10.23)	11.05 (10.82 to 11.28)	12.57 (12.31 to 12.82)	14.35 (13.85 to 14.85)	14.95 (14.48 to 15.42)	15.60 (15.13 to 16.06)	**1.13 (1.01 to 1.25)**	**1.05 (0.89 to 1.22)**	-0.07 (-0.28 to 0.13)
	6–7	6.06 (5.89 to 6.23)	6.56 (6.42 to 6.71)	7.33 (7.19 to 7.47)	8.73 (8.56 to 8.90)	10.05 (9.73 to 10.37)	10.35 (10.04 to 10.67)	10.83 (10.52 to 11.14)	**0.80 (0.73 to 0.87)**	**0.74 (0.63 to 0.85)**	**-0.06 (-0.19 to 0.07)**
	7–8	5.49 (5.33 to 5.64)	6.23 (6.10 to 6.37)	7.13 (6.99 to 7.27)	8.17 (8.01 to 8.33)	8.40 (8.13 to 8.67)	8.98 (8.70 to 9.26)	9.96 (9.67 to 10.26)	**0.80 (0.73 to 0.86)**	**0.54 (0.45 to 0.64)**	**-0.25 (-0.37 to -0.14)**
	≥8	8.81 (8.57 to 9.05)	10.00 (9.78 to 10.22)	11.64 (11.40 to 11.88)	13.43 (13.16 to 13.69)	13.49 (13.06 to 13.92)	37.14 (35.84 to 38.44)	16.00 (15.51 to 16.49)	**1.03 (0.94 to 1.11)**	**0.60 (0.46 to 0.73)**	**-0.43 (-0.59 to -0.27)**
**Level of education**											
High school or lower education	<6	14.46 (13.93 to 14.98)	16.34 (15.91 to 16.77)	19.19 (18.74 to 19.65)	20.51 (20.02 to 20.99)	21.54 (20.65 to 22.43)	22.67 (21.82 to 23.52)	24.20 (23.35 to 25.04)	**2.15 (1.92 to 2.38)**	**1.21 (0.90 to 1.52)**	**-0.94 (-1.33 to -0.55)**

	6–7	12.61 (12.17 to 13.04)	14.78 (14.39 to 15.18)	16.89 (16.46 to 17.31)	19.34 (18.87 to 19.81)	19.80 (18.97 to 20.63)	21.21 (20.35 to 22.07)	22.39 (21.55 to 23.24)	**2.25 (2.04 to 2.46)**	**1.06 (0.74 to 1.37)**	**-1.20 (-1.57 to -0.82)**
	7–8	12.29 (11.87 to 12.71)	14.97 (14.58 to 15.36)	17.17 (16.74 to 17.59)	19.07 (18.61 to 19.53)	19.52 (18.71 to 20.33)	21.32 (20.45 to 22.19)	23.36 (22.45 to 24.28)	**2.28 (2.08 to 2.49)**	**1.35 (1.03 to 1.68)**	**-0.93 (-1.31 to -0.55)**
	≥8	20.79 (20.18 to 21.41)	24.90 (24.31 to 25.48)	29.95 (29.29 to 30.61)	33.92 (33.22 to 34.63)	35.13 (33.91 to 36.35)	8.68 (8.26 to 9.10)	38.95 (37.69 to 40.22)	**2.47 (2.25 to 2.69)**	**1.13 (0.79 to 1.47)**	**-1.34 (-1.74 to -0.94)**
College or higher education	<6	5.13 (4.82 to 5.44)	6.04 (5.79 to 6.29)	6.57 (6.33 to 6.80)	8.04 (7.77 to 8.31)	9.88 (9.32 to 10.43)	10.41 (9.88 to 10.94)	10.61 (10.11 to 11.12)	**0.91 (0.78 to 1.03)**	**0.89 (0.71 to 1.07)**	-0.02 (-0.23 to 0.20)
	6–7	3.77 (3.60 to 3.93)	4.07 (3.94 to 4.21)	4.86 (4.72 to 4.99)	5.84 (5.68 to 6.00)	7.07 (6.75 to 7.39)	7.51 (7.20 to 7.82)	7.82 (7.52 to 8.13)	**0.67 (0.60 to 0.74)**	**0.69 (0.58 to 0.79)**	0.02 (-0.11 to 0.14)
	7–8	3.39 (3.25 to 3.53)	3.82 (3.69 to 3.94)	4.69 (4.56 to 4.82)	5.47 (5.32 to 5.62)	5.84 (5.58 to 6.09)	6.46 (6.19 to 6.73)	7.09 (6.81 to 7.38)	**0.67 (0.61 to 0.73)**	**0.51 (0.41 to 0.60)**	**-0.16 (-0.27 to -0.05)**
	≥8	4.51 (4.30 to 4.72)	5.21 (5.02 to 5.40)	6.37 (6.16 to 6.59)	7.60 (7.36 to 7.84)	7.85 (7.46 to 8.23)	8.33 (7.86 to 8.80)	10.28 (9.81 to 10.75)	**0.78 (0.69 to 0.86)**	**0.64 (0.51 to 0.77)**	-0.14 (-0.29 to 0.02)
**Household income**											
Lowest and second quartile	<6	5.92 (5.51 to 6.33)	6.49 (6.19 to 6.79)	6.64 (6.36 to 6.92)	8.20 (7.88 to 8.51)	9.68 (9.05 to 10.31)	10.23 (9.64 to 10.83)	11.22 (10.64 to 11.80)	**0.74 (0.59 to 0.89)**	**0.90 (0.70 to 1.10)**	0.16 (-0.09 to 0.41)

	6–7	4.21 (4.00 to 4.42)	4.29 (4.14 to 4.45)	4.86 (4.70 to 5.02)	6.01 (5.83 to 6.19)	6.55 (6.21 to 6.89)	7.62 (7.27 to 7.98)	7.79 (7.46 to 8.13)	**0.59 (0.51 to 0.67)**	**0.63 (0.51 to 0.75)**	0.04 (-0.10 to 0.18)
	7–8	3.72 (3.54 to 3.91)	4.00 (3.85 to 4.14)	4.56 (4.40 to 4.72)	5.42 (5.25 to 5.58)	5.90 (5.61 to 6.18)	6.35 (6.04 to 6.65)	7.21 (6.90 to 7.52)	**0.56 (0.48 to 0.63)**	**0.53 (0.43 to 0.64)**	-0.02 (-0.15 to 0.11)
	≥8	5.66 (5.36 to 5.96)	5.79 (5.55 to 6.03)	6.57 (6.30 to 6.84)	7.57 (7.29 to 7.84)	8.16 (7.70 to 8.62)	23.70 (22.84 to 24.57)	10.28 (9.75 to 10.81)	**0.50 (0.39 to 0.60)**	**0.58 (0.43 to 0.72)**	0.08 (-0.10 to 0.26)
Third and highest quartile	<6	11.05 (10.65 to 11.45)	13.18 (12.83 to 13.52)	14.86 (14.52 to 15.21)	17.29 (16.90 to 17.69)	18.80 (18.07 to 19.54)	19.57 (18.87 to 20.28)	20.53 (19.83 to 21.24)	**2.00 (1.83 to 2.18)**	**1.21 (0.95 to 1.46)**	**-0.79 (-1.10 to -0.48)**
	6–7	8.01 (7.74 to 8.28)	9.82 (9.57 to 10.07)	10.74 (10.49 to 10.99)	13.75 (13.42 to 14.08)	15.98 (15.35 to 16.60)	15.52 (14.94 to 16.10)	17.16 (16.54 to 17.78)	**1.67 (1.54 to 1.80)**	**1.16 (0.95 to 1.38)**	**-0.51 (-0.76 to -0.26)**
	7–8	7.30 (7.07 to 7.54)	9.47 (9.23 to 9.72)	10.62 (10.37 to 10.87)	13.45 (13.14 to 13.77)	13.34 (12.81 to 13.87)	14.37 (13.81 to 14.92)	16.42 (15.80 to 17.05)	**1.77 (1.66 to 1.89)**	**0.85 (0.65 to 1.06)**	**-0.92 (-1.16 to -0.69)**
	≥8	11.49 (11.14 to 11.83)	14.87 (14.50 to 15.24)	17.18 (16.79 to 17.57)	22.31 (21.82 to 22.80)	21.88 (21.09 to 22.68)	14.12 (13.01 to 15.24)	26.56 (25.64 to 27.47)	**2.16 (2.02 to 2.30)**	**1.01 (0.77 to 1.25)**	**-1.15 (-1.43 to -0.87)**
**Smoking status**											
Smoker	<6	7.56 (6.98 to 8.14)	7.96 (7.50 to 8.42)	9.04 (8.55 to 9.53)	11.01 (10.43 to 11.59)	13.54 (12.38 to 14.70)	14.53 (13.34 to 15.72)	14.60 (13.45 to 15.74)	**1.18 (0.93 to 1.42)**	**1.28 (0.89 to 1.67)**	0.11 (-0.35 to 0.57)

	6–7	5.33 (5.01 to 5.65)	5.88 (5.60 to 6.17)	6.73 (6.43 to 7.02)	8.27 (7.90 to 8.64)	10.50 (9.70 to 11.30)	11.09 (10.33 to 11.85)	12.09 (11.29 to 12.88)	**0.91 (0.76 to 1.05)**	**1.29 (1.03 to 1.55)**	**0.38 (0.09 to 0.68)**
	7–8	5.50 (5.20 to 5.80)	6.15 (5.86 to 6.43)	7.24 (6.91 to 7.56)	8.38 (8.00 to 8.75)	8.86 (8.20 to 9.52)	9.58 (8.85 to 10.30)	10.10 (9.39 to 10.81)	**0.91 (0.77 to 1.05)**	**0.55 (0.32 to 0.79)**	**-0.36 (-0.63 to -0.08)**
	≥8	8.15 (7.69 to 8.61)	9.32 (8.86 to 9.77)	11.29 (10.76 to 11.82)	12.39 (11.79 to 12.99)	13.13 (12.15 to 14.12)	24.66 (23.30 to 26.02)	15.92 (14.81 to 17.03)	**1.02 (0.83 to 1.21)**	**0.98 (0.66 to 1.29)**	-0.05 (-0.42 to 0.33)
Ex-smoker	<6	12.11 (11.25 to 12.97)	13.65 (12.97 to 14.32)	15.43 (14.78 to 16.08)	16.61 (15.92 to 17.29)	17.97 (16.68 to 19.25)	18.71 (17.53 to 19.90)	19.11 (18.02 to 20.19)	**1.38 (1.04 to 1.71)**	**0.87 (0.46 to 1.27)**	-0.51 (-1.04 to 0.01)
	6–7	9.83 (9.26 to 10.39)	10.30 (9.88 to 10.72)	11.18 (10.77 to 11.59)	12.71 (12.26 to 13.16)	13.52 (12.67 to 14.36)	13.84 (13.03 to 14.65)	14.76 (14.00 to 15.53)	**0.94 (0.72 to 1.15)**	**0.68 (0.40 to 0.95)**	-0.26 (-0.61 to 0.09)
	7–8	9.63 (9.10 to 10.17)	10.52 (10.10 to 10.95)	11.99 (11.57 to 12.41)	12.96 (12.51 to 13.41)	12.72 (11.97 to 13.47)	13.82 (13.05 to 14.60)	14.69 (13.93 to 15.44)	**1.08 (0.87 to 1.28)**	**0.56 (0.30 to 0.83)**	**-0.51 (-0.85 to -0.18)**
	≥8	17.12 (16.25 to 17.99)	19.39 (18.64 to 20.14)	22.50 (21.72 to 23.29)	25.24 (24.44 to 26.04)	23.16 (21.89 to 24.43)	11.61 (11.12 to 12.11)	25.91 (24.67 to 27.16)	**1.77 (1.49 to 2.05)**	0.12 (-0.23 to 0.48)	**-1.65 (-2.10 to -1.19)**
Non-smoker	<6	8.89 (8.53 to 9.25)	9.91 (9.62 to 10.20)	10.71 (10.43 to 10.98)	12.09 (11.78 to 12.40)	13.64 (13.06 to 14.22)	14.13 (13.58 to 14.68)	14.87 (14.32 to 15.42)	**0.94 (0.80 to 1.08)**	**0.95 (0.76 to 1.14)**	0.01 (-0.23 to 0.25)

	6–7	5.51 (5.31 to 5.72)	5.87 (5.71 to 6.04)	6.53 (6.36 to 6.70)	7.75 (7.55 to 7.94)	8.93 (8.56 to 9.30)	9.16 (8.79 to 9.53)	9.11 (8.75 to 9.47)	**0.63 (0.54 to 0.71)**	**0.52 (0.40 to 0.64)**	-0.11 (-0.25 to 0.04)
	7–8	4.62 (4.44 to 4.79)	5.23 (5.08 to 5.38)	5.86 (5.71 to 6.02)	6.83 (6.65 to 7.01)	7.16 (6.86 to 7.47)	7.54 (7.23 to 7.86)	8.38 (8.04 to 8.72)	**0.61 (0.54 to 0.68)**	**0.47 (0.36 to 0.58)**	**-0.14 (-0.27 to -0.01)**
	≥8	7.53 (7.26 to 7.80)	8.30 (8.05 to 8.54)	9.36 (9.10 to 9.62)	10.80 (10.51 to 11.10)	11.32 (10.85 to 11.80)	16.56 (15.96 to 17.16)	13.10 (12.54 to 13.67)	**0.68 (0.58 to 0.78)**	**0.55 (0.40 to 0.70)**	-0.13 (-0.31 to 0.05)
**Alcohol consumption**											
Non-drinker and 1day/month	<6	10.88 (10.48 to 11.29)	12.46 (12.12 to 12.79)	13.72 (13.39 to 14.05)	15.14 (14.79 to 15.49)	16.26 (15.64 to 16.88)	16.95 (16.37 to 17.54)	17.98 (17.39 to 18.57)	**1.30 (1.14 to 1.46)**	**0.93 (0.72 to 1.15)**	**-0.37 (-0.63 to -0.10)**
	6–7	7.42 (7.17 to 7.68)	8.15 (7.94 to 8.37)	9.18 (8.96 to 9.40)	10.89 (10.64 to 11.14)	11.71 (11.28 to 12.14)	12.13 (11.71 to 12.56)	12.39 (11.98 to 12.80)	**1.01 (0.91 to 1.12)**	**0.58 (0.44 to 0.73)**	**-0.43 (-0.61 to -0.25)**
	7–8	6.40 (6.18 to 6.61)	7.55 (7.35 to 7.75)	8.84 (8.63 to 9.05)	10.09 (9.85 to 10.32)	10.08 (9.70 to 10.45)	10.58 (10.21 to 10.95)	11.89 (11.47 to 12.30)	**1.11 (1.01 to 1.21)**	**0.51 (0.37 to 0.65)**	**-0.60 (-0.77 to -0.43)**
	≥8	10.64 (10.31 to 10.97)	12.29 (11.97 to 12.60)	14.42 (14.08 to 14.77)	16.94 (16.56 to 17.32)	16.17 (15.58 to 16.75)	8.40 (7.77 to 9.03)	19.29 (18.64 to 19.93)	**1.37 (1.25 to 1.50)**	**0.49 (0.31 to 0.67)**	**-0.88 (-1.10 to -0.66)**
2–9 days/month	<6	5.44 (5.03 to 5.86)	5.94 (5.62 to 6.26)	6.71 (6.40 to 7.03)	8.15 (7.76 to 8.54)	9.94 (9.10 to 10.78)	10.04 (9.26 to 10.82)	10.20 (9.45 to 10.94)	**0.89 (0.72 to 1.06)**	**0.77 (0.52 to 1.03)**	-0.11 (-0.42 to 0.20)

	6–7	3.99 (3.77 to 4.22)	4.36 (4.18 to 4.54)	5.01 (4.82 to 5.19)	5.72 (5.51 to 5.94)	6.99 (6.52 to 7.45)	6.85 (6.41 to 7.30)	8.09 (7.63 to 8.55)	**0.59 (0.50 to 0.68)**	**0.69 (0.54 to 0.84)**	0.10 (-0.08 to 0.28)
	7–8	3.87 (3.67 to 4.08)	4.24 (4.07 to 4.42)	4.78 (4.60 to 4.96)	5.45 (5.25 to 5.65)	5.60 (5.24 to 5.97)	5.92 (5.52 to 6.32)	6.71 (6.31 to 7.11)	**0.49 (0.40 to 0.57)**	**0.37 (0.24 to 0.50)**	-0.12 (-0.28 to 0.04)
	≥8	5.21 (4.90 to 5.52)	5.85 (5.56 to 6.13)	6.84 (6.53 to 7.15)	7.41 (7.08 to 7.74)	7.73 (7.15 to 8.30)	19.00 (16.66 to 21.34)	9.46 (8.79 to 10.13)	**0.54 (0.42 to 0.66)**	**0.53 (0.34 to 0.71)**	-0.01 (-0.23 to 0.20)
Over 10 days/month	<6	9.05 (8.06 to 10.04)	9.69 (8.90 to 10.47)	11.56 (10.75 to 12.37)	11.62 (10.76 to 12.49)	12.35 (10.57 to 14.13)	12.62 (10.90 to 14.34)	14.35 (12.62 to 16.07)	**0.99 (0.58 to 1.40)**	**0.81 (0.19 to 1.42)**	-0.19 (-0.92 to 0.55)
	6–7	7.92 (7.16 to 8.67)	8.49 (7.88 to 9.11)	8.84 (8.27 to 9.41)	10.16 (9.48 to 10.83)	10.58 (9.14 to 12.03)	12.13 (10.59 to 13.68)	12.23 (10.79 to 13.67)	**0.60 (0.30 to 0.91)**	**0.78 (0.30 to 1.26)**	0.18 (-0.39 to 0.74)
	7–8	7.97 (7.23 to 8.71)	8.53 (7.92 to 9.13)	9.60 (8.99 to 10.21)	9.99 (9.31 to 10.66)	8.93 (7.66 to 10.20)	10.60 (9.18 to 12.02)	12.09 (10.62 to 13.56)	**0.66 (0.35 to 0.96)**	**0.61 (0.13 to 1.09)**	-0.05 (-0.62 to 0.53)
	≥8	12.42 (11.36 to 13.47)	13.53 (12.61 to 14.46)	16.15 (15.13 to 17.16)	16.84 (15.76 to 17.93)	16.44 (14.52 to 18.36)	16.44 (14.52 to 18.36)	19.57 (17.36 to 21.77)	**0.97 (0.60 to 1.34)**	0.56 (-0.01 to 1.13)	-0.41 (-1.09 to 0.27)

Abbreviations: BMI, body mass index; CI, confidence interval; KCHS, Korea Community Health Survey.

β values indicate the average annual change (%) in the weighted prevalence, multiplied by 100 for interpretability.

The values in bold font represent significant variance ($p<0.05$).

[a] According to the Asian-Pacific guidelines, BMI is divided into four groups: underweight (<18.5 kg/ m^2), normal (18.5–22.9 kg/ m^2), overweight (23.0–24.9 kg/ m^2), and obese (≥25.0 kg/ m^2).

Table S4. National trends in sleep duration among individuals with both hypertension and diabetes, along with β-coefficients before and during the COVID–19 pandemic (weighted % [95% CI]).

Variables	Sleep duration (hours/day)	Pre-pandemic				During the pandemic			Trends in the pre-pandemic era, β (95% CI)	Trends in the pandemic era, β (95% CI)	β_{diff} between 2009-2019 and 2019-2022 (95% CI)
		2009-2010	2011-2013	2014-2016	2017-2019	2020	2021	2022			
**Overall**	<6	6.98 (6.69 to 7.27)	8.18 (7.94 to 8.43)	9.48 (9.24 to 9.73)	11.42 (11.13 to 11.71)	13.31 (12.74 to 13.88)	13.86 (13.32 to 14.39)	15.19 (14.64 to 15.73)	**1.28 (1.16 to 1.40)**	**1.21 (1.03 to 1.39)**	-0.07 (-0.29 to 0.14)
	6-7	3.96 (3.80 to 4.11)	4.44 (4.31 to 4.57)	5.22 (5.08 to 5.35)	6.52 (6.36 to 6.68)	7.72 (7.40 to 8.04)	7.71 (7.41 to 8.02)	8.45 (8.14 to 8.76)	**0.73 (0.66 to 0.79)**	**0.62 (0.52 to 0.72)**	**-0.40 (-0.56 to -0.24)**
	7-8	3.41 (3.28 to 3.54)	4.11 (3.99 to 4.23)	4.93 (4.80 to 5.06)	5.93 (5.78 to 6.07)	5.98 (5.73 to 6.23)	6.48 (6.22 to 6.74)	7.56 (7.27 to 7.86)	**0.71 (0.65 to 0.76)**	**0.45 (0.36 to 0.54)**	**-0.40 (-0.56 to -0.24)**
	≥8	5.26 (5.07 to 5.45)	6.18 (6.00 to 6.36)	7.27 (7.08 to 7.47)	8.85 (8.62 to 9.07)	8.72 (8.37 to 9.08)	9.19 (8.82 to 9.57)	10.76 (10.34 to 11.18)	**0.98 (0.90 to 1.06)**	**0.51 (0.38 to 0.64)**	**-0.47 (-0.62 to -0.31)**
**Sex**											
Male	<6	5.70 (5.31 to 6.10)	6.99 (6.64 to 7.34)	8.67 (8.30 to 9.04)	10.83 (10.38 to 11.28)	13.04 (12.16 to 13.91)	13.72 (12.89 to 14.55)	14.89 (14.05 to 15.72)	**1.50 (1.33 to 1.67)**	**1.38 (1.10 to 1.66)**	-0.12 (-0.45 to 0.20)
	6-7	3.84 (3.63 to 4.05)	4.47 (4.29 to 4.66)	5.34 (5.15 to 5.54)	6.77 (6.53 to 7.01)	8.10 (7.62 to 8.57)	8.15 (7.69 to 8.60)	9.46 (8.97 to 9.95)	**0.81 (0.72 to 0.90)**	**0.84 (0.69 to 0.99)**	**-0.69 (-0.94 to -0.44)**
	7-8	3.72 (3.52 to 3.92)	4.68 (4.49 to 4.86)	5.91 (5.70 to 6.12)	6.80 (6.57 to 7.03)	6.62 (6.24 to 7.01)	7.71 (7.29 to 8.13)	8.68 (8.24 to 9.13)	**0.87 (0.78 to 0.95)**	**0.55 (0.41 to 0.68)**	**-0.69 (-0.94 to -0.44)**
	≥8	6.29 (5.97 to 6.60)	7.53 (7.23 to 7.83)	9.37 (9.03 to 9.71)	11.16 (10.78 to 11.53)	10.62 (10.05 to 11.19)	12.00 (11.36 to 12.64)	13.09 (12.41 to 13.76)	**1.34 (1.21 to 1.48)**	**0.63 (0.42 to 0.84)**	**-0.71 (-0.96 to -0.46)**
Female	<6	8.02 (7.61 to 8.43)	9.06 (8.73 to 9.39)	10.08 (9.75 to 10.40)	11.81 (11.44 to 12.18)	13.48 (12.77 to 14.20)	13.95 (13.27 to 14.63)	15.38 (14.70 to 16.07)	**1.04 (0.88 to 1.21)**	**1.07 (0.84 to 1.30)**	0.03 (-0.25 to 0.30)
	6-7	4.08 (3.86 to 4.29)	4.41 (4.23 to 4.58)	5.10 (4.92 to 5.28)	6.30 (6.08 to 6.51)	7.40 (6.98 to 7.82)	7.33 (6.93 to 7.74)	7.57 (7.17 to 7.96)	**0.63 (0.55 to 0.72)**	**0.41 (0.28 to 0.54)**	-0.17 (-0.36 to 0.02)
	7-8	3.14 (2.97 to 3.30)	3.65 (3.50 to 3.80)	4.13 (3.97 to 4.28)	5.21 (5.03 to 5.39)	5.43 (5.12 to 5.75)	5.44 (5.12 to 5.76)	6.63 (6.26 to 6.99)	**0.55 (0.48 to 0.62)**	**0.37 (0.26 to 0.48)**	-0.17 (-0.36 to 0.02)
	≥8	4.51 (4.29 to 4.74)	5.27 (5.06 to 5.48)	5.82 (5.60 to 6.04)	7.21 (6.96 to 7.47)	7.28 (6.86 to 7.71)	7.08 (6.65 to 7.50)	8.91 (8.41 to 9.41)	**0.67 (0.58 to 0.77)**	**0.38 (0.23 to 0.52)**	**-0.30 (-0.48 to -0.12)**
**Age, years**											

19–30	<6	0.03 (0.00 to 0.06)	0.05 (0.00 to 0.10)	0.09 (0.02 to 0.16)	0.12 (0.04 to 0.20)	N/A	0.15 (0.00 to 0.35)	0.37 (0.05 to 0.69)	**0.04 (0.01 to 0.07)**	0.06 (-0.03 to 0.16)	0.03 (-0.07 to 0.13)
	6-7	0.06 (0.02 to 0.11)	0.08 (0.03 to 0.13)	0.05 (0.02 to 0.08)	0.04 (0.01 to 0.07)	0.15 (0.05 to 0.25)	0.08 (0.01 to 0.15)	0.19 (0.05 to 0.32)	-0.01 (-0.03 to 0.02)	0.04 (0.00 to 0.08)	0.01 (-0.07 to 0.10)
	7-8	0.03 (0.00 to 0.06)	0.03 (0.01 to 0.06)	0.07 (0.04 to 0.11)	0.09 (0.04 to 0.14)	0.07 (0.02 to 0.12)	0.02 (0.00 to 0.05)	0.11 (0.04 to 0.18)	**0.03 (0.01 to 0.04)**	-0.01 (-0.03 to 0.02)	0.01 (-0.07 to 0.10)
	≥8	0.04 (0.01 to 0.08)	0.08 (0.04 to 0.13)	0.13 (0.06 to 0.19)	0.05 (0.02 to 0.08)	0.10 (0.00 to 0.19)	0.12 (0.02 to 0.22)	0.16 (0.04 to 0.29)	0.00 (-0.01 to 0.02)	**0.04 (0.01 to 0.07)**	0.03 (0.00 to 0.07)
31–40	<6	0.39 (0.23 to 0.55)	0.42 (0.28 to 0.57)	0.42 (0.28 to 0.55)	0.73 (0.52 to 0.95)	1.15 (0.65 to 1.66)	1.44 (0.92 to 1.96)	0.93 (0.48 to 1.38)	**0.12 (0.02 to 0.21)**	0.13 (-0.04 to 0.31)	0.02 (-0.18 to 0.21)
	6-7	0.18 (0.12 to 0.24)	0.30 (0.22 to 0.38)	0.40 (0.32 to 0.48)	0.45 (0.34 to 0.56)	0.64 (0.41 to 0.88)	0.57 (0.34 to 0.79)	0.69 (0.43 to 0.96)	**0.09 (0.05 to 0.13)**	0.08 (-0.01 to 0.17)	0.14 (-0.06 to 0.33)
	7-8	0.22 (0.15 to 0.30)	0.25 (0.19 to 0.31)	0.38 (0.30 to 0.46)	0.33 (0.25 to 0.41)	0.42 (0.26 to 0.58)	0.70 (0.47 to 0.92)	0.63 (0.43 to 0.83)	**0.05 (0.02 to 0.09)**	**0.12 (0.05 to 0.19)**	0.14 (-0.06 to 0.33)
	≥8	0.32 (0.22 to 0.42)	0.31 (0.22 to 0.40)	0.29 (0.21 to 0.38)	0.44 (0.32 to 0.56)	0.55 (0.33 to 0.77)	0.44 (0.23 to 0.65)	0.75 (0.45 to 1.05)	0.03 (-0.02 to 0.08)	0.07 (-0.02 to 0.17)	0.04 (-0.07 to 0.15)
41–50	<6	1.83 (1.50 to 2.16)	2.33 (2.03 to 2.64)	2.32 (2.04 to 2.60)	2.89 (2.52 to 3.26)	3.15 (2.44 to 3.85)	2.83 (2.19 to 3.48)	3.87 (3.11 to 4.62)	**0.30 (0.13 to 0.46)**	**0.27 (0.01 to 0.53)**	-0.03 (-0.34 to 0.28)
	6-7	1.48 (1.30 to 1.67)	1.42 (1.28 to 1.57)	1.62 (1.47 to 1.77)	1.98 (1.78 to 2.17)	1.72 (1.39 to 2.05)	2.47 (2.08 to 2.87)	2.72 (2.30 to 3.14)	**0.20 (0.11 to 0.29)**	**0.25 (0.10 to 0.40)**	0.19 (-0.13 to 0.51)
	7-8	1.27 (1.11 to 1.43)	1.38 (1.24 to 1.52)	1.46 (1.32 to 1.60)	1.70 (1.53 to 1.87)	1.93 (1.61 to 2.26)	1.88 (1.55 to 2.22)	2.42 (2.03 to 2.81)	**0.16 (0.08 to 0.24)**	**0.20 (0.06 to 0.33)**	0.19 (-0.13 to 0.51)
	≥8	1.92 (1.64 to 2.19)	1.80 (1.58 to 2.01)	1.82 (1.58 to 2.06)	2.02 (1.75 to 2.29)	2.50 (2.01 to 3.00)	2.61 (2.07 to 3.15)	2.53 (1.98 to 3.08)	0.02 (-0.12 to 0.16)	0.14 (-0.05 to 0.33)	0.12 (-0.12 to 0.35)
51–60	<6	7.01 (6.34 to 7.68)	7.17 (6.65 to 7.69)	7.70 (7.20 to 8.19)	8.23 (7.69 to 8.78)	8.79 (7.72 to 9.86)	9.75 (8.73 to 10.77)	9.16 (8.18 to 10.13)	**0.54 (0.26 to 0.83)**	**0.38 (0.01 to 0.75)**	-0.17 (-0.64 to 0.30)
	6-7	6.15 (5.67 to 6.63)	5.80 (5.47 to 6.14)	5.97 (5.65 to 6.29)	6.67 (6.31 to 7.04)	6.85 (6.22 to 7.49)	7.42 (6.78 to 8.06)	7.32 (6.69 to 7.96)	**0.27 (0.07 to 0.46)**	**0.28 (0.03 to 0.53)**	0.08 (-0.38 to 0.55)
	7-8	5.99 (5.55 to 6.44)	5.98 (5.62 to 6.34)	6.21 (5.87 to 6.55)	6.15 (5.80 to 6.50)	6.02 (5.44 to 6.60)	6.53 (5.88 to 7.18)	7.25 (6.60 to 7.91)	0.16 (-0.03 to 0.34)	**0.32 (0.07 to 0.57)**	0.08 (-0.38 to 0.55)
	≥8	7.80 (7.20 to 8.39)	8.03 (7.53 to 8.54)	8.77 (8.22 to 9.32)	8.05 (7.51 to 8.59)	9.24 (8.27 to 10.21)	8.30 (7.38 to 9.21)	9.14 (8.12 to 10.17)	0.18 (-0.10 to 0.46)	0.32 (-0.06 to 0.71)	0.14 (-0.33 to 0.61)
61–70	<6	19.26 (18.07 to 20.45)	19.72 (18.79 to 20.64)	20.99 (20.09 to 21.88)	20.36 (19.49 to 21.22)	21.21 (19.66 to 22.75)	19.59 (18.20 to 20.97)	21.54 (20.23 to 22.86)	0.43 (-0.04 to 0.90)	0.19 (-0.33 to 0.72)	-0.23 (-0.94 to 0.47)
	6-7	15.83 (14.91 to 16.75)	18.18 (17.37 to 18.98)	18.44 (17.68 to 19.20)	17.62 (16.91 to 18.33)	17.75 (16.55 to 18.96)	16.49 (15.39 to 17.59)	17.15 (16.10 to 18.20)	0.36 (-0.02 to 0.74)	-0.25 (-0.67 to 0.17)	-0.52 (-1.09 to 0.06)

	7-8	16.02 (15.10 to 16.93)	18.00 (17.22 to 18.78)	18.99 (18.23 to 19.76)	17.86 (17.16 to 18.55)	17.16 (16.01 to 18.31)	17.56 (16.43 to 18.68)	18.70 (17.57 to 19.83)	**0.50 (0.12 to 0.87)**	0.17 (-0.27 to 0.61)	-0.52 (-1.09 to 0.06)
	≥8	19.43 (18.41 to 20.46)	21.38 (20.44 to 22.32)	22.40 (21.43 to 23.37)	22.48 (21.54 to 23.41)	20.55 (19.13 to 21.97)	21.87 (20.38 to 23.35)	23.29 (21.85 to 24.73)	**1.12 (0.66 to 1.59)**	0.08 (-0.50 to 0.66)	**-1.04 (-1.78 to -0.29)**
>70	<6	21.44 (20.13 to 22.76)	27.86 (26.77 to 28.95)	33.05 (32.02 to 34.08)	34.54 (33.51 to 35.58)	35.38 (33.63 to 37.12)	36.76 (35.17 to 38.36)	38.07 (36.53 to 39.61)	**4.09 (3.54 to 4.63)**	**1.07 (0.46 to 1.69)**	**-3.01 (-3.83 to -2.20)**
	6-7	22.26 (20.91 to 23.61)	24.81 (23.74 to 25.87)	30.94 (29.87 to 32.01)	32.50 (31.44 to 33.56)	31.39 (29.70 to 33.08)	31.57 (29.90 to 33.24)	33.68 (32.08 to 35.29)	**3.59 (3.04 to 4.13)**	0.27 (-0.37 to 0.90)	**-1.40 (-1.93 to -0.86)**
	7-8	21.64 (20.27 to 23.00)	25.97 (24.85 to 27.09)	29.66 (28.58 to 30.74)	32.47 (31.42 to 33.51)	29.57 (27.97 to 31.17)	32.90 (31.23 to 34.58)	35.89 (34.14 to 37.64)	**3.51 (2.96 to 4.07)**	**1.01 (0.35 to 1.67)**	**-1.40 (-1.93 to -0.86)**
	≥8	22.90 (21.73 to 24.07)	26.72 (25.73 to 27.72)	30.95 (29.93 to 31.96)	34.86 (33.88 to 35.85)	33.32 (31.75 to 34.89)	34.63 (33.00 to 36.27)	36.79 (35.16 to 38.42)	**4.08 (3.59 to 4.56)**	0.49 (-0.12 to 1.11)	**-3.58 (-4.37 to -2.80)**
**Region of residence**											
Urban	<6	6.61 (6.28 to 6.95)	7.55 (7.28 to 7.83)	8.75 (8.48 to 9.03)	10.67 (10.35 to 11.00)	12.64 (11.99 to 13.28)	13.20 (12.60 to 13.80)	14.45 (13.83 to 15.06)	**1.19 (1.05 to 1.32)**	**1.17 (0.97 to 1.38)**	-0.01 (-0.26 to 0.23)
	6-7	3.63 (3.46 to 3.80)	4.00 (3.86 to 4.14)	4.69 (4.55 to 4.84)	5.93 (5.75 to 6.11)	7.09 (6.73 to 7.44)	7.18 (6.84 to 7.52)	7.91 (7.57 to 8.26)	**0.65 (0.58 to 0.72)**	**0.62 (0.51 to 0.73)**	**-0.35 (-0.54 to -0.17)**
	7-8	3.11 (2.96 to 3.27)	3.67 (3.54 to 3.81)	4.46 (4.32 to 4.60)	5.33 (5.16 to 5.49)	5.41 (5.13 to 5.69)	5.84 (5.55 to 6.12)	7.06 (6.73 to 7.38)	**0.63 (0.56 to 0.69)**	**0.45 (0.35 to 0.55)**	**-0.35 (-0.54 to -0.17)**
	≥8	4.79 (4.57 to 5.02)	5.58 (5.37 to 5.79)	6.58 (6.35 to 6.81)	7.93 (7.67 to 8.18)	7.94 (7.54 to 8.34)	8.30 (7.87 to 8.73)	9.84 (9.36 to 10.31)	**0.86 (0.76 to 0.96)**	**0.48 (0.34 to 0.63)**	**-0.38 (-0.55 to -0.20)**
Rural	<6	8.60 (8.06 to 9.15)	11.33 (10.84 to 11.81)	13.22 (12.69 to 13.74)	15.19 (14.58 to 15.80)	16.66 (15.56 to 17.76)	17.01 (15.91 to 18.11)	18.76 (17.66 to 19.87)	**1.79 (1.56 to 2.03)**	**1.31 (0.95 to 1.68)**	**-0.48 (-0.91 to -0.05)**
	6-7	5.58 (5.24 to 5.93)	6.89 (6.58 to 7.19)	8.24 (7.92 to 8.56)	9.90 (9.50 to 10.30)	11.32 (10.59 to 12.04)	10.81 (10.08 to 11.55)	11.53 (10.81 to 12.24)	**1.19 (1.05 to 1.34)**	**0.64 (0.41 to 0.87)**	**-0.53 (-0.82 to -0.25)**
	7-8	4.69 (4.43 to 4.95)	6.24 (5.98 to 6.50)	7.35 (7.07 to 7.64)	9.04 (8.70 to 9.39)	9.09 (8.52 to 9.67)	10.12 (9.46 to 10.78)	10.37 (9.68 to 11.06)	**1.16 (1.05 to 1.28)**	**0.55 (0.35 to 0.76)**	**-0.53 (-0.82 to -0.25)**

	≥8	6.87 (6.54 to 7.20)	8.51 (8.17 to 8.84)	10.10 (9.73 to 10.47)	12.68 (12.22 to 13.14)	12.33 (11.54 to 13.13)	13.29 (12.49 to 14.08)	15.03 (14.15 to 15.92)	**1.52 (1.36 to 1.67)**	**0.79 (0.52 to 1.05)**	**-0.73 (-1.03 to -0.42)**
**BMI group** [a]											
Underweight	<6	5.73 (4.91 to 6.55)	8.88 (8.12 to 9.65)	9.92 (9.10 to 10.74)	11.45 (10.49 to 12.40)	6.20 (4.66 to 7.74)	10.33 (8.53 to 12.13)	9.65 (8.00 to 11.30)	**1.44 (1.07 to 1.81)**	-0.31 (-0.91 to 0.30)	**-1.75 (-2.46 to -1.04)**
	6-7	2.93 (2.46 to 3.40)	4.31 (3.89 to 4.74)	4.47 (4.04 to 4.90)	5.61 (5.06 to 6.16)	2.43 (1.51 to 3.35)	4.64 (3.67 to 5.62)	4.02 (3.19 to 4.86)	**0.59 (0.39 to 0.78)**	**-0.46 (-0.73 to -0.19)**	**-1.68 (-2.11 to -1.25)**
	7-8	1.99 (1.68 to 2.31)	3.62 (3.26 to 3.98)	4.04 (3.65 to 4.43)	4.70 (4.24 to 5.15)	2.04 (1.35 to 2.73)	2.72 (2.13 to 3.31)	2.84 (2.17 to 3.51)	**0.64 (0.48 to 0.80)**	**-0.57 (-0.79 to -0.36)**	**-1.68 (-2.11 to -1.25)**
	≥8	3.68 (3.22 to 4.13)	5.97 (5.49 to 6.45)	5.79 (5.30 to 6.29)	7.58 (6.98 to 8.17)	3.53 (2.63 to 4.43)	5.49 (4.53 to 6.44)	4.89 (3.90 to 5.88)	**0.89 (0.69 to 1.10)**	**-0.79 (-1.09 to -0.50)**	**-1.69 (-2.05 to -1.33)**
Normal weight	<6	4.73 (4.37 to 5.09)	5.31 (5.02 to 5.60)	6.31 (6.00 to 6.62)	7.12 (6.76 to 7.48)	9.41 (8.65 to 10.17)	9.82 (9.10 to 10.55)	10.38 (9.67 to 11.10)	**0.70 (0.56 to 0.85)**	**1.03 (0.81 to 1.26)**	**0.33 (0.06 to 0.60)**
	6-7	2.31 (2.15 to 2.48)	2.48 (2.34 to 2.63)	3.00 (2.85 to 3.15)	3.37 (3.19 to 3.55)	4.77 (4.39 to 5.15)	4.51 (4.15 to 4.88)	4.97 (4.60 to 5.34)	**0.31 (0.25 to 0.38)**	**0.50 (0.39 to 0.61)**	-0.12 (-0.32 to 0.08)
	7-8	1.86 (1.73 to 2.00)	2.28 (2.15 to 2.40)	2.68 (2.55 to 2.82)	3.10 (2.94 to 3.26)	3.44 (3.17 to 3.72)	3.70 (3.40 to 3.99)	4.50 (4.17 to 4.83)	**0.34 (0.28 to 0.40)**	**0.38 (0.28 to 0.48)**	-0.12 (-0.32 to 0.08)
	≥8	2.98 (2.77 to 3.18)	3.51 (3.32 to 3.70)	4.09 (3.88 to 4.31)	5.09 (4.84 to 5.35)	5.18 (4.77 to 5.58)	5.59 (5.16 to 6.02)	7.00 (6.51 to 7.49)	**0.55 (0.46 to 0.64)**	**0.48 (0.33 to 0.62)**	-0.08 (-0.25 to 0.09)
Overweight	<6	7.50 (6.88 to 8.12)	8.90 (8.36 to 9.45)	10.24 (9.71 to 10.77)	11.65 (11.04 to 12.26)	13.26 (12.14 to 14.38)	14.11 (13.02 to 15.20)	16.16 (15.06 to 17.25)	**1.18 (0.93 to 1.43)**	**1.41 (1.04 to 1.77)**	0.22 (-0.22 to 0.66)
	6-7	4.50 (4.17 to 4.83)	5.20 (4.92 to 5.48)	5.87 (5.58 to 6.16)	7.22 (6.87 to 7.58)	8.01 (7.36 to 8.65)	7.91 (7.30 to 8.51)	9.33 (8.65 to 10.01)	**0.73 (0.60 to 0.87)**	**0.61 (0.39 to 0.82)**	0.01 (-0.34 to 0.36)
	7-8	4.01 (3.72 to 4.30)	4.98 (4.71 to 5.25)	5.86 (5.57 to 6.15)	6.64 (6.31 to 6.96)	6.54 (6.00 to 7.07)	7.28 (6.72 to 7.84)	8.76 (8.11 to 9.40)	**0.75 (0.63 to 0.88)**	**0.51 (0.31 to 0.70)**	0.01 (-0.34 to 0.36)
	≥8	6.92 (6.45 to 7.39)	7.52 (7.09 to 7.95)	9.06 (8.60 to 9.53)	9.95 (9.46 to 10.45)	10.31 (9.50 to 11.11)	11.07 (10.18 to 11.96)	12.71 (11.78 to 13.65)	**0.88 (0.68 to 1.07)**	**0.84 (0.54 to 1.13)**	-0.04 (-0.39 to 0.31)
Obese	<6	11.39 (10.66 to 12.13)	12.50 (11.90 to 13.11)	13.90 (13.34 to 14.47)	16.96 (16.34 to 17.58)	19.27 (18.10 to 20.44)	19.87 (18.72 to 21.02)	22.13 (20.95 to 23.31)	**1.65 (1.37 to 1.93)**	**1.61 (1.22 to 1.99)**	-0.04 (-0.52 to 0.43)
	6-7	7.37 (6.93 to 7.81)	7.77 (7.42 to 8.12)	8.90 (8.55 to 9.24)	10.81 (10.42 to 11.19)	12.47 (11.75 to 13.19)	12.77 (12.04 to 13.49)	13.74 (13.02 to 14.46)	**1.00 (0.83 to 1.16)**	**0.97 (0.74 to 1.21)**	**-0.46 (-0.81 to -0.10)**

	7-8	7.39 (6.96 to 7.83)	7.85 (7.49 to 8.20)	9.29 (8.92 to 9.66)	10.49 (10.11 to 10.86)	10.40 (9.79 to 11.02)	11.37 (10.70 to 12.04)	12.59 (11.90 to 13.27)	**0.88 (0.72 to 1.04)**	**0.66 (0.44 to 0.88)**	**-0.46 (-0.81 to -0.10)**
	≥8	10.48 (9.90 to 11.06)	11.79 (11.25 to 12.33)	13.55 (12.99 to 14.11)	15.14 (14.58 to 15.70)	14.85 (13.98 to 15.72)	14.96 (14.05 to 15.86)	17.09 (16.10 to 18.08)	**1.29 (1.06 to 1.52)**	**0.49 (0.18 to 0.81)**	**-0.80 (-1.19 to -0.40)**
**Subjective Health-level**											
High	<6	1.45 (1.23 to 1.67)	2.10 (1.88 to 2.32)	2.39 (2.18 to 2.60)	3.62 (3.32 to 3.92)	5.54 (4.95 to 6.12)	5.33 (4.73 to 5.92)	5.21 (4.65 to 5.77)	**0.55 (0.44 to 0.65)**	**0.58 (0.40 to 0.75)**	0.03 (-0.17 to 0.23)
	6-7	0.90 (0.80 to 1.00)	1.18 (1.08 to 1.27)	1.48 (1.37 to 1.58)	2.03 (1.89 to 2.17)	3.34 (3.05 to 3.62)	2.85 (2.57 to 3.12)	3.19 (2.91 to 3.48)	**0.30 (0.25 to 0.34)**	**0.40 (0.31 to 0.48)**	0.03 (-0.12 to 0.18)
	7-8	0.89 (0.79 to 0.98)	1.20 (1.10 to 1.29)	1.47 (1.37 to 1.58)	1.85 (1.73 to 1.97)	2.70 (2.48 to 2.92)	2.82 (2.58 to 3.07)	2.76 (2.52 to 3.00)	**0.26 (0.22 to 0.30)**	**0.32 (0.24 to 0.39)**	0.03 (-0.12 to 0.18)
	≥8	1.15 (1.02 to 1.27)	1.53 (1.40 to 1.65)	1.87 (1.72 to 2.02)	2.58 (2.40 to 2.76)	3.67 (3.36 to 3.99)	3.61 (3.26 to 3.97)	3.81 (3.44 to 4.17)	**0.36 (0.30 to 0.42)**	**0.39 (0.29 to 0.50)**	0.03 (-0.09 to 0.15)
Middle	<6	4.27 (3.89 to 4.65)	4.88 (4.58 to 5.18)	6.20 (5.89 to 6.51)	8.41 (8.02 to 8.79)	12.60 (11.74 to 13.47)	12.16 (11.38 to 12.93)	12.16 (11.42 to 12.90)	**1.31 (1.15 to 1.47)**	**1.36 (1.11 to 1.61)**	0.05 (-0.24 to 0.35)
	6-7	3.35 (3.12 to 3.57)	3.51 (3.33 to 3.68)	4.54 (4.35 to 4.73)	5.87 (5.64 to 6.10)	9.28 (8.72 to 9.84)	8.59 (8.09 to 9.08)	8.82 (8.32 to 9.31)	**0.79 (0.70 to 0.88)**	**1.06 (0.90 to 1.21)**	0.15 (-0.10 to 0.39)
	7-8	2.93 (2.73 to 3.13)	3.47 (3.30 to 3.64)	4.51 (4.32 to 4.70)	5.63 (5.41 to 5.84)	7.93 (7.45 to 8.40)	7.46 (7.02 to 7.90)	8.57 (8.08 to 9.06)	**0.82 (0.74 to 0.91)**	**0.88 (0.74 to 1.03)**	0.15 (-0.10 to 0.39)
	≥8	3.55 (3.29 to 3.80)	4.25 (4.02 to 4.48)	5.78 (5.50 to 6.06)	7.29 (6.98 to 7.60)	10.30 (9.66 to 10.94)	10.10 (9.47 to 10.74)	11.21 (10.52 to 11.90)	**1.09 (0.97 to 1.20)**	**1.22 (1.01 to 1.43)**	0.13 (-0.10 to 0.37)
Low	<6	22.44 (21.44 to 23.44)	25.17 (24.36 to 25.99)	28.44 (27.63 to 29.26)	29.12 (28.27 to 29.97)	35.02 (33.15 to 36.88)	32.37 (30.79 to 33.96)	36.79 (35.27 to 38.30)	**1.94 (1.52 to 2.35)**	**2.46 (1.90 to 3.01)**	0.52 (-0.17 to 1.21)
	6-7	20.47 (19.52 to 21.42)	22.32 (21.55 to 23.09)	24.30 (23.52 to 25.07)	26.28 (25.43 to 27.13)	30.70 (28.78 to 32.62)	27.97 (26.31 to 29.62)	31.62 (30.03 to 33.20)	**1.68 (1.28 to 2.08)**	**1.48 (0.92 to 2.05)**	-0.33 (-0.90 to 0.23)
	7-8	19.77 (18.87 to 20.68)	21.87 (21.08 to 22.65)	24.26 (23.47 to 25.06)	26.26 (25.42 to 27.11)	27.96 (26.08 to 29.84)	26.19 (24.53 to 27.85)	33.63 (31.88 to 35.38)	**1.90 (1.51 to 2.29)**	**1.88 (1.28 to 2.48)**	-0.33 (-0.90 to 0.23)
	≥8	23.33 (22.42 to 24.24)	25.79 (24.97 to 26.62)	27.56 (26.71 to 28.41)	30.74 (29.84 to 31.64)	32.83 (30.92 to 34.73)	28.39 (26.74 to 30.05)	34.89 (33.20 to 36.58)	**2.13 (1.72 to 2.54)**	**0.96 (0.35 to 1.57)**	**-1.17 (-1.90 to -0.44)**

**Depression counseling**											
Yes	<6	10.51 (8.44 to 12.58)	10.50 (8.98 to 12.01)	11.20 (9.85 to 12.55)	13.44 (11.85 to 15.03)	13.34 (10.66 to 16.02)	13.49 (11.19 to 15.78)	13.16 (10.92 to 15.39)	**0.78 (0.05 to 1.51)**	0.23 (-0.62 to 1.07)	-0.56 (-1.67 to 0.56)
	6-7	5.89 (4.36 to 7.42)	5.71 (4.46 to 6.96)	6.45 (5.33 to 7.57)	7.24 (6.00 to 8.47)	7.68 (5.52 to 9.83)	7.33 (5.37 to 9.30)	5.94 (4.43 to 7.44)	0.42 (-0.13 to 0.97)	-0.21 (-0.81 to 0.39)	0.89 (-0.25 to 2.04)
	7-8	4.42 (3.08 to 5.77)	5.77 (4.48 to 7.06)	6.76 (5.51 to 8.02)	5.21 (4.10 to 6.33)	5.14 (3.44 to 6.83)	4.36 (2.87 to 5.85)	7.04 (5.16 to 8.92)	0.21 (-0.32 to 0.74)	0.20 (-0.42 to 0.83)	0.89 (-0.25 to 2.04)
	≥8	7.31 (5.38 to 9.24)	8.75 (7.07 to 10.43)	8.83 (7.26 to 10.40)	7.52 (6.17 to 8.88)	7.95 (5.86 to 10.05)	7.43 (5.43 to 9.42)	11.37 (8.94 to 13.81)	-0.16 (-0.85 to 0.53)	0.80 (-0.01 to 1.61)	0.96 (-0.10 to 2.02)
No	<6	6.87 (6.58 to 7.17)	8.11 (7.86 to 8.36)	9.42 (9.17 to 9.67)	11.33 (11.04 to 11.63)	13.31 (12.73 to 13.89)	13.88 (13.33 to 14.43)	15.30 (14.74 to 15.86)	**1.29 (1.17 to 1.41)**	**1.26 (1.07 to 1.44)**	-0.03 (-0.25 to 0.19)
	6-7	3.93 (3.78 to 4.09)	4.42 (4.29 to 4.55)	5.20 (5.06 to 5.33)	6.51 (6.34 to 6.67)	7.72 (7.40 to 8.04)	7.72 (7.41 to 8.03)	8.52 (8.20 to 8.84)	**0.73 (0.66 to 0.79)**	**0.64 (0.54 to 0.75)**	**-0.42 (-0.58 to -0.26)**
	7-8	3.40 (3.26 to 3.53)	4.09 (3.97 to 4.21)	4.90 (4.78 to 5.03)	5.94 (5.79 to 6.09)	6.00 (5.74 to 6.25)	6.53 (6.26 to 6.79)	7.57 (7.28 to 7.87)	**0.71 (0.66 to 0.77)**	**0.46 (0.37 to 0.55)**	**-0.42 (-0.58 to -0.26)**
	≥8	5.23 (5.04 to 5.42)	6.14 (5.96 to 6.32)	7.24 (7.04 to 7.44)	8.87 (8.65 to 9.10)	8.74 (8.38 to 9.10)	9.25 (8.86 to 9.63)	10.74 (10.32 to 11.17)	**1.00 (0.91 to 1.08)**	**0.51 (0.38 to 0.64)**	**-0.49 (-0.64 to -0.33)**
**Stress counseling**											
Yes	<6	11.38 (8.62 to 14.14)	12.79 (10.60 to 14.99)	14.42 (12.34 to 16.50)	15.44 (13.33 to 17.54)	14.77 (11.55 to 17.99)	14.75 (11.19 to 18.30)	17.50 (14.31 to 20.69)	0.74 (-0.29 to 1.76)	0.69 (-0.46 to 1.85)	-0.04 (-1.59 to 1.50)
	6-7	7.49 (5.21 to 9.78)	7.87 (5.83 to 9.91)	9.18 (7.33 to 11.04)	9.45 (7.61 to 11.29)	7.93 (5.28 to 10.58)	9.65 (6.65 to 12.64)	9.48 (6.69 to 12.28)	0.59 (-0.25 to 1.43)	0.05 (-0.93 to 1.03)	1.43 (-0.01 to 2.88)
	7-8	5.28 (3.30 to 7.26)	7.16 (5.06 to 9.25)	9.37 (7.38 to 11.36)	8.19 (6.33 to 10.06)	6.61 (4.22 to 8.99)	8.13 (5.75 to 10.51)	10.48 (7.51 to 13.45)	0.81 (-0.04 to 1.66)	0.51 (-0.56 to 1.59)	1.43 (-0.01 to 2.88)
	≥8	8.33 (5.83 to 10.82)	11.85 (9.39 to 14.30)	12.32 (10.11 to 14.52)	9.25 (7.34 to 11.17)	9.55 (6.47 to 12.63)	9.52 (6.87 to 12.18)	16.94 (13.53 to 20.36)	0.03 (-0.89 to 0.94)	**1.79 (0.67 to 2.91)**	**1.77 (0.32 to 3.21)**
No	<6	6.90 (6.61 to 7.19)	8.10 (7.85 to 8.34)	9.39 (9.14 to 9.64)	11.32 (11.03 to 11.61)	13.26 (12.68 to 13.84)	13.83 (13.29 to 14.37)	15.11 (14.56 to 15.65)	**1.28 (1.16 to 1.40)**	**1.21 (1.03 to 1.40)**	-0.07 (-0.28 to 0.15)
	6-7	3.93 (3.78 to 4.09)	4.41 (4.29 to 4.54)	5.18 (5.05 to 5.32)	6.49 (6.33 to 6.66)	7.72 (7.39 to 8.04)	7.69 (7.38 to 7.99)	8.43 (8.12 to 8.75)	**0.72 (0.66 to 0.79)**	**0.63 (0.53 to 0.73)**	**-0.43 (-0.59 to -0.27)**

	7-8	3.40 (3.26 to 3.53)	4.09 (3.97 to 4.21)	4.90 (4.77 to 5.03)	5.91 (5.76 to 6.06)	5.97 (5.72 to 6.23)	6.46 (6.20 to 6.72)	7.52 (7.23 to 7.82)	**0.70 (0.65 to 0.76)**	**0.45 (0.36 to 0.54)**	**-0.43 (-0.59 to -0.27)**
	≥8	5.22 (5.03 to 5.41)	6.12 (5.94 to 6.30)	7.21 (7.01 to 7.40)	8.84 (8.62 to 9.06)	8.71 (8.35 to 9.07)	9.19 (8.80 to 9.57)	10.62 (10.20 to 11.05)	**0.99 (0.90 to 1.07)**	**0.49 (0.36 to 0.62)**	**-0.50 (-0.65 to -0.35)**
**Level of education**											
High school or lower education	<6	14.27 (13.62 to 14.93)	17.65 (17.07 to 18.23)	22.18 (21.56 to 22.81)	25.13 (24.44 to 25.82)	26.81 (25.54 to 28.09)	28.28 (27.04 to 29.51)	31.54 (30.32 to 32.77)	**3.68 (3.38 to 3.99)**	**1.98 (1.54 to 2.43)**	**-1.70 (-2.24 to -1.16)**
	6-7	11.33 (10.81 to 11.85)	14.08 (13.60 to 14.56)	17.94 (17.39 to 18.50)	21.98 (21.33 to 22.62)	22.24 (21.12 to 23.35)	23.32 (22.15 to 24.49)	26.23 (25.06 to 27.40)	**3.55 (3.29 to 3.81)**	**1.32 (0.89 to 1.75)**	**-1.34 (-1.74 to -0.94)**
	7-8	10.53 (10.06 to 11.00)	14.11 (13.63 to 14.59)	17.60 (17.05 to 18.14)	21.22 (20.61 to 21.84)	21.55 (20.46 to 22.65)	23.74 (22.56 to 24.92)	27.22 (25.94 to 28.50)	**3.51 (3.26 to 3.75)**	**1.83 (1.39 to 2.27)**	**-1.34 (-1.74 to -0.94)**
	≥8	13.82 (13.27 to 14.37)	17.49 (16.94 to 18.03)	21.64 (21.01 to 22.27)	25.99 (25.29 to 26.68)	26.62 (25.42 to 27.82)	28.23 (26.93 to 29.52)	30.37 (29.09 to 31.65)	**4.04 (3.76 to 4.33)**	**1.47 (1.01 to 1.94)**	**-2.57 (-3.12 to -2.03)**
College or higher education	<6	3.10 (2.84 to 3.35)	3.76 (3.54 to 3.97)	4.35 (4.14 to 4.56)	5.70 (5.44 to 5.96)	7.29 (6.74 to 7.84)	7.83 (7.32 to 8.35)	8.21 (7.70 to 8.72)	**0.78 (0.67 to 0.89)**	**0.85 (0.69 to 1.02)**	0.07 (-0.13 to 0.27)
	6-7	1.95 (1.82 to 2.08)	2.27 (2.17 to 2.38)	2.82 (2.70 to 2.93)	3.54 (3.40 to 3.68)	4.57 (4.28 to 4.85)	4.82 (4.55 to 5.09)	5.19 (4.91 to 5.47)	**0.48 (0.42 to 0.53)**	**0.55 (0.46 to 0.64)**	-0.14 (-0.29 to 0.02)
	7-8	1.72 (1.61 to 1.83)	2.07 (1.97 to 2.17)	2.71 (2.60 to 2.82)	3.30 (3.17 to 3.42)	3.43 (3.22 to 3.64)	4.03 (3.80 to 4.27)	4.67 (4.42 to 4.92)	**0.49 (0.44 to 0.54)**	**0.40 (0.32 to 0.47)**	-0.14 (-0.29 to 0.02)
	≥8	2.32 (2.16 to 2.47)	2.72 (2.58 to 2.86)	3.38 (3.22 to 3.54)	4.31 (4.12 to 4.49)	4.41 (4.12 to 4.71)	5.03 (4.70 to 5.35)	6.14 (5.76 to 6.52)	**0.57 (0.50 to 0.64)**	**0.50 (0.38 to 0.61)**	-0.07 (-0.20 to 0.06)
**Household income**											
Lowest and second quartile	<6	3.84 (3.49 to 4.19)	4.34 (4.07 to 4.61)	4.49 (4.23 to 4.75)	5.85 (5.56 to 6.15)	7.31 (6.69 to 7.94)	7.92 (7.33 to 8.50)	8.79 (8.20 to 9.37)	**0.61 (0.48 to 0.75)**	**0.87 (0.67 to 1.06)**	**0.25 (0.02 to 0.49)**
	6-7	2.27 (2.10 to 2.44)	2.46 (2.33 to 2.59)	2.80 (2.66 to 2.93)	3.73 (3.57 to 3.89)	4.11 (3.81 to 4.41)	4.87 (4.55 to 5.18)	5.23 (4.93 to 5.54)	**0.44 (0.37 to 0.51)**	**0.49 (0.39 to 0.59)**	0.08 (-0.10 to 0.26)
	7-8	1.96 (1.81 to 2.11)	2.20 (2.08 to 2.32)	2.57 (2.44 to 2.69)	3.22 (3.08 to 3.36)	3.57 (3.33 to 3.82)	3.78 (3.52 to 4.03)	4.69 (4.41 to 4.97)	**0.39 (0.33 to 0.45)**	**0.40 (0.31 to 0.48)**	0.08 (-0.10 to 0.26)
	≥8	3.11 (2.88 to 3.34)	3.10 (2.92 to 3.28)	3.58 (3.38 to 3.79)	4.32 (4.11 to 4.53)	4.63 (4.28 to 4.99)	4.78 (4.42 to 5.14)	6.19 (5.76 to 6.62)	**0.37 (0.28 to 0.46)**	**0.44 (0.32 to 0.57)**	0.08 (-0.08 to 0.24)

Third and highest quartile	<6	9.33 (8.90 to 9.76)	12.17 (11.76 to 12.58)	14.48 (14.07 to 14.89)	18.62 (18.11 to 19.14)	20.42 (19.46 to 21.37)	20.98 (20.07 to 21.90)	24.01 (23.06 to 24.97)	**2.76 (2.56 to 2.97)**	**1.76 (1.43 to 2.09)**	**-1.00 (-1.39 to -0.62)**
	6-7	5.85 (5.59 to 6.11)	7.60 (7.35 to 7.86)	8.93 (8.67 to 9.20)	12.59 (12.21 to 12.97)	15.03 (14.30 to 15.76)	14.13 (13.44 to 14.81)	16.56 (15.80 to 17.32)	**1.84 (1.71 to 1.97)**	**1.25 (1.01 to 1.50)**	**-1.15 (-1.43 to -0.87)**
	7-8	5.00 (4.78 to 5.22)	7.21 (6.97 to 7.46)	8.49 (8.23 to 8.74)	12.09 (11.73 to 12.44)	11.53 (10.95 to 12.12)	12.96 (12.33 to 13.59)	15.68 (14.93 to 16.43)	**1.90 (1.79 to 2.01)**	**0.96 (0.74 to 1.19)**	**-1.15 (-1.43 to -0.87)**
	≥8	7.05 (6.77 to 7.33)	9.75 (9.43 to 10.06)	11.31 (10.98 to 11.65)	15.95 (15.50 to 16.39)	15.38 (14.67 to 16.10)	16.71 (15.92 to 17.51)	19.30 (18.44 to 20.15)	**2.43 (2.28 to 2.57)**	**1.02 (0.74 to 1.30)**	**-1.41 (-1.72 to -1.09)**
**Smoking status**											
Smoker	<6	4.46 (3.99 to 4.93)	4.97 (4.56 to 5.38)	6.05 (5.61 to 6.50)	7.62 (7.07 to 8.16)	10.60 (9.42 to 11.78)	11.57 (10.37 to 12.76)	12.11 (10.89 to 13.33)	**0.97 (0.77 to 1.18)**	**1.54 (1.15 to 1.92)**	**0.57 (0.13 to 1.00)**
	6-7	2.81 (2.56 to 3.06)	3.17 (2.94 to 3.40)	3.97 (3.72 to 4.22)	5.20 (4.87 to 5.53)	7.41 (6.67 to 8.14)	7.33 (6.62 to 8.03)	8.21 (7.48 to 8.94)	**0.71 (0.59 to 0.83)**	**1.03 (0.79 to 1.26)**	-0.05 (-0.42 to 0.33)
	7-8	2.80 (2.56 to 3.04)	3.41 (3.17 to 3.64)	4.30 (4.03 to 4.58)	5.20 (4.87 to 5.52)	5.47 (4.89 to 6.04)	5.88 (5.25 to 6.50)	6.85 (6.18 to 7.51)	**0.71 (0.59 to 0.82)**	**0.42 (0.22 to 0.62)**	-0.05 (-0.42 to 0.33)
	≥8	4.27 (3.93 to 4.62)	4.97 (4.62 to 5.32)	6.33 (5.91 to 6.74)	7.11 (6.63 to 7.59)	7.60 (6.81 to 8.38)	8.63 (7.74 to 9.53)	9.80 (8.86 to 10.75)	**0.83 (0.67 to 1.00)**	**0.84 (0.55 to 1.14)**	0.01 (-0.32 to 0.35)
Ex-smoker	<6	10.51 (9.55 to 11.47)	12.03 (11.26 to 12.80)	14.22 (13.45 to 14.98)	16.24 (15.40 to 17.07)	16.55 (15.06 to 18.04)	18.16 (16.71 to 19.60)	19.21 (17.90 to 20.53)	**1.75 (1.37 to 2.12)**	**1.04 (0.57 to 1.52)**	**-0.70 (-1.31 to -0.09)**
	6-7	7.06 (6.49 to 7.63)	7.85 (7.42 to 8.28)	8.76 (8.33 to 9.18)	10.48 (9.99 to 10.96)	11.10 (10.19 to 12.00)	11.12 (10.27 to 11.97)	12.78 (11.93 to 13.64)	**0.99 (0.78 to 1.20)**	**0.68 (0.40 to 0.97)**	**-1.65 (-2.10 to -1.19)**
	7-8	7.00 (6.47 to 7.53)	7.82 (7.40 to 8.25)	9.72 (9.27 to 10.17)	10.84 (10.35 to 11.33)	9.86 (9.07 to 10.65)	11.54 (10.70 to 12.38)	12.68 (11.85 to 13.52)	**1.22 (1.02 to 1.43)**	**0.54 (0.26 to 0.82)**	**-1.65 (-2.10 to -1.19)**
	≥8	10.89 (10.13 to 11.66)	12.62 (11.96 to 13.27)	15.11 (14.41 to 15.81)	17.64 (16.90 to 18.37)	15.84 (14.71 to 16.98)	17.09 (15.88 to 18.31)	18.20 (17.03 to 19.36)	**2.09 (1.77 to 2.40)**	0.06 (-0.35 to 0.47)	**-2.02 (-2.54 to -1.51)**
Non-smoker	<6	7.26 (6.88 to 7.63)	8.48 (8.17 to 8.79)	9.52 (9.22 to 9.83)	11.37 (11.01 to 11.72)	13.22 (12.53 to 13.91)	13.37 (12.73 to 14.01)	14.77 (14.11 to 15.44)	**1.14 (0.99 to 1.28)**	**1.03 (0.81 to 1.24)**	-0.11 (-0.37 to 0.15)

	6-7	3.77 (3.59 to 3.96)	4.11 (3.96 to 4.26)	4.77 (4.61 to 4.93)	5.89 (5.70 to 6.09)	6.93 (6.56 to 7.30)	6.89 (6.53 to 7.25)	7.12 (6.76 to 7.48)	**0.57 (0.50 to 0.65)**	**0.41 (0.30 to 0.52)**	-0.13 (-0.31 to 0.05)
	7-8	2.95 (2.80 to 3.09)	3.54 (3.40 to 3.67)	4.00 (3.86 to 4.14)	4.95 (4.78 to 5.11)	5.18 (4.90 to 5.46)	5.43 (5.14 to 5.72)	6.24 (5.92 to 6.57)	**0.51 (0.45 to 0.57)**	**0.38 (0.28 to 0.48)**	-0.13 (-0.31 to 0.05)
	≥8	4.55 (4.34 to 4.77)	5.23 (5.03 to 5.43)	5.82 (5.60 to 6.03)	7.22 (6.98 to 7.47)	7.39 (6.99 to 7.79)	7.46 (7.06 to 7.87)	8.78 (8.30 to 9.26)	**0.65 (0.56 to 0.74)**	**0.40 (0.26 to 0.54)**	**-0.25 (-0.42 to -0.09)**
**Alcohol consumption**											
Non-drinker and 1day/month	<6	9.06 (8.63 to 9.49)	10.89 (10.52 to 11.26)	12.62 (12.24 to 12.99)	14.64 (14.22 to 15.05)	15.69 (14.95 to 16.42)	16.04 (15.35 to 16.74)	18.10 (17.38 to 18.81)	**1.61 (1.43 to 1.78)**	**1.00 (0.75 to 1.24)**	**-0.61 (-0.91 to -0.31)**
	6-7	5.12 (4.89 to 5.36)	5.92 (5.71 to 6.12)	6.96 (6.74 to 7.17)	8.58 (8.33 to 8.84)	9.24 (8.79 to 9.69)	9.30 (8.87 to 9.73)	9.93 (9.50 to 10.36)	**0.95 (0.85 to 1.05)**	**0.46 (0.32 to 0.61)**	**-0.88 (-1.10 to -0.66)**
	7-8	4.19 (4.00 to 4.39)	5.23 (5.05 to 5.41)	6.35 (6.15 to 6.56)	7.70 (7.47 to 7.93)	7.39 (7.03 to 7.75)	7.81 (7.45 to 8.18)	9.23 (8.81 to 9.66)	**0.98 (0.89 to 1.06)**	**0.41 (0.28 to 0.54)**	**-0.88 (-1.10 to -0.66)**
	≥8	6.49 (6.22 to 6.76)	7.79 (7.53 to 8.05)	9.22 (8.93 to 9.51)	11.45 (11.12 to 11.78)	10.57 (10.08 to 11.06)	10.82 (10.31 to 11.32)	12.97 (12.40 to 13.54)	**1.37 (1.25 to 1.49)**	**0.32 (0.14 to 0.50)**	**-1.04 (-1.26 to -0.83)**
2–9 days/month	<6	3.40 (3.05 to 3.76)	4.10 (3.80 to 4.40)	4.73 (4.44 to 5.03)	6.17 (5.79 to 6.56)	8.16 (7.28 to 9.04)	8.70 (7.86 to 9.54)	8.70 (7.91 to 9.50)	**0.81 (0.66 to 0.96)**	**0.95 (0.70 to 1.21)**	0.14 (-0.16 to 0.44)
	6-7	2.23 (2.05 to 2.41)	2.49 (2.34 to 2.64)	3.06 (2.90 to 3.21)	3.72 (3.53 to 3.91)	4.93 (4.50 to 5.36)	4.72 (4.31 to 5.14)	5.77 (5.34 to 6.21)	**0.48 (0.40 to 0.56)**	**0.59 (0.45 to 0.73)**	-0.01 (-0.23 to 0.20)
	7-8	2.07 (1.90 to 2.23)	2.44 (2.29 to 2.58)	2.90 (2.75 to 3.05)	3.47 (3.29 to 3.64)	3.58 (3.26 to 3.89)	3.93 (3.57 to 4.29)	4.69 (4.32 to 5.06)	**0.42 (0.35 to 0.49)**	**0.32 (0.21 to 0.43)**	-0.01 (-0.23 to 0.20)
	≥8	2.73 (2.51 to 2.96)	3.15 (2.94 to 3.35)	3.74 (3.51 to 3.97)	4.44 (4.18 to 4.70)	4.62 (4.16 to 5.08)	5.05 (4.54 to 5.55)	5.98 (5.43 to 6.53)	**0.47 (0.37 to 0.57)**	**0.46 (0.29 to 0.62)**	-0.01 (-0.20 to 0.18)
Over 10 days/month	<6	6.99 (5.97 to 8.00)	7.59 (6.79 to 8.40)	10.31 (9.40 to 11.21)	10.64 (9.67 to 11.60)	12.11 (9.97 to 14.25)	12.86 (10.74 to 14.98)	14.93 (12.80 to 17.07)	**1.31 (0.89 to 1.73)**	**1.44 (0.71 to 2.16)**	0.13 (-0.71 to 0.97)
	6-7	5.75 (5.01 to 6.49)	6.19 (5.59 to 6.79)	7.09 (6.50 to 7.69)	8.64 (7.91 to 9.38)	10.04 (8.36 to 11.72)	9.75 (8.11 to 11.39)	11.26 (9.65 to 12.87)	**0.81 (0.51 to 1.12)**	**0.90 (0.38 to 1.42)**	-0.41 (-1.09 to 0.27)
	7-8	5.62 (4.89 to 6.34)	6.62 (6.00 to 7.24)	8.22 (7.56 to 8.87)	8.52 (7.79 to 9.26)	7.99 (6.55 to 9.43)	8.96 (7.45 to 10.48)	11.40 (9.77 to 13.03)	**0.93 (0.63 to 1.24)**	**0.69 (0.19 to 1.20)**	-0.41 (-1.09 to 0.27)
	≥8	7.93 (7.02 to 8.85)	8.92 (8.13 to 9.71)	11.05 (10.15 to 11.94)	11.49 (10.53 to 12.45)	11.95 (10.19 to 13.72)	14.35 (12.19 to 16.52)	14.68 (12.59 to 16.76)	**1.20 (0.80 to 1.61)**	**0.89 (0.23 to 1.55)**	-0.31 (-1.09 to 0.46)

Abbreviations: BMI, body mass index; CI, confidence interval; KCHS, Korea Community Health Survey.

β values indicate the average annual change (%) in the weighted prevalence, multiplied by 100 for interpretability.

The values in bold font represent significant variance ($p<0.05$).

[a] According to the Asian-Pacific guidelines, BMI is divided into four groups: underweight (<18.5 kg/ m^2), normal (18.5–22.9 kg/ m^2), overweight (23.0–24.9 kg/ m^2), and obese (≥25.0 kg/ m^2).

Table S5. Weighted odds ratios in the prevalence of individuals with hypertension stratified by sleep duration before and during COVID–19, with 7–8hours of sleep duration as the reference (weighted % [95% CI]).

Variables	Sleep duration (hours/day)	Overall (2009–2022)		Pre-pandemic era (2009–2019)		During pandemic era (2020–2022)	
		Weighted OR (95% CI)	p-value	Weighted OR (95% CI)	p-value	Weighted OR (95% CI)	p-value
Overall	7-8 (ref)	1.00 (reference)		1.00 (reference)		1.00 (reference)	
	< 6	**1.23 (1.21 to 1.24)**	**<0.001**	**1.14 (1.12 to 1.15)**	**<0.001**	**1.18 (1.15 to 1.22)**	**<0.001**
	6-7	**1.07 (1.06 to 1.08)**	**<0.001**	**0.93 (0.92 to 0.95)**	**<0.001**	**0.93 (0.91 to 0.96)**	**<0.001**
	≥ 8	**1.05 (1.04 to 1.06)**	**<0.001**	**0.98 (0.97 to 0.99)**	**0.004**	0.98 (0.96 to 1.01)	0.173
Sex							
Male	7-8 (ref)	1.00 (reference)		1.00 (reference)		1.00 (reference)	
	< 6	**1.20 (1.18 to 1.22)**	**<0.001**	**1.12 (1.10 to 1.14)**	**<0.001**	**1.13 (1.09 to 1.18)**	**<0.001**
	6-7	**1.07 (1.05 to 1.09)**	**<0.001**	**0.94 (0.92 to 0.96)**	**<0.001**	**0.92 (0.89 to 0.95)**	**<0.001**
	≥ 8	**1.08 (1.06 to 1.09)**	**<0.001**	1.01 (0.99 to 1.02)	0.606	1.01 (0.97 to 1.05)	0.580
Female	7-8 (ref)	1.00 (reference)		1.00 (reference)		1.00 (reference)	
	< 6	**1.18 (1.16 to 1.20)**	**<0.001**	**1.12 (1.10 to 1.14)**	**<0.001**	**1.20 (1.16 to 1.24)**	**<0.001**
	6-7	**1.04 (1.02 to 1.05)**	**<0.001**	**0.96 (0.94 to 0.98)**	**<0.001**	0.98 (0.95 to 1.01)	0.207
	≥ 8	**1.07 (1.05 to 1.09)**	**<0.001**	**1.03 (1.01 to 1.05)**	**0.001**	1.03 (0.99 to 1.07)	0.159
Age, years							
19–30	7-8 (ref)	1.00 (reference)		1.00 (reference)		1.00 (reference)	

	< 6	**1.71 (1.54 to 1.89)**	**<0.001**	**1.39 (1.24 to 1.56)**	**<0.001**	**1.40 (1.08 to 1.81)**	**0.011**
	6-7	**1.23 (1.13 to 1.34)**	**<0.001**	**0.86 (0.78 to 0.94)**	**0.001**	**0.64 (0.53 to 0.78)**	**<0.001**
	≥ 8	**1.10 (1.01 to 1.20)**	**0.036**	0.93 (0.84 to 1.03)	0.166	**0.75 (0.61 to 0.92)**	**0.006**
31–40	7-8 (ref)	1.00 (reference)		1.00 (reference)		1.00 (reference)	
	< 6	**1.46 (1.37 to 1.55)**	**<0.001**	**1.28 (1.20 to 1.36)**	**<0.001**	**1.37 (1.19 to 1.59)**	**<0.001**
	6-7	**1.13 (1.08 to 1.19)**	**<0.001**	**0.87 (0.83 to 0.92)**	**<0.001**	0.92 (0.82 to 1.04)	0.184
	≥ 8	1.02 (0.97 to 1.09)	0.442	**0.90 (0.85 to 0.96)**	**0.002**	0.91 (0.79 to 1.05)	0.189
41–50	7-8 (ref)	1.00 (reference)		1.00 (reference)		1.00 (reference)	
	< 6	**1.31 (1.27 to 1.36)**	**<0.001**	**1.18 (1.14 to 1.23)**	**<0.001**	**1.20 (1.11 to 1.30)**	**<0.001**
	6-7	**1.11 (1.08 to 1.14)**	**<0.001**	**0.91 (0.88 to 0.94)**	**<0.001**	**0.88 (0.82 to 0.93)**	**<0.001**
	≥ 8	**1.09 (1.05 to 1.13)**	**<0.001**	0.99 (0.96 to 1.03)	0.681	0.94 (0.87 to 1.02)	0.145
51–60	7-8 (ref)	1.00 (reference)		1.00 (reference)		1.00 (reference)	
	< 6	**1.21 (1.18 to 1.23)**	**<0.001**	**1.14 (1.11 to 1.17)**	**<0.001**	**1.20 (1.13 to 1.26)**	**<0.001**
	6-7	**1.05 (1.02 to 1.07)**	**<0.001**	**0.96 (0.94 to 0.98)**	**0.001**	**0.94 (0.90 to 0.99)**	**0.013**
	≥ 8	**1.13 (1.10 to 1.16)**	**<0.001**	**1.09 (1.06 to 1.12)**	**<0.001**	1.05 (1.00 to 1.11)	0.055
61–70	7-8 (ref)	1.00 (reference)		1.00 (reference)		1.00 (reference)	
	< 6	**1.14 (1.11 to 1.16)**	**<0.001**	**1.09 (1.06 to 1.12)**	**<0.001**	**1.17 (1.12 to 1.23)**	**<0.001**
	6-7	**1.02 (1.00 to 1.05)**	**0.029**	**0.98 (0.95 to 1.00)**	**0.038**	0.98 (0.94 to 1.03)	0.397
	≥ 8	**1.08 (1.06 to 1.11)**	**<0.001**	**1.05 (1.03 to 1.08)**	**<0.001**	**1.08 (1.03 to 1.13)**	**0.002**
>70	7-8 (ref)	1.00 (reference)		1.00 (reference)		1.00 (reference)	
	< 6	**1.07 (1.05 to 1.10)**	**<0.001**	**1.05 (1.02 to 1.08)**	**<0.001**	**1.11 (1.06 to 1.16)**	**<0.001**

	6-7	1.01 (0.98 to 1.03)	0.601	0.99 (0.96 to 1.02)	0.395	1.01 (0.96 to 1.06)	0.710
	≥ 8	0.98 (0.96 to 1.00)	0.061	**0.96 (0.94 to 0.99)**	**0.002**	1.01 (0.97 to 1.06)	0.597
Region of residence							
Urban	7-8 (ref)	1.00 (reference)		1.00 (reference)		1.00 (reference)	
	< 6	**1.25 (1.23 to 1.27)**	**<0.001**	**1.15 (1.13 to 1.17)**	**<0.001**	**1.20 (1.16 to 1.23)**	**<0.001**
	6-7	**1.08 (1.06 to 1.09)**	**<0.001**	**0.93 (0.92 to 0.95)**	**<0.001**	**0.92 (0.90 to 0.95)**	**<0.001**
	≥ 8	**1.06 (1.04 to 1.07)**	**<0.001**	**0.98 (0.97 to 1.00)**	**0.036**	0.98 (0.95 to 1.01)	0.140
Rural	7-8 (ref)	1.00 (reference)		1.00 (reference)		1.00 (reference)	
	< 6	**1.16 (1.14 to 1.19)**	**<0.001**	**1.09 (1.07 to 1.11)**	**<0.001**	**1.13 (1.09 to 1.18)**	**<0.001**
	6-7	**1.06 (1.04 to 1.08)**	**<0.001**	**0.94 (0.92 to 0.96)**	**<0.001**	0.97 (0.93 to 1.01)	0.100
	≥ 8	**1.04 (1.02 to 1.06)**	**<0.001**	**0.98 (0.96 to 1.00)**	**0.029**	0.99 (0.95 to 1.03)	0.702
BMI group							
Underweight	7-8 (ref)	1.00 (reference)		1.00 (reference)		1.00 (reference)	
	< 6	**1.15 (1.10 to 1.21)**	**<0.001**	**1.09 (1.04 to 1.14)**	**0.001**	**1.30 (1.14 to 1.48)**	**<0.001**
	6-7	1.03 (0.99 to 1.08)	0.157	0.96 (0.91 to 1.00)	0.063	1.06 (0.92 to 1.23)	0.401
	≥ 8	**1.08 (1.04 to 1.13)**	**<0.001**	1.02 (0.98 to 1.07)	0.342	**1.21 (1.06 to 1.38)**	**0.006**
Normal weight	7-8 (ref)	1.00 (reference)		1.00 (reference)		1.00 (reference)	
	< 6	**1.22 (1.19 to 1.25)**	**<0.001**	**1.15 (1.12 to 1.18)**	**<0.001**	**1.20 (1.15 to 1.25)**	**<0.001**
	6-7	**1.05 (1.03 to 1.07)**	**<0.001**	**0.95 (0.93 to 0.97)**	**<0.001**	**0.96 (0.92 to 1.00)**	**0.048**
	≥ 8	**1.08 (1.05 to 1.10)**	**<0.001**	1.02 (1.00 to 1.05)	0.053	1.04 (0.99 to 1.09)	0.113

Overweight	7-8 (ref)	1.00 (reference)		1.00 (reference)		1.00 (reference)	
	< 6	**1.15 (1.12 to 1.18)**	**<0.001**	**1.10 (1.07 to 1.13)**	**<0.001**	**1.18 (1.12 to 1.24)**	**<0.001**
	6-7	**1.03 (1.01 to 1.05)**	**0.009**	**0.97 (0.94 to 0.99)**	**0.006**	0.99 (0.95 to 1.04)	0.685
	≥ 8	**1.05 (1.02 to 1.07)**	**<0.001**	1.01 (0.99 to 1.04)	0.349	1.04 (0.98 to 1.09)	0.177
Obese	7-8 (ref)	1.00 (reference)		1.00 (reference)		1.00 (reference)	
	< 6	**1.19 (1.17 to 1.22)**	**<0.001**	**1.13 (1.10 to 1.15)**	**<0.001**	**1.13 (1.08 to 1.18)**	**<0.001**
	6-7	**1.06 (1.04 to 1.08)**	**<0.001**	**0.95 (0.93 to 0.97)**	**<0.001**	**0.92 (0.89 to 0.96)**	**<0.001**
	≥ 8	**1.06 (1.04 to 1.08)**	**<0.001**	1.01 (0.98 to 1.03)	0.506	0.98 (0.93 to 1.02)	0.285
Subjective Health-level							
High (ref)	7-8 (ref)	1.00 (reference)		1.00 (reference)		1.00 (reference)	
	< 6	**1.17 (1.14 to 1.20)**	**<0.001**	**1.10 (1.07 to 1.13)**	**<0.001**	**1.09 (1.04 to 1.15)**	**<0.001**
	6-7	**1.07 (1.04 to 1.09)**	**<0.001**	**0.94 (0.92 to 0.96)**	**<0.001**	**0.93 (0.90 to 0.97)**	**0.001**
	≥ 8	**1.03 (1.00 to 1.05)**	**0.040**	**0.96 (0.93 to 0.98)**	**0.002**	0.97 (0.93 to 1.02)	0.193
Middle	7-8 (ref)	1.00 (reference)		1.00 (reference)		1.00 (reference)	
	< 6	**1.15 (1.13 to 1.17)**	**<0.001**	**1.07 (1.05 to 1.10)**	**<0.001**	**1.12 (1.08 to 1.16)**	**<0.001**
	6-7	**1.06 (1.04 to 1.08)**	**<0.001**	**0.94 (0.93 to 0.96)**	**<0.001**	**0.95 (0.91 to 0.98)**	**0.002**
	≥ 8	1.02 (1.00 to 1.04)	0.085	**0.96 (0.94 to 0.98)**	**<0.001**	0.98 (0.94 to 1.02)	0.245
Low	7-8 (ref)	1.00 (reference)		1.00 (reference)		1.00 (reference)	
	< 6	**1.05 (1.02 to 1.07)**	**<0.001**	1.01 (0.98 to 1.03)	0.572	**1.09 (1.04 to 1.15)**	**0.001**
	6-7	1.02 (1.00 to 1.05)	0.051	**0.97 (0.94 to 0.99)**	**0.007**	1.04 (0.98 to 1.10)	0.208

	≥ 8	**0.96 (0.94 to 0.99)**	**0.001**	**0.94 (0.92 to 0.96)**	**<0.001**	0.96 (0.90 to 1.01)	0.111
Depression counseling							
Yes	7-8 (ref)	1.00 (reference)		1.00 (reference)		1.00 (reference)	
	< 6	**1.25 (1.13 to 1.38)**	**<0.001**	**1.23 (1.11 to 1.37)**	**<0.001**	**1.33 (1.13 to 1.57)**	**0.001**
	6-7	0.99 (0.89 to 1.10)	0.814	1.01 (0.89 to 1.15)	0.868	1.01 (0.84 to 1.23)	0.890
	≥ 8	1.03 (0.92 to 1.15)	0.596	1.05 (0.93 to 1.18)	0.462	1.04 (0.87 to 1.24)	0.698
No	7-8 (ref)	1.00 (reference)		1.00 (reference)		1.00 (reference)	
	< 6	**1.22 (1.21 to 1.24)**	**<0.001**	**1.13 (1.11 to 1.15)**	**<0.001**	**1.17 (1.14 to 1.21)**	**<0.001**
	6-7	**1.07 (1.06 to 1.08)**	**<0.001**	**0.93 (0.92 to 0.95)**	**<0.001**	**0.93 (0.91 to 0.96)**	**<0.001**
	≥ 8	**1.05 (1.04 to 1.06)**	**<0.001**	**0.98 (0.97 to 0.99)**	**0.001**	0.98 (0.95 to 1.01)	0.121
Stress counseling							
Yes	7-8 (ref)	1.00 (reference)		1.00 (reference)		1.00 (reference)	
	< 6	**1.29 (1.19 to 1.40)**	**<0.001**	**1.23 (1.13 to 1.34)**	**<0.001**	**1.25 (1.09 to 1.43)**	**0.002**
	6-7	1.05 (0.96 to 1.14)	0.299	0.96 (0.87 to 1.07)	0.472	0.93 (0.79 to 1.09)	0.370
	≥ 8	1.08 (0.98 to 1.19)	0.109	1.07 (0.96 to 1.18)	0.219	0.94 (0.80 to 1.10)	0.440
No	7-8 (ref)	1.00 (reference)		1.00 (reference)		1.00 (reference)	
	< 6	**1.22 (1.20 to 1.23)**	**<0.001**	**1.13 (1.11 to 1.14)**	**<0.001**	**1.17 (1.14 to 1.20)**	**<0.001**
	6-7	**1.07 (1.06 to 1.08)**	**<0.001**	**0.93 (0.92 to 0.95)**	**<0.001**	**0.94 (0.91 to 0.96)**	**<0.001**
	≥ 8	**1.05 (1.04 to 1.06)**	**<0.001**	**0.98 (0.96 to 0.99)**	**0.001**	0.98 (0.96 to 1.01)	0.188
Education							

High school or lower education	7-8 (ref)	1.00 (reference)		1.00 (reference)		1.00 (reference)	
	< 6	**1.10 (1.09 to 1.12)**	**<0.001**	**1.07 (1.05 to 1.09)**	**<0.001**	**1.13 (1.09 to 1.18)**	**<0.001**
	6-7	**1.02 (1.01 to 1.04)**	**<0.001**	**0.97 (0.96 to 0.99)**	**0.005**	1.00 (0.96 to 1.04)	0.941
	≥ 8	**1.03 (1.02 to 1.05)**	**<0.001**	1.00 (0.99 to 1.02)	0.653	**1.05 (1.01 to 1.09)**	**0.015**
College or higher education	7-8 (ref)	1.00 (reference)		1.00 (reference)		1.00 (reference)	
	< 6	**1.28 (1.25 to 1.30)**	**<0.001**	**1.17 (1.15 to 1.20)**	**<0.001**	**1.18 (1.14 to 1.23)**	**<0.001**
	6-7	**1.09 (1.07 to 1.10)**	**<0.001**	**0.92 (0.91 to 0.94)**	**<0.001**	**0.92 (0.89 to 0.94)**	**<0.001**
	≥ 8	**1.06 (1.04 to 1.07)**	**<0.001**	**0.98 (0.96 to 0.99)**	**0.011**	**0.96 (0.92 to 0.99)**	**0.015**
Household income							
Lowest and second quartile	7-8 (ref)	1.00 (reference)		1.00 (reference)		1.00 (reference)	
	< 6	**1.27 (1.25 to 1.30)**	**<0.001**	**1.16 (1.13 to 1.19)**	**<0.001**	**1.19 (1.14 to 1.23)**	**<0.001**
	6-7	**1.09 (1.07 to 1.11)**	**<0.001**	**0.91 (0.90 to 0.93)**	**<0.001**	**0.92 (0.89 to 0.95)**	**<0.001**
	≥ 8	**1.06 (1.04 to 1.08)**	**<0.001**	**0.97 (0.95 to 0.99)**	**0.008**	0.98 (0.94 to 1.01)	0.214
Third and highest quartile	7-8 (ref)	1.00 (reference)		1.00 (reference)		1.00 (reference)	
	< 6	**1.16 (1.15 to 1.18)**	**<0.001**	**1.11 (1.09 to 1.12)**	**<0.001**	**1.16 (1.12 to 1.20)**	**<0.001**
	6-7	**1.04 (1.03 to 1.06)**	**<0.001**	**0.96 (0.94 to 0.98)**	**<0.001**	**0.96 (0.93 to 1.00)**	**0.025**
	≥ 8	**1.04 (1.02 to 1.05)**	**<0.001**	0.99 (0.98 to 1.01)	0.435	1.00 (0.97 to 1.04)	0.968
Smoking status							
Non-smoker	7-8 (ref)	1.00 (reference)		1.00 (reference)		1.00 (reference)	
	< 6	**1.27 (1.23 to 1.31)**	**<0.001**	**1.16 (1.12 to 1.20)**	**<0.001**	**1.17 (1.09 to 1.25)**	**<0.001**

	6-7	**1.09 (1.07 to 1.12)**	**<0.001**	**0.92 (0.90 to 0.95)**	**<0.001**	**0.87 (0.82 to 0.92)**	**<0.001**
	≥ 8	**1.07 (1.04 to 1.10)**	**<0.001**	0.99 (0.96 to 1.02)	0.508	**0.94 (0.88 to 1.00)**	**0.038**
Ex-smoker	7-8 (ref)	1.00 (reference)		1.00 (reference)		1.00 (reference)	
	< 6	**1.16 (1.13 to 1.19)**	**<0.001**	**1.10 (1.07 to 1.14)**	**<0.001**	**1.10 (1.04 to 1.16)**	**0.001**
	6-7	**1.05 (1.03 to 1.08)**	**<0.001**	**0.96 (0.93 to 0.98)**	**0.001**	**0.93 (0.89 to 0.98)**	**0.005**
	≥ 8	**1.06 (1.04 to 1.09)**	**<0.001**	1.00 (0.98 to 1.03)	0.811	1.03 (0.98 to 1.09)	0.234
Smoker	7-8 (ref)	1.00 (reference)		1.00 (reference)		1.00 (reference)	
	< 6	**1.21 (1.19 to 1.22)**	**<0.001**	**1.13 (1.11 to 1.15)**	**<0.001**	**1.20 (1.16 to 1.24)**	**<0.001**
	6-7	**1.05 (1.04 to 1.07)**	**<0.001**	**0.95 (0.93 to 0.96)**	**<0.001**	**0.97 (0.94 to 1.00)**	**0.042**
	≥ 8	**1.06 (1.05 to 1.08)**	**<0.001**	1.01 (0.99 to 1.03)	0.181	1.01 (0.97 to 1.04)	0.718
Alcohol consumption							
Non-drinker and 1day/month	7-8 (ref)	1.00 (reference)		1.00 (reference)		1.00 (reference)	
	< 6	**1.20 (1.18 to 1.22)**	**<0.001**	**1.13 (1.11 to 1.15)**	**<0.001**	**1.17 (1.14 to 1.21)**	**<0.001**
	6-7	**1.05 (1.03 to 1.06)**	**<0.001**	**0.95 (0.94 to 0.97)**	**<0.001**	**0.95 (0.93 to 0.98)**	**0.002**
	≥ 8	**1.06 (1.04 to 1.07)**	**<0.001**	1.01 (0.99 to 1.03)	0.359	1.00 (0.97 to 1.04)	0.876
2–9 days/month	7-8 (ref)	1.00 (reference)		1.00 (reference)		1.00 (reference)	
	< 6	**1.28 (1.25 to 1.31)**	**<0.001**	**1.16 (1.13 to 1.19)**	**<0.001**	**1.21 (1.15 to 1.28)**	**<0.001**
	6-7	**1.10 (1.08 to 1.12)**	**<0.001**	**0.91 (0.89 to 0.93)**	**<0.001**	**0.90 (0.86 to 0.94)**	**<0.001**
	≥ 8	**1.05 (1.03 to 1.08)**	**<0.001**	**0.96 (0.94 to 0.98)**	**0.001**	0.96 (0.91 to 1.01)	0.096
	7-8 (ref)	1.00 (reference)		1.00 (reference)		1.00 (reference)	

Over 10 days/month	< 6	**1.11 (1.07 to 1.16)**	**<0.001**	**1.07 (1.03 to 1.12)**	**0.002**	**1.16 (1.05 to 1.28)**	**0.005**
	6-7	1.02 (0.99 to 1.06)	0.214	0.98 (0.94 to 1.02)	0.289	0.97 (0.88 to 1.06)	0.460
	≥ 8	**1.06 (1.02 to 1.10)**	**0.007**	1.03 (0.98 to 1.07)	0.252	1.05 (0.95 to 1.16)	0.318

Abbreviations: BMI, body mass index; CI, confidence interval; KCHS, Korea Community Health Survey.

The values in bold font represent significant variance (p<0.05).

[a] According to the Asian-Pacific guidelines, BMI is divided into four groups: underweight (<18.5 kg/ m^2), normal (18.5–22.9 kg/ m^2), overweight (23.0–24.9 kg/ m^2), and obese (≥25.0 kg/ m^2).

Table S6. Weighted odds ratios in the prevalence of individuals with diabetes stratified by sleep duration before and during COVID–19, with 7–8hours of sleep duration as the reference (weighted % [95% CI]).

Variables	Sleep duration (hours/day)	Overall (2009–2022)		Pre-pandemic era (2009–2019)		During pandemic era (2020–2022)	
		Weighted OR (95% CI)	p-value	Weighted OR (95% CI)	p-value	Weighted OR (95% CI)	p-value
Overall	7-8 (ref)	1.00 (reference)		1.00 (reference)		1.00 (reference)	
	< 6	**1.17 (1.15 to 1.19)**	**<0.001**	**1.16 (1.14 to 1.18)**	**<0.001**	**1.18 (1.15 to 1.22)**	**<0.001**
	6-7	1.00 (0.99 to 1.02)	0.656	1.00 (0.98 to 1.02)	0.972	0.98 (0.95 to 1.01)	0.286
	≥ 8	**1.21 (1.19 to 1.23)**	**<0.001**	**1.21 (1.19 to 1.24)**	**<0.001**	**1.18 (1.15 to 1.22)**	**<0.001**
Sex							
Male	7-8 (ref)	1.00 (reference)		1.00 (reference)		1.00 (reference)	
	< 6	**1.16 (1.13 to 1.19)**	**<0.001**	**1.16 (1.13 to 1.19)**	**<0.001**	**1.18 (1.13 to 1.24)**	**<0.001**
	6-7	1.00 (0.98 to 1.02)	0.752	1.01 (0.99 to 1.04)	0.247	0.97 (0.93 to 1.01)	0.121
	≥ 8	**1.22 (1.20 to 1.25)**	**<0.001**	**1.24 (1.21 to 1.27)**	**<0.001**	**1.18 (1.13 to 1.23)**	**<0.001**
Female	7-8 (ref)	1.00 (reference)		1.00 (reference)		1.00 (reference)	
	< 6	**1.14 (1.12 to 1.17)**	**<0.001**	**1.14 (1.11 to 1.17)**	**<0.001**	**1.17 (1.12 to 1.22)**	**<0.001**
	6-7	1.00 (0.98 to 1.02)	0.776	1.00 (0.97 to 1.02)	0.774	1.02 (0.98 to 1.07)	0.333
	≥ 8	**1.22 (1.19 to 1.25)**	**<0.001**	**1.22 (1.19 to 1.25)**	**<0.001**	**1.24 (1.18 to 1.30)**	**<0.001**
Age, years							
19–30	7-8 (ref)	1.00 (reference)		1.00 (reference)		1.00 (reference)	

	< 6	**1.36 (1.10 to 1.69)**	**0.004**	1.16 (0.89 to 1.51)	0.276	**1.62 (1.06 to 2.47)**	**0.026**
	6-7	1.09 (0.91 to 1.30)	0.345	0.93 (0.76 to 1.13)	0.463	0.82 (0.58 to 1.17)	0.278
	≥ 8	**1.23 (1.05 to 1.45)**	**0.010**	1.18 (0.97 to 1.44)	0.107	0.93 (0.66 to 1.30)	0.659
31–40	7-8 (ref)	1.00 (reference)		1.00 (reference)		1.00 (reference)	
	< 6	**1.47 (1.34 to 1.62)**	**<0.001**	**1.50 (1.34 to 1.68)**	**<0.001**	**1.42 (1.15 to 1.76)**	**0.001**
	6-7	0.99 (0.92 to 1.08)	0.867	1.03 (0.94 to 1.13)	0.535	0.90 (0.75 to 1.08)	0.251
	≥ 8	**1.29 (1.18 to 1.40)**	**<0.001**	**1.33 (1.21 to 1.47)**	**<0.001**	1.13 (0.93 to 1.37)	0.229
41–50	7-8 (ref)	1.00 (reference)		1.00 (reference)		1.00 (reference)	
	< 6	**1.37 (1.31 to 1.45)**	**<0.001**	**1.34 (1.26 to 1.42)**	**<0.001**	**1.32 (1.17 to 1.48)**	**<0.001**
	6-7	1.03 (0.99 to 1.08)	0.166	0.97 (0.93 to 1.02)	0.244	0.95 (0.86 to 1.04)	0.281
	≥ 8	**1.27 (1.21 to 1.33)**	**<0.001**	**1.25 (1.18 to 1.32)**	**<0.001**	**1.16 (1.04 to 1.30)**	**0.009**
51–60	7-8 (ref)	1.00 (reference)		1.00 (reference)		1.00 (reference)	
	< 6	**1.25 (1.20 to 1.29)**	**<0.001**	**1.22 (1.17 to 1.27)**	**<0.001**	**1.24 (1.15 to 1.33)**	**<0.001**
	6-7	1.02 (0.99 to 1.05)	0.231	0.99 (0.96 to 1.03)	0.701	0.94 (0.88 to 1.01)	0.069
	≥ 8	**1.31 (1.27 to 1.36)**	**<0.001**	**1.30 (1.25 to 1.34)**	**<0.001**	**1.28 (1.19 to 1.38)**	**<0.001**
61–70	7-8 (ref)	1.00 (reference)		1.00 (reference)		1.00 (reference)	
	< 6	**1.10 (1.07 to 1.13)**	**<0.001**	**1.11 (1.07 to 1.15)**	**<0.001**	**1.18 (1.11 to 1.25)**	**<0.001**
	6-7	**0.97 (0.95 to 1.00)**	**0.049**	1.02 (0.99 to 1.06)	0.171	1.05 (0.99 to 1.10)	0.111
	≥ 8	**1.22 (1.19 to 1.25)**	**<0.001**	**1.25 (1.21 to 1.29)**	**<0.001**	**1.27 (1.20 to 1.34)**	**<0.001**
>70	7-8 (ref)	1.00 (reference)		1.00 (reference)		1.00 (reference)	
	< 6	1.02 (0.99 to 1.05)	0.177	**1.03 (1.00 to 1.07)**	**0.039**	**1.08 (1.03 to 1.14)**	**0.002**

	6-7	**0.97 (0.95 to 1.00)**	**0.047**	1.03 (1.00 to 1.07)	0.066	1.03 (0.97 to 1.08)	0.366
	≥ 8	**1.10 (1.08 to 1.13)**	**<0.001**	**1.14 (1.10 to 1.17)**	**<0.001**	**1.14 (1.09 to 1.20)**	**<0.001**
Region of residence							
Urban	7-8 (ref)	1.00 (reference)		1.00 (reference)		1.00 (reference)	
	< 6	**1.18 (1.16 to 1.21)**	**<0.001**	**1.17 (1.15 to 1.20)**	**<0.001**	**1.20 (1.16 to 1.25)**	**<0.001**
	6-7	1.00 (0.98 to 1.02)	0.796	1.00 (0.98 to 1.02)	0.944	0.98 (0.95 to 1.02)	0.346
	≥ 8	**1.24 (1.21 to 1.26)**	**<0.001**	**1.24 (1.22 to 1.27)**	**<0.001**	**1.20 (1.15 to 1.25)**	**<0.001**
Rural	7-8 (ref)	1.00 (reference)		1.00 (reference)		1.00 (reference)	
	< 6	**1.12 (1.10 to 1.15)**	**<0.001**	**1.11 (1.07 to 1.14)**	**<0.001**	**1.11 (1.05 to 1.17)**	**<0.001**
	6-7	1.01 (0.99 to 1.04)	0.292	0.99 (0.97 to 1.02)	0.451	0.98 (0.94 to 1.03)	0.478
	≥ 8	**1.15 (1.12 to 1.17)**	**<0.001**	**1.14 (1.11 to 1.17)**	**<0.001**	**1.13 (1.08 to 1.19)**	**<0.001**
BMI group							
Underweight	7-8 (ref)	1.00 (reference)		1.00 (reference)		1.00 (reference)	
	< 6	**1.10 (1.04 to 1.17)**	**0.002**	**1.08 (1.01 to 1.16)**	**0.017**	**1.26 (1.07 to 1.50)**	**0.007**
	6-7	1.00 (0.94 to 1.06)	0.926	1.00 (0.93 to 1.07)	0.917	1.06 (0.89 to 1.27)	0.495
	≥ 8	**1.21 (1.14 to 1.29)**	**<0.001**	**1.20 (1.13 to 1.28)**	**<0.001**	**1.34 (1.13 to 1.59)**	**0.001**
Normal weight	7-8 (ref)	1.00 (reference)		1.00 (reference)		1.00 (reference)	
	< 6	**1.19 (1.16 to 1.23)**	**<0.001**	**1.20 (1.16 to 1.24)**	**<0.001**	**1.24 (1.17 to 1.31)**	**<0.001**
	6-7	0.99 (0.96 to 1.01)	0.347	1.01 (0.98 to 1.04)	0.605	1.03 (0.97 to 1.09)	0.304
	≥ 8	**1.20 (1.17 to 1.23)**	**<0.001**	**1.22 (1.18 to 1.26)**	**<0.001**	**1.21 (1.14 to 1.28)**	**<0.001**
Overweight	7-8 (ref)	1.00 (reference)		1.00 (reference)		1.00 (reference)	

	< 6	**1.14 (1.11 to 1.18)**	**<0.001**	**1.14 (1.10 to 1.18)**	**<0.001**	**1.11 (1.04 to 1.18)**	**0.002**
	6-7	1.01 (0.98 to 1.04)	0.508	1.00 (0.97 to 1.04)	0.887	0.95 (0.90 to 1.01)	0.107
	≥ 8	**1.25 (1.21 to 1.29)**	**<0.001**	**1.25 (1.21 to 1.30)**	**<0.001**	**1.19 (1.12 to 1.27)**	**<0.001**
Obese	7-8 (ref)	1.00 (reference)		1.00 (reference)		1.00 (reference)	
	< 6	**1.11 (1.08 to 1.14)**	**<0.001**	**1.13 (1.10 to 1.17)**	**<0.001**	**1.15 (1.10 to 1.22)**	**<0.001**
	6-7	0.98 (0.95 to 1.00)	0.059	**1.03 (1.00 to 1.06)**	**0.048**	1.00 (0.95 to 1.05)	0.930
	≥ 8	**1.21 (1.18 to 1.25)**	**<0.001**	**1.25 (1.22 to 1.29)**	**<0.001**	**1.22 (1.15 to 1.28)**	**<0.001**
Subjective Health-level							
High	7-8 (ref)	1.00 (reference)		1.00 (reference)		1.00 (reference)	
	< 6	**1.07 (1.03 to 1.12)**	**0.001**	**1.11 (1.05 to 1.16)**	**<0.001**	**1.13 (1.05 to 1.22)**	**0.001**
	6-7	**0.96 (0.93 to 1.00)**	**0.041**	**1.05 (1.00 to 1.09)**	**0.039**	1.01 (0.95 to 1.08)	0.749
	≥ 8	**1.10 (1.06 to 1.14)**	**<0.001**	**1.14 (1.09 to 1.19)**	**<0.001**	**1.13 (1.06 to 1.21)**	**<0.001**
Middle	7-8 (ref)	1.00 (reference)		1.00 (reference)		1.00 (reference)	
	< 6	**1.04 (1.01 to 1.07)**	**0.004**	**1.06 (1.02 to 1.09)**	**0.001**	**1.07 (1.02 to 1.12)**	**0.010**
	6-7	0.98 (0.96 to 1.00)	0.077	1.02 (0.99 to 1.05)	0.169	1.03 (0.98 to 1.07)	0.276
	≥ 8	**1.13 (1.11 to 1.16)**	**<0.001**	**1.14 (1.11 to 1.18)**	**<0.001**	**1.20 (1.14 to 1.26)**	**<0.001**
Low	7-8 (ref)	1.00 (reference)		1.00 (reference)		1.00 (reference)	
	< 6	**0.96 (0.94 to 0.98)**	**0.001**	**0.97 (0.94 to 1.00)**	**0.024**	0.99 (0.94 to 1.05)	0.709
	6-7	0.98 (0.96 to 1.01)	0.191	1.02 (0.99 to 1.05)	0.193	1.02 (0.96 to 1.09)	0.521
	≥ 8	**1.10 (1.07 to 1.13)**	**<0.001**	**1.13 (1.10 to 1.16)**	**<0.001**	**1.09 (1.03 to 1.16)**	**0.004**

Depression counseling							
Yes	7-8 (ref)	1.00 (reference)		1.00 (reference)		1.00 (reference)	
	< 6	**1.15 (1.01 to 1.30)**	**0.029**	1.15 (1.00 to 1.32)	0.051	**1.25 (1.03 to 1.52)**	**0.027**
	6-7	0.98 (0.85 to 1.13)	0.742	1.01 (0.86 to 1.19)	0.900	1.06 (0.84 to 1.33)	0.643
	≥ 8	**1.32 (1.16 to 1.51)**	**<0.001**	**1.30 (1.11 to 1.52)**	**0.001**	**1.51 (1.23 to 1.85)**	**<0.001**
No	7-8 (ref)	1.00 (reference)		1.00 (reference)		1.00 (reference)	
	< 6	**1.16 (1.14 to 1.18)**	**<0.001**	**1.15 (1.13 to 1.17)**	**<0.001**	**1.18 (1.14 to 1.22)**	**<0.001**
	6-7	1.00 (0.99 to 1.02)	0.694	1.00 (0.98 to 1.02)	0.968	0.98 (0.95 to 1.01)	0.278
	≥ 8	**1.20 (1.18 to 1.22)**	**<0.001**	**1.21 (1.19 to 1.23)**	**<0.001**	**1.18 (1.14 to 1.21)**	**<0.001**
Stress counseling							
Yes	7-8 (ref)	1.00 (reference)		1.00 (reference)		1.00 (reference)	
	< 6	**1.17 (1.05 to 1.30)**	**0.004**	**1.15 (1.03 to 1.29)**	**0.017**	**1.23 (1.03 to 1.46)**	**0.022**
	6-7	1.00 (0.89 to 1.12)	0.945	1.02 (0.89 to 1.17)	0.776	0.97 (0.79 to 1.19)	0.771
	≥ 8	**1.42 (1.26 to 1.60)**	**<0.001**	**1.41 (1.24 to 1.61)**	**<0.001**	**1.46 (1.20 to 1.78)**	**<0.001**
No	7-8 (ref)	1.00 (reference)		1.00 (reference)		1.00 (reference)	
	< 6	**1.16 (1.14 to 1.18)**	**<0.001**	**1.15 (1.13 to 1.17)**	**<0.001**	**1.17 (1.13 to 1.21)**	**<0.001**
	6-7	1.00 (0.99 to 1.02)	0.770	1.00 (0.98 to 1.02)	0.958	0.99 (0.96 to 1.02)	0.357
	≥ 8	**1.20 (1.18 to 1.22)**	**<0.001**	**1.21 (1.19 to 1.23)**	**<0.001**	**1.18 (1.14 to 1.21)**	**<0.001**
Education							
	7-8 (ref)	1.00 (reference)		1.00 (reference)		1.00 (reference)	

High school or lower education	< 6	**1.07 (1.04 to 1.09)**	**<0.001**	**1.07 (1.04 to 1.09)**	**<0.001**	**1.08 (1.03 to 1.13)**	**0.001**
	6-7	1.00 (0.97 to 1.02)	0.616	1.00 (0.98 to 1.03)	0.736	1.02 (0.97 to 1.06)	0.487
	≥ 8	**1.15 (1.12 to 1.17)**	**<0.001**	**1.16 (1.13 to 1.18)**	**<0.001**	**1.16 (1.11 to 1.21)**	**<0.001**
College or higher education	7-8 (ref)	1.00 (reference)		1.00 (reference)		1.00 (reference)	
	< 6	**1.23 (1.20 to 1.27)**	**<0.001**	**1.23 (1.19 to 1.26)**	**<0.001**	**1.24 (1.18 to 1.30)**	**<0.001**
	6-7	1.00 (0.98 to 1.02)	0.871	1.00 (0.98 to 1.03)	0.740	0.97 (0.94 to 1.02)	0.217
	≥ 8	**1.25 (1.22 to 1.28)**	**<0.001**	**1.27 (1.23 to 1.30)**	**<0.001**	**1.20 (1.14 to 1.25)**	**<0.001**
Household income							
Lowest and second quartile	7-8 (ref)	1.00 (reference)		1.00 (reference)		1.00 (reference)	
	< 6	**1.23 (1.20 to 1.27)**	**<0.001**	**1.20 (1.16 to 1.25)**	**<0.001**	**1.25 (1.19 to 1.32)**	**<0.001**
	6-7	1.01 (0.99 to 1.04)	0.368	0.98 (0.95 to 1.01)	0.105	1.01 (0.97 to 1.06)	0.633
	≥ 8	**1.22 (1.18 to 1.25)**	**<0.001**	**1.20 (1.16 to 1.24)**	**<0.001**	**1.21 (1.15 to 1.27)**	**<0.001**
Third and highest quartile	7-8 (ref)	1.00 (reference)		1.00 (reference)		1.00 (reference)	
	< 6	**1.11 (1.09 to 1.13)**	**<0.001**	**1.11 (1.09 to 1.14)**	**<0.001**	**1.10 (1.06 to 1.15)**	**<0.001**
	6-7	1.00 (0.98 to 1.02)	0.647	1.02 (1.00 to 1.04)	0.101	**0.96 (0.92 to 1.00)**	**0.037**
	≥ 8	**1.18 (1.16 to 1.21)**	**<0.001**	**1.20 (1.18 to 1.23)**	**<0.001**	**1.14 (1.10 to 1.19)**	**<0.001**
Smoking status							
Non-smoker	7-8 (ref)	1.00 (reference)		1.00 (reference)		1.00 (reference)	
	< 6	**1.22 (1.17 to 1.27)**	**<0.001**	**1.21 (1.15 to 1.26)**	**<0.001**	**1.16 (1.07 to 1.26)**	**<0.001**
	6-7	1.02 (0.98 to 1.05)	0.341	1.01 (0.97 to 1.05)	0.741	**0.90 (0.84 to 0.97)**	**0.004**
	≥ 8	**1.18 (1.13 to 1.22)**	**<0.001**	**1.18 (1.13 to 1.23)**	**<0.001**	1.08 (1.00 to 1.16)	0.062

Ex-smoker	7-8 (ref)	1.00 (reference)		1.00 (reference)		1.00 (reference)	
	< 6	**1.12 (1.08 to 1.15)**	**<0.001**	**1.13 (1.08 to 1.17)**	**<0.001**	**1.17 (1.10 to 1.25)**	**<0.001**
	6-7	0.98 (0.95 to 1.01)	0.203	1.02 (0.99 to 1.06)	0.186	1.01 (0.95 to 1.07)	0.802
	≥ 8	**1.21 (1.18 to 1.25)**	**<0.001**	**1.25 (1.20 to 1.29)**	**<0.001**	**1.21 (1.13 to 1.28)**	**<0.001**
Smoker	7-8 (ref)	1.00 (reference)		1.00 (reference)		1.00 (reference)	
	< 6	**1.14 (1.12 to 1.17)**	**<0.001**	**1.14 (1.11 to 1.16)**	**<0.001**	**1.17 (1.12 to 1.22)**	**<0.001**
	6-7	1.00 (0.98 to 1.02)	0.844	1.00 (0.97 to 1.02)	0.790	1.02 (0.97 to 1.06)	0.476
	≥ 8	**1.23 (1.20 to 1.25)**	**<0.001**	**1.23 (1.20 to 1.26)**	**<0.001**	**1.23 (1.18 to 1.28)**	**<0.001**
Alcohol consumption							
Non-drinker and 1day/month	7-8 (ref)	1.00 (reference)		1.00 (reference)		1.00 (reference)	
	< 6	**1.16 (1.14 to 1.18)**	**<0.001**	**1.15 (1.13 to 1.18)**	**<0.001**	**1.19 (1.14 to 1.23)**	**<0.001**
	6-7	1.00 (0.98 to 1.02)	0.870	1.00 (0.98 to 1.02)	0.891	1.01 (0.97 to 1.05)	0.669
	≥ 8	**1.22 (1.20 to 1.25)**	**<0.001**	**1.23 (1.21 to 1.26)**	**<0.001**	**1.21 (1.17 to 1.26)**	**<0.001**
2–9 days/month	7-8 (ref)	1.00 (reference)		1.00 (reference)		1.00 (reference)	
	< 6	**1.19 (1.15 to 1.24)**	**<0.001**	**1.17 (1.13 to 1.22)**	**<0.001**	**1.20 (1.11 to 1.29)**	**<0.001**
	6-7	1.01 (0.98 to 1.04)	0.428	1.00 (0.97 to 1.04)	0.867	**0.94 (0.88 to 1.00)**	**0.037**
	≥ 8	**1.18 (1.15 to 1.22)**	**<0.001**	**1.18 (1.14 to 1.23)**	**<0.001**	**1.12 (1.05 to 1.21)**	**0.001**
Over 10 days/month	7-8 (ref)	1.00 (reference)		1.00 (reference)		1.00 (reference)	
	< 6	**1.14 (1.08 to 1.21)**	**<0.001**	**1.14 (1.07 to 1.21)**	**<0.001**	1.11 (0.97 to 1.27)	0.132
	6-7	1.01 (0.96 to 1.06)	0.759	1.00 (0.94 to 1.06)	0.957	0.95 (0.83 to 1.08)	0.404

	≥ 8	**1.14 (1.08 to 1.21)**	**<0.001**	**1.14 (1.07 to 1.21)**	**<0.001**	1.12 (0.98 to 1.26)	0.090

Abbreviations: BMI, body mass index; CI, confidence interval; KCHS, Korea Community Health Survey.

The values in bold font represent significant variance ($p<0.05$).

[a] According to the Asian-Pacific guidelines, BMI is divided into four groups: underweight (<18.5 kg/ m^2), normal (18.5–22.9 kg/ m^2), overweight (23.0–24.9 kg/ m^2), and obese (≥25.0 kg/ m^2).

Table S7. Weighted odds ratios in the prevalence of individuals who have both hypertension and diabetes stratified by sleep duration before and during COVID–19, with 7-8 hours of sleep duration as the reference (weighted % [95% CI]).

Variables	Sleep duration (hours/day)	Overall (2009–2022)			Pre-pandemic era (2009–2019)			During pandemic era (2020–2022)		
		Sample size	wOR (95% CI)	p-value	Sample size	wOR (95% CI)	p-value	Sample size	wOR (95% CI)	p-value
Overall	7-8 (ref)	491,183	1.00 (reference)		392,601	1.00 (reference)		98,582	1.00 (reference)	
	< 6	822,197	**1.20 (1.18 to 1.23)**	**<0.001**	671,007	**1.18 (1.16 to 1.21)**	**<0.001**	151,190	**1.25 (1.20 to 1.30)**	**<0.001**
	6-7	914,450	1.00 (0.98 to 1.02)	0.725	742,258	1.00 (0.98 to 1.02)	0.820	172,192	0.99 (0.95 to 1.03)	0.640
	≥ 8	676,057	**1.19 (1.17 to 1.22)**	**<0.001**	548,619	**1.19 (1.17 to 1.22)**	**<0.001**	127,438	**1.18 (1.14 to 1.23)**	**<0.001**
Sex										
Male	7-8 (ref)	192,647	1.00 (reference)		155,797	1.00 (reference)		36,850	1.00 (reference)	
	< 6	388,490	**1.21 (1.17 to 1.24)**	**<0.001**	318,907	**1.20 (1.16 to 1.24)**	**<0.001**	69,583	**1.28 (1.21 to 1.36)**	**<0.001**
	6-7	423,760	0.99 (0.96 to 1.02)	0.463	343,821	1.02 (0.99 to 1.05)	0.198	79,939	0.98 (0.93 to 1.03)	0.402
	≥ 8	301,539	**1.22 (1.18 to 1.25)**	**<0.001**	242,616	**1.24 (1.20 to 1.28)**	**<0.001**	58,923	**1.21 (1.15 to 1.28)**	**<0.001**
Female	7-8 (ref)	298,536	1.00 (reference)		236,804	1.00 (reference)		61,732	1.00 (reference)	
	< 6	433,707	**1.16 (1.13 to 1.20)**	**<0.001**	352,100	**1.15 (1.12 to 1.18)**	**<0.001**	81,607	**1.20 (1.14 to 1.26)**	**<0.001**
	6-7	490,690	1.00 (0.97 to 1.03)	0.967	398,437	0.99 (0.96 to 1.02)	0.463	92,253	1.03 (0.98 to 1.09)	0.271
	≥ 8	374,518	**1.20 (1.16 to 1.23)**	**<0.001**	306,003	**1.19 (1.16 to 1.23)**	**<0.001**	68,515	**1.20 (1.14 to 1.27)**	**<0.001**

Age, years										
19–30	7-8 (ref)	35,462	1.00 (reference)		30,197	1.00 (reference)		5,265	1.00 (reference)	
	< 6	92,503	1.41 (0.89 to 2.21)	0.140	78,294	1.23 (0.71 to 2.12)	0.461	14,209	1.44 (0.60 to 3.45)	0.416
	6-7	125,725	1.10 (0.75 to 1.62)	0.623	101,213	1.06 (0.68 to 1.68)	0.788	24,512	0.52 (0.25 to 1.09)	0.085
	≥ 8	103,547	1.44 (0.99 to 2.10)	0.055	83,090	1.41 (0.89 to 2.25)	0.143	20,457	0.96 (0.46 to 1.98)	0.909
31–40	7-8 (ref)	46,987	1.00 (reference)		40,348	1.00 (reference)		6,639	1.00 (reference)	
	< 6	124,600	**1.63 (1.36 to 1.95)**	**<0.001**	107,607	**1.48 (1.20 to 1.84)**	**<0.001**	16,993	**1.80 (1.26 to 2.57)**	**0.001**
	6-7	155,692	1.04 (0.89 to 1.22)	0.596	133,222	0.95 (0.80 to 1.13)	0.570	22,470	0.93 (0.68 to 1.28)	0.647
	≥ 8	96,633	**1.31 (1.11 to 1.54)**	**0.002**	82,825	**1.30 (1.07 to 1.58)**	**0.009**	13,808	1.06 (0.74 to 1.52)	0.744
41–50	7-8 (ref)	70,484	1.00 (reference)		59,445	1.00 (reference)		11,039	1.00 (reference)	
	< 6	169,934	**1.52 (1.41 to 1.65)**	**<0.001**	144,514	**1.45 (1.33 to 1.59)**	**<0.001**	25,420	**1.40 (1.18 to 1.67)**	**<0.001**
	6-7	183,765	1.06 (0.98 to 1.13)	0.130	154,089	0.95 (0.88 to 1.02)	0.157	29,676	0.94 (0.81 to 1.09)	0.400
	≥ 8	97,802	**1.33 (1.23 to 1.44)**	**<0.001**	82,328	**1.28 (1.17 to 1.40)**	**<0.001**	15,474	**1.20 (1.01 to 1.42)**	**0.037**
51–60	7-8 (ref)	96,383	1.00 (reference)		78,073	1.00 (reference)		18,310	1.00 (reference)	
	< 6	172,901	**1.29 (1.23 to 1.36)**	**<0.001**	140,297	**1.27 (1.21 to 1.34)**	**<0.001**	32,604	**1.31 (1.19 to 1.44)**	**<0.001**
	6-7	176,886	1.01 (0.97 to 1.05)	0.662	143,617	1.01 (0.97 to 1.06)	0.605	33,269	0.92 (0.84 to 1.01)	0.070

Abbreviations: BMI, body mass index; CI, confidence interval; KCHS, Korea Community Health Survey.

The values in bold font represent significant variance ($p<0.05$).

[a] According to the Asian-Pacific guidelines, BMI is divided into four groups: underweight (<18.5 kg/ m^2), normal (18.5–22.9 kg/ m^2), overweight (23.0–24.9 kg/ m^2), and obese (≥25.0 kg/ m^2).

Table S8. Weighted odds ratios and 95% CI for the association between sleep duration and hypertension, diabetes, and their coexistence according to the COVID–19 pandemic, based on an interaction model.

	**Hypertension**		**Diabetes**		**Hypertension & Diabetes**	
	Weighted OR (95% CI)	p-value	Weighted OR (95% CI)	p-value	Weighted OR (95% CI)	p-value
7-8 hour sleep & before the pandemic	1.00 (ref)		1.00 (ref)		1.00 (ref)	
<6 hour sleep & before the pandemic	**1.85 (1.82, 1.87)**	**<0.001**	**1.69 (1.66, 1.72)**	**<0.001**	**1.87 (1.82, 1.91)**	**<0.001**
6-7 hour sleep & before the pandemic	**1.14 (1.13, 1.16)**	**<0.001**	**1.07 (1.05, 1.09)**	**<0.001**	**1.08 (1.05, 1.10)**	**<0.001**
>8 hour sleep & before the pandemic	**1.22 (1.21, 1.24)**	**<0.001**	**1.37 (1.35, 1.40)**	**<0.001**	**1.45 (1.42, 1.48)**	**<0.001**
7-8 hour sleep & during the pandemic	**2.53 (2.48, 2.58)**	**<0.001**	**2.48 (2.42, 2.55)**	**<0.001**	**2.84 (2.75, 2.93)**	**<0.001**
<6 hour sleep & during the pandemic	**1.56 (1.53, 1.59)**	**<0.001**	**1.61 (1.57, 1.65)**	**<0.001**	**1.66 (1.61, 1.72)**	**<0.001**
6-7 hour sleep & during the pandemic	**1.24 (1.22, 1.27)**	**<0.001**	**1.37 (1.33, 1.40)**	**<0.001**	**1.41 (1.37, 1.46)**	**<0.001**
>8 hour sleep & during the pandemic	**1.50 (1.47, 1.53)**	**<0.001**	**1.84 (1.79, 1.89)**	**<0.001**	**1.98 (1.92, 2.05)**	**<0.001**

Abbreviations: CI, confidence interval.
